# Ruthenium-Catalyzed Dehydrogenative Intermolecular O-H/Si-H/C-H Silylation: Synthesis of (*E*)-Alkenyl Silyl-Ether and Silyl-Ether Heterocycle

**DOI:** 10.3390/molecules28207186

**Published:** 2023-10-20

**Authors:** Ziwei Huang, Qiao Lin, Jiefang Li, Shanshan Xu, Shaohuan Lv, Feng Xie, Jun Wang, Bin Li

**Affiliations:** 1 School of Biotechnology and Health Sciences, Wuyi University, Jiangmen 529020, China; prayerhz@126.com (Z.H.); m19979306090@163.com (Q.L.); jenniferlee1101@163.com (J.L.); xshan1004@126.com (S.X.); 15931918925@163.com (S.L.); 2 Guangdong Wamo New Material Technology Co., Ltd., Jiangmen 529020, China; 3 Department of Chemistry, Hong Kong Baptist University, Hong Kong, China; wang.j@sustc.edu.cn; 4 Guangdong Provincial Key Laboratory of Large Animal Models for Biomedicine, Wuyi University, Jiangmen 529020, China

**Keywords:** ruthenium catalysis, silylation, C-H activation, silyl-ether, dehydrogenative coupling

## Abstract

Selective dehydrogenative silylation is one of the most valuable tools for synthesizing organosilicon compounds. In this study, a regio- and stereoselective ruthenium-catalyzed dehydrogenative intermolecular silylation was firstly developed to access (*E*)-alkenyl silyl-ether derivatives and silyl-ether heterocycles with good functional group tolerance. Furthermore, two pathways for RuH_2_(CO)(PPh_3_)_3_/NBE-catalyzed dehydrogenative intermolecular silylation of alcohols and alkenes as well as intermolecular silylation of naphthol derivatives were investigated with H_2_SiEt_2_ as the hydrosilane reagent.

## 1. Introduction

**Scheme 1:**
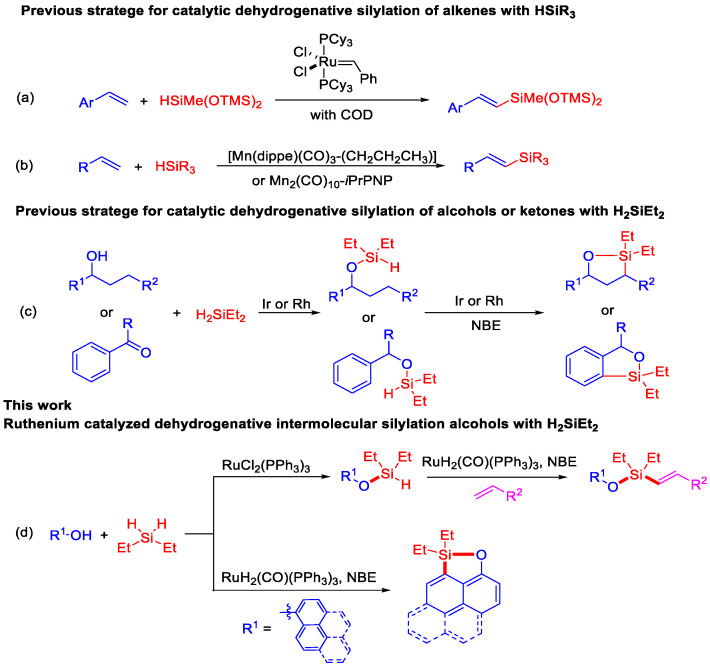
Strategy for transition metal-catalyzed dehydrogenative silylation.

Alkenylsilanes are one of the most versatile building blocks in numerous polymers, semi-conductors, and medicinal compounds due to their low toxicity and favorable metabolic profiles [1,2]. They can also serve as synthetic intermediates for useful moieties, including allylic alcohols, allylic amines, and alkenyl halides via oxidation with H_2_O_2_, aminations, and halogenations [3,4,5,6,7]. Compared to the alkenylsilanes, synthetic methodologies with the silyl–Heck reaction [8,9,10], alkyne hydrosilylation [11,12], and dehydrogenative silylation of alkenes, direct dehydrogenative silylation of alkenes has attracted attention as a competitive alternative process because alkenes are less expensive and more readily available [13,14]. The competitive alkene hydrosilylation to generate alkylsilanes is still a challenging step during the dehydrogenative silylation process to produce alkenylsilanes [15,16,17]. Recently, several precious metals, such as rhodium [18,19,20], iridium [21], ruthenium [22], and earth-abundant metals, including iron [23], copper [24], and manganese [25,26], were developed for the selective dehydrogenative silylation of alkenes. For example, Zhang reported efficient [RhCl(COD)]_2_/PPh_3_-catalyzed dehydrogenative silylation derivatives with trisubstituted hydrosilanes as the hydrosilane reagents [18]. Jeon demonstrated regio- and stereoselective dehydrogenative silylation and hydrosilylation of vinylarenes with alkoxysilanes by using a ruthenium alkylidene catalyst (Scheme 1a) [22]. Very recently, [Mn(dippe)(CO)_3_-(CH_2_CH_2_CH_3_)] developed by Kirchner [25] and the Mn_2_(CO)_10_-*i*PrPNP catalytic system by Jin Xie [26] were successfully applied in the dehydrogenative silylation of alkenes, with trisubstituted hydrosilanes as the hydrosilane reagents (Scheme 1b).

In spite of the significant progress in the direct dehydrogenative silylation of alkenes, HSiEt_3_ and HSiMe(OSiMe_3_)_2_ as the hydrosilane reagents are always used, whereas H_2_SiEt_2_ has still not been developed in dehydrogenative silylation of alkenes mainly because H_2_SiEt_2_ is more difficult to control and easily generates other silane-byproducts during the catalytic reaction. In addition, H_2_SiEt_2_ constitutes two Si–H bonds, which could provide more possibilities and molecule diversity to produce organosilicon compounds. Recently, Hartwig demonstrated efficient synthetic methods to afford five-membered silyl ether heterocycles via Rh or Ir-catalyzed dehydrogenative silylation of alcohols or ketones with H_2_SiEt_2_ (Scheme 1c) [27,28].

In our previous study, we demonstrated that the selective C–H silylation of N-heterocycles, amides, and anilides utilizing the RuHCl(CO)(PPh_3_)_3_/KOAc catalytic system was feasible to achieve silylated compounds with HSiEt_3_ [29,30,31]. Herein, we report Ru-catalyzed regio- and stereoselective dehydrogenative intermolecular silylations to synthesize (*E*)-alkenyl silyl-ether derivatives and silyl-ether heterocycles with H_2_SiEt_2_ as the hydrosilane reagent (Scheme 1d).

## 2. Results

To attempt the alkenyl silyl-ether, a two-step sequential ruthenium-catalyzed dehydrogenative intermolecular silylation of 1-phenylpropan-1-ol (**1a**) with styrene (**3a**) has been evaluated. Based on our previous experience and literature on dehydrogenative silylation of O–H/Si–H bonds [32], full conversion of intermediate silyl-ether (**2a**) was obtained from the reaction of 1-phenylpropan-1-ol (**1a**) with H_2_SiEt_2_ in the presence of 2 mol% of RuCl_2_(PPh_3_)_3_ in toluene at 60 °C under N_2_ (more details see Table S1). After 2 h, the reaction conditions of the in situ-generated silyl-ether (**2a**) with styrene (**3a**) were optimized and shown in Table 1. Firstly, 88% yield of (*E*)-alkenyl silyl-ether **4a** was observed by using 10 mol% of RuHCl(CO)(PPh_3_)_3_ as catalyst, 6 equiv. of norbornene (NBE) as hydrogen acceptor at 120 °C for 12 h (Table 1, entry 1). Then, other ruthenium complexes, such as [RuCl_2_(*p*-cymene)]_2_, [RuCl_2_(COD)]_n_, RuH_2_(CO)(PPh_3_)_3_, RuCl_2_(PPh_3_)_3_, and Ru_3_(CO)_12_ were tested for this dehydrogenative intermolecular Si–H/C–H silylation, and RuH_2_(CO)(PPh_3_)_3_ was found to the best catalyst and led to almost full conversion to (*E*)-alkenyl silyl-ether **4a** (Table 1, entry 4). Other hydrogen trappers such as cyclohexene, *t*-butyl acrylate, and *ɑ*-methylstyrene did not give better results, and the yields of (*E*)-alkenyl silyl-ether **4a** were below 44% (Table 1, entries 8–10), which is mainly because the NBE gave significant ring tension and high reactivity. Decreasing the NBE amount or the temperature did not improve the yield of (*E*)-alkenyl silyl-ether (Table 1, entries 7 and 11). Then, some other solvents, including MeOH, THF, CH_3_CN, and 1,4-dioxane, were also tested and did not show favorable effects for the reaction (Table 1, entries 12 to 15).

**Scheme 2:**
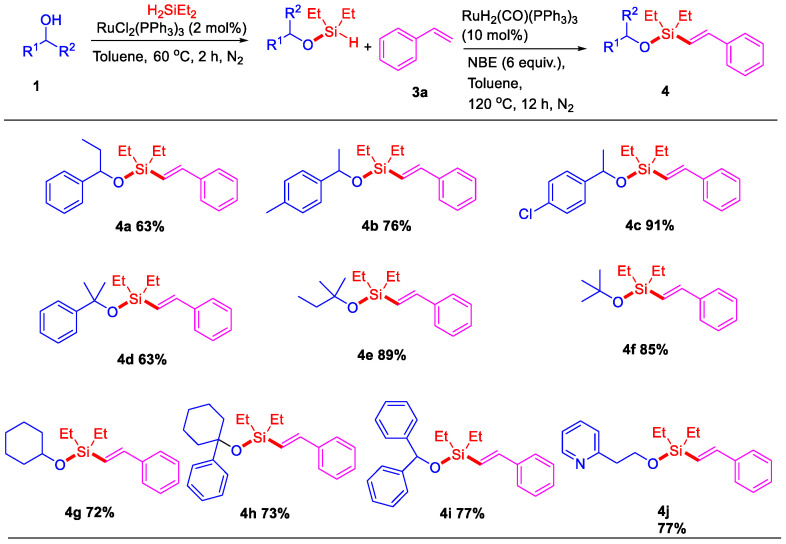
Sequential Ru-catalyzed dehydrogenative intermolecular silylation of alcohols with styrene *^a,b^*. *^a^* Reaction conditions: Step 1: 1-phenylpropan-1-ol **1** (0.5 mmol), RuCl_2_(PPh_3_)_3_ (2 mol%), H_2_SiEt_2_ (0.55 mmol), toluene (2 mL), 60 °C for 2 h, under N_2_. Step 2: styrene **3a** (0.6 mmol), RuH_2_(CO)(PPh_3_)_3_ (10 mol%), NBE (6 equiv.), at 120 °C under N_2_, 12 h. *^b^* Isolated yield.

We subsequently investigated the effect of various alcohols on the efficiency of this ruthenium-catalyzed dehydrogenative intermolecular silylation with the optimized conditions in entry 4 of Table 1 (Scheme 2). Firstly, secondary alcohols, such as 1-phenylethanol with para-Me (**3b**) and -Cl (**3c**) substituents, were applied to synthesize the corresponding (*E*)-alkenyl silyl-ethers **4b** and **4c** in 76% and 91% yields with the reaction of styrene. Tertiary alcohols with phenyl or alkyl group were able to give the desired (*E*)-alkenyl silyl-ethers **4d**–**4f** in good yields. Additionally, other functional alcohols, including cyclohexanol, 1-phenylcyclohexanol, diphenylmethanol, and 2-(pyridin-2-yl)ethanol, showed good reactivities in the production of (*E*)-alkenyl silyl-ethers (**3g**–**3j**).

**Scheme 3:**
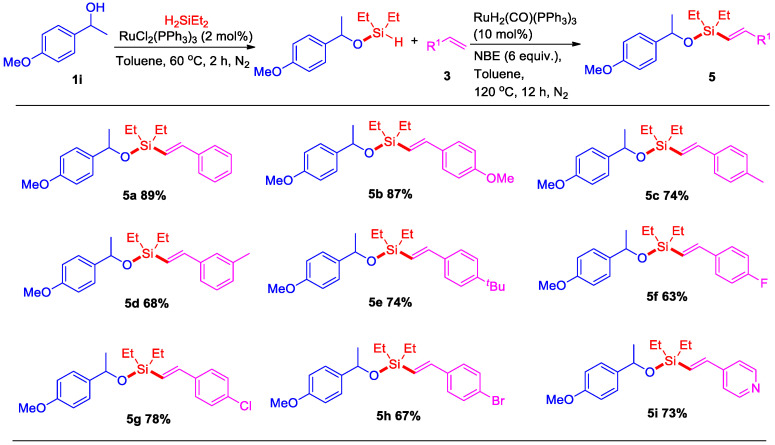
Sequential Ru-catalyzed dehydrogenative intermolecular silylation of 1-(4-methoxyphenyl)ethanol with alkenes *^a,b^*. ^*a*^ Reaction conditions: Step 1: 1-(4-methoxyphenyl)ethanol 1i (0.5 mmol), RuCl_2_(PPh_3_)_3_ (2 mol%), H_2_SiEt_2_ (0.55 mmol), toluene (2 mL), 60 °C for 2 h, under N_2_. Step 2: alkene 3 (0.6 mmol), RuH_2_(CO)(PPh_3_)_3_ (10 mol%), NBE (6 equiv.), at 120 °C under N_2_, 12 h. *^b^* Isolated yield.

We next studied the tolerance and selectivity of the reaction of alkene derivatives (Scheme 3). (*E*)-Alkenyl silyl-ethers with a 4-methoxyphenyl group could be easily prepared through this sequential ruthenium-catalyzed dehydrogenative intermolecular silylation. The corresponding alkenyl silyl-ethers bearing substituents, including -OMe, -Me, -*t*Bu, -F, -Cl, and -Br at the para or meta position on the styrene ring, could be produced in 63–87% yields under similar conditions (**5b**–**5h**). Analogously, the pyridine ring instead of the phenyl ring also proceeded well via this sequential ruthenium-catalyzed dehydrogenative intermolecular silylation.

**Scheme 4:**
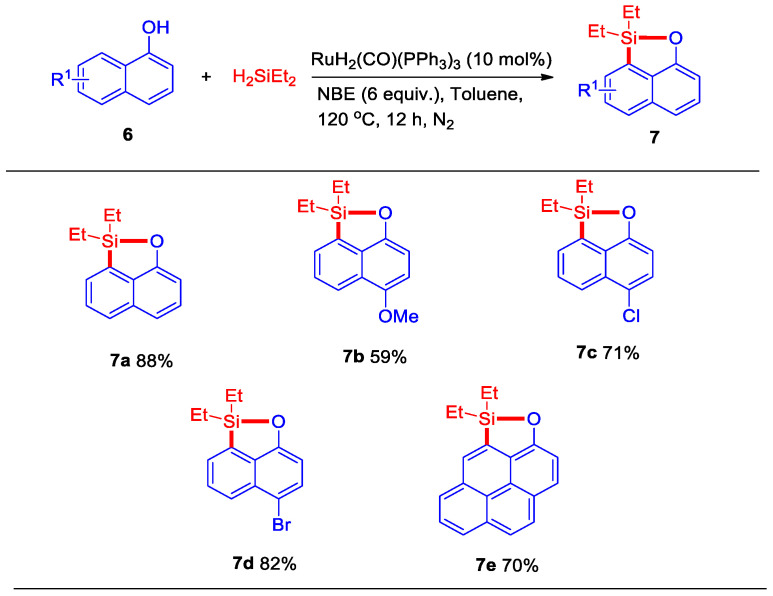
Ru-catalyzed dehydrogenative intermolecular silylation of naphthalen-1-ol derivatives *^a,b^*. *^a^* Reaction conditions: naphthalen-1-ols **6** (0.5 mmol), RuH_2_(CO)(PPh_3_)_3_ (10 mol%), NBE (6 equiv.), H_2_SiEt_2_ (0.55 mmol), toluene (2 mL), at 120 °C under N_2_, 12 h. *^b^* Isolated yield.

**Scheme 5:**
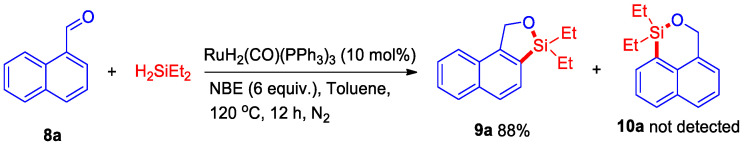
Ru-catalyzed dehydrogenative intermolecular silylation of 1-naphthaldehyde.

Then, the above-described catalytic system was evaluated for dehydrogenative C–H silylation of naphthol with styrene, however, an important silyl-ether heterocycle **7a** product was observed, which indicated that styrene did not play a role in this dehydrogenative intermolecular silylation. After our evaluation, an 88% yield of silyl-ether heterocycle **7a** was isolated in the presence of 10 mol% of RuH_2_(CO)(PPh_3_)_3_ and 6 equiv. of NBE at 120 °C for 12 h under N_2_ (Scheme 4). Then, some naphthols bearing -OMe, -Cl, and -Br substituents were also successfully transferred to the corresponding silyl-ether heterocycles **7b**–**7d** in 59–82% yields. Interestingly, this dehydrogenative intermolecular C–H silylation catalytic system could also be applied to synthesize pyren silyl-ether **7e** at a good yield. Moreover, when this reaction reacted with 1-naphthaldehyde, the C–H bond at position eight of the naphthalene ring was not activated, whereas position two of the C–H bond was activated and generated silyl-ether heterocycle **9a** at an 88% yield (Scheme 5). This result indicated that the five-membered silyl-ether heterocycle is easier to produce than the six-membered silyl-ether heterocycle.

**Scheme 6:**
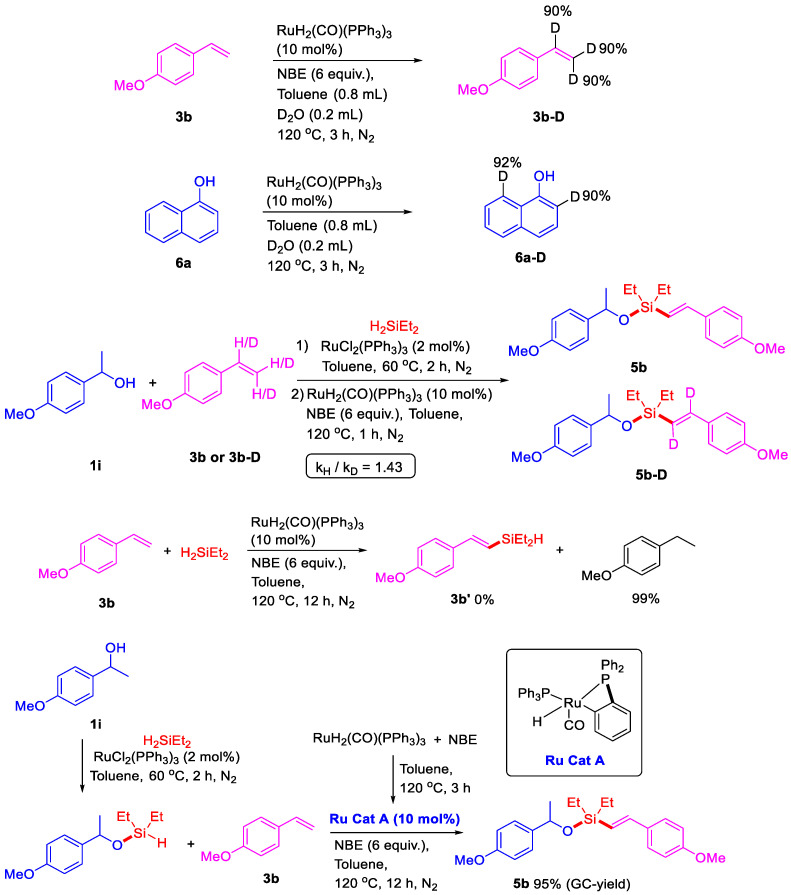
Controlled experiments.

To gain further insight into the reaction mechanism, several controlled experiments were performed (Scheme 6). Two H/D exchange reactions were conducted in the presence of D_2_O (0.2 mL) under the above conditions for 3 h. A 90% H/D exchange took place at the C–H bonds of 4-methoxystyrene **3b**. In the H/D exchange reaction of naphthol **6a**, 90% of the H/D exchange took place at position two, and a 92% H/D exchange was observed at position eight. These results indicated that C–H bonds at both position two and position eight in naphthol could be reactive. However, the ruthenium-catalyzed intermolecular silylation was performed at position eight in naphthol, not at position two, mainly because the four-membered ruthenacycle activated at position two is more difficult to generate and unstable during the catalytic cycle. In addition, the KIE value of the C–H activation in this dehydrogenative silylation of **3b** and **3b-D** with **1i** and Et_2_SiH_2_ was 1.43, revealing that the C–H cleavage is not the rate-determining step. No dehydrogenative silylated compound **3b** was detected and full conversion of reductive product 4-ethylanisole was obtained from the reaction of 4-methoxystyrene **3b** with H_2_SiEt_2_ in the presence of RuH_2_(CO)(PPh_3_)_3_ and NBE, which indicated that the silyl ether would reduce the reactivity of the Si–H bond and play an important role for dehydrogenative silylation. Furthermore, Murai described a complex Ru(H)(*o*-C_6_H_4_PPh_2_)(PPh_3_)_2_(CO), which could be easily synthesized from the reaction with RuH_2_(CO)(PPh_3_)_3_ with trimethylsilane [33]. With this important result, the reaction of RuH_2_(CO)(PPh_3_)_3_ with NBE was performed at 120 °C for 3 h, and a mixture with the major Ru complex **A** Ru(H)(*o*-C_6_H_4_PPh_2_)(PPh_3_)(CO) was obtained and detected by HR-MS (See in Supplementary Materials). Then, Ru complex **A** could be successfully applied to dehydrogenative intermolecular silylation and give 95% GC-yield of alkenyl silyl-ether **5b**. These results indicated that the Ru(H)(*o*-C_6_H_4_PPh_2_)(PPh_3_)(CO) may be the active ruthenium catalyst for the dehydrogenative intermolecular silylation.

**Scheme 7:**
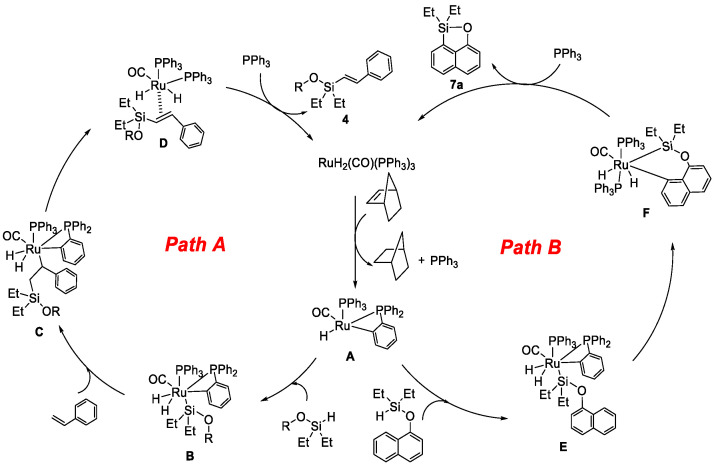
Proposed mechanism.

On the basis of the above results and previous studies [29,33,34,35] in the literature, we proposed mechanisms for the RuH_2_(CO)(PPh_3_)_3_/NBE-catalyzed silylation of alkene with alcohol and the silylation of naphthol through the intermolecular pathway, as illustrated in Scheme 7. In the Ru-catalyzed intermolecular silylation cycle of alkene with alcohol, the active Ru(H)(*o*-C_6_H_4_PPh_2_)(PPh_3_)(CO) species **A** was generated in situ after the release of one PPh_3_ and cyclometallation from the reaction of RuH_2_(CO)(PPh_3_)_3_ with NBE [29,33,34]. Then, the oxidative addition of the above-generated ROSi(Et_2_)H compound led to “Ru–Si” intermediate **B** [36]. Next, following the insertion of the Ru–Si bond to styrene [18] and *β*-elimination of Ru, intermediate **C** led to Ru intermediate **D**. Finally, (*E*)-alkenyl silyl-ether product **4** was produced via the decoordination of Ru intermediate **D,** and RuH_2_(CO)(PPh_3_)_3_ was regenerated with the previous release of PPh_3_ for the next catalytic cycle. On the other hand, in the Ru-catalyzed dehydrogenative intermolecular silylation cycle of naphthol, the active Ru(H)(*o*-C_6_H_4_PPh_2_)(PPh_3_)(CO) species **A** reacted with the in situ generated “O–Si–H” compound to afford “Ru–Si” intermediate **E**. Then, a six-membered “Ru–Si–O” intermediate **F** was generated via C–H bond deprotonation and cycloruthenation. The silyl-ether heterocycle **7a** was finally obtained by reductive elimination and released RuH_2_(CO)(PPh_3_)_3_ for the next catalytic cycle.

## 3. Materials and Methods

### 3.1. General Information

All reagents were obtained from commercial sources and used as received. Ethanol (anhydrous) was used as received. Technical grade petroleum ether (40–60 °C bp.) and ethyl acetate were used for chromatography column.

^1^H NMR spectra were recorded in CDCl_3_ at ambient temperature on Bruker AVANCE I 400 or 500 spectrometers at 400.1 or 500.1 MHz, using the solvent as internal standard (7.26 ppm). ^13^C NMR spectra were obtained at 100 MHz or 125 MHz and referenced to the internal solvent signals (central peak is 77.2 ppm). Chemical shift (δ) and coupling constants (*J*) are given in ppm and in Hz, respectively. The peak patterns are indicated as follows: s, singlet; d, doublet; t, triplet; q, quartet; m, multiplet; and br. for broad. 

GC analyses were performed with GC-7890A (Agilent, Santa Clara, CA, USA) equipped with a 30 m capillary column (HP-5ms, fused silica capillary column, 30 M × 0.25 mm × 0.25 mm film thickness) with N_2_/air as vector gas. GCMS was measured by GCMS-7890A-5975C (Agilent) with GC-7890A equipped with a 30 m capillary column (HP-5ms, fused silica capillary column, 30 M × 0.25 mm × 0.25 mm film thickness) with helium as vector gas. HRMS were measured by MAT 95XP (Termol, Vinkovci, Croatia) (LCMS-IT-TOF).

The following GC conditions were used: Method A: initial temperature 100 °C for 1.7 min, then rate 10 °C/min until 250 °C and 250 °C for 13 min. Method B: initial temperature 120 °C for 2 min, then rate 10 °C/min until 280 °C and 280 °C for 15 min.

### 3.2. General Procedure for Ruthenium-Catalyzed Dehydrogenative Intermolecular O–H/Si–H/C–H Silylation of Alcohols with Alkenes

Alcohol (0.5 mmol), RuCl_2_(PPh_3_)_3_ (2 mol%), H_2_SiEt_2_ (0.55 mmol), and toluene (2 mL) were introduced in a tube under N_2_, equipped with magnetic stirring bar and stirred at 60 °C. After 2 h, alkene (0.6 mmol), RuH_2_(CO)(PPh_3_)_3_ (10 mol%), and norbornene (3 mmol) were introduced in a tube under N_2_ and stirred at 120 °C for 12 h; then the conversion of the reaction was analyzed by gas chromatography. The solvent was then evaporated under vacuum, and the desired product was purified by using a silica gel chromatography column and a mixture of petrol ether/ethyl acetate as eluent.

### 3.3. General Procedure for Ruthenium-Catalyzed Dehydrogenative Intermolecular O–H/Si–H/C–H silylation of naphthalen-1-ol Derivatives

Naphthalen-1-ol derivative (0.5 mmol), RuH_2_(CO)(PPh_3_)_3_ (10 mol%), norbornene (3 mmol), H_2_SiEt_2_ (0.55 mmol), and toluene (2 mL) were introduced in a tube under N_2_, equipped with magnetic stirring bar and stirred at 120 °C for 12 h; then the conversion of the reaction was analyzed by gas chromatography. The solvent was then evaporated under vacuum, and the desired product was purified by using a silica gel chromatography column and a mixture of petrol ether/ethyl acetate as eluent.

### 3.4. Characterization Data of Substrates

(*E*)-diethyl(1-phenylpropoxy)(styryl)silane (**4a**) Light yellow oil, yield = 63%, 102 mg, ^1^H NMR (500 MHz, CDCl_3_): δ = 7.46 − 7.32 (m, 10H), 7.01–6.96 (m, 1H), 6.40–6.35 (m, 1H), 4.72–4.69 (m, 1H), 1.89–1.77 (m, 2H), 1.08–1.04 (m, 3H), 1.00–0.95 (m, 6H), 0.83–0.70 (m, 4H). ^13^C{^1^H} NMR (125 MHz, CDCl_3_): δ = 146.1, 145.5, 138.3, 128.6, 128.4, 128.2, 127.1, 126.7, 126.3, 124.7, 76.7, 33.6, 10.3, 6.9, 6.8, 5.7, 5.6. GC: t_R_ = 7.96 min (Method A). HRMS (EI): *m/z* calcd for C_21_H_28_NaOSi [M+Na]^+^ 347.1802, found 347.1801.

(*E*)-diethyl(styryl)(1-(p-tolyl)ethoxy)silane (**4b**) Light yellow oil, yield = 76%, 123 mg, 1H NMR (500 MHz, CDCl3): δ = 7.53 − 7.25 (m, 9H), 7.08 (d, 1H, *J* = 19.5 Hz), 6.46 (d, 1H, *J* = 19.5 Hz), 5.06–5.01 (m, 1H), 2.46 (s, 3H), 1.59 (d, 3H, *J* = 6.5 Hz), 1.16–1.07 (m, 6H), 0.90–0.79 (m, 4H). 13C{1H} NMR (125 MHz, CDCl3): δ = 146.1, 143.8, 138.2, 136.5, 129.0, 128.6, 128.4, 126.7, 125.5, 124.6, 71.0, 27.2, 21.3, 7.0, 6.9, 5.7, 5.5. GC: t_R_ = 8.07 min (Method A). HRMS (EI): *m*/*z* calcd for C_21_H_28_NaOSi [M + Na]^+^ 347.1802, found 347.1799.

(*E*)-(1-(4-chlorophenyl)ethoxy)diethyl(styryl)silane (**4c**) Light yellow oil, yield = 91%, 157 mg, ^1^H NMR (500 MHz, CDCl_3_): δ = 7.457.29 (m, 9H), 7.00 (d, 1H, *J* = 19.0 Hz), 6.36 (d, 1H, *J* = 19.5 Hz), 4.97–4.91 (m, 1H), 1.48 (d, 3H, *J* = 6.5 Hz), 1.07–0.98 (m, 6H), 0.82–0.70 (m, 4H). ^13^C{^1^H} NMR (125 MHz, CDCl_3_): δ = 146.4, 145.3, 138.1, 132.6, 128.7, 128.5, 128.4, 127.0, 126.7, 124.2, 70.5, 27.2, 6.9, 6.8, 5.6, 5.5. GC: t_R_ = 8.05 min (Method A). HRMS (EI): *m/z* calcd for C_20_H_25_ClNaOSi [M + Na]^+^ 367.1255, found 367.1260.

(*E*)-diethyl((2-phenylpropan-2-yl)oxy)(styryl)silane (**4d**) Light yellow oil, yield = 63%, 103 mg, ^1^H NMR (500 MHz, CDCl_3_): δ = 7.55–7.53 (m, 2H), 7.44–7.25 (m, 8H), 6.97 (d, 1H, *J* = 19.5 Hz), 6.38 (d, 1H, *J* = 19.5 Hz), 1.66 (s, 3H), 1.06 (t, 6H, *J* = 7.5 Hz), 0.88–0.77 (m, 4H). ^13^C{^1^H} NMR (125 MHz, CDCl_3_): δ = 150.2, 145.1, 138.4, 128.7, 128.3, 128.1, 126.8, 126.7, 126.5, 124.9, 75.4, 32.7, 7.2, 7.1. GC: t_R_ = 8.12 min (Method A). HRMS (EI): *m/z* calcd for C_21_H_28_NaOSi [M + Na]^+^ 347.1802, found 347.1798.

(*E*)-diethyl(tert-pentyloxy)(styryl)silane (**4e**) Light yellow oil, yield = 89%, 123 mg, ^1^H NMR (500 MHz, CDCl_3_): δ = 7.51 (d, 2H, *J* = 7.5 Hz), 7.41–7.30 (m, 3H), 7.02 (d, 1H, *J* = 19.0 Hz), 6.52 (d, 1H, *J* = 19.5 Hz), 1.57–1.53 (m, 2H), 1.28 (s, 6H), 1.06 (t, 6H, *J* = 7.5 Hz), 0.96 (t, 3H, *J* = 7.5 Hz), 0.80–0.76 (m, 4H). ^13^C{^1^H} NMR (125 MHz, CDCl_3_): δ = 144.8, 138.6, 128.7, 128.2, 127.4, 126.6, 74.4, 37.6, 29.6, 9.0, 7.3, 7.2. GC: t_R_ = 4.23 min (Method B). HRMS (EI): *m/z* calcd for C_17_H_28_NaOSi [M + Na]^+^ 299.1802, found 299.1800.

(*E*)-tert-butoxydiethyl(styryl)silane (**4f**) Colorless oil, yield = 85%, 111 mg, ^1^H NMR (500 MHz, CDCl_3_): δ = 7.51 (d, 2H, *J* = 7.5 Hz), 7.39 (t, 2H, *J* = 7.0 Hz), 7.32–7.29 (m, 1H), 7.02 (d, 1H, *J* = 19.0 Hz), 6.52 (d, 1H, *J* = 19.5 Hz), 1.33 (s, 9H), 1.06 (t, 6H, *J* = 7.5 Hz), 0.80–0.76 (m, 4H). ^13^C{^1^H} NMR (125 MHz, CDCl_3_): δ = 144.9, 138.6, 128.7, 128.2, 127.4, 126.7, 72.3, 32.3, 7.2, 7.1. GC: t_R_ = 4.11 min (Method B). HRMS (EI): m/z calcd for C_19_H_26_OSiK [M+K]^+^ 301.1384, found 301.1382.

(*E*)-(cyclohexyloxy)diethyl(styryl)silane (**4g**) Colorless oil, yield = 72%, 104 mg, ^1^H NMR (500 MHz, CDCl_3_): δ = 7.46 (d, 2H, *J* = 7.0 Hz), 7.34 (t, 2H, *J* = 7.5 Hz), 7.28–7.24 (m, 1H), 7.01 (d, 1H, *J* = 19.5 Hz), 6.42 (d, 1H, *J* = 19.5 Hz), 3.67–3.62 (m, 1H), 1.84–1.81 (m, 2H), 1.74–1.71 (m, 2H), 1.35–1.19 (m, 6H), 1.01 (t, 6H, *J* = 8.0 Hz), 0.77–0.72 (m, 4H). ^13^C{^1^H} NMR (125 MHz, CDCl_3_): δ = 145.9, 138.4, 128.7, 128.35, 126.7, 125.2, 71.4, 36.2, 25.7, 24.6, 7.0, 5.7. GC: t_R_ = 5.05 min (Method B). HRMS (EI): *m/z* calcd for C_18_H_28_OSiK [M+K]^+^ 327.1541, found 327.1541.

(*E*)-diethyl((1-phenylcyclohexyl)oxy)(styryl)silane (**4h**) Light yellow oil, yield = 73%, 133 mg, ^1^H NMR (500 MHz, CDCl_3_): δ = 7.56 − 7.54 (m, 2H), 7.39–7.28 (m, 8H), 6.81–6.76 (m, 1H), 6.06–6.01 (m, 1H), 2.13–2.11 (m, 2H), 1.93–1.83 (m, 4H), 1.72–1.70 (m, 1H), 1.62–1.60 (m, 2H), 1.34–1.32 (m, 1H), 1.00–0.96 (m, 6H), 0.64–0.58 (m, 4H). ^13^C{^1^H} NMR (125 MHz, CDCl_3_): δ = 148.4, 144.3, 138.6, 128.6, 128.1, 127.1, 126.8, 126.6, 126.2, 75.5, 39.2, 26.0, 22.7, 7.1, 6.6. GC: t_R_ = 10.41 min (Method A). HRMS (EI): *m/z* calcd for C_24_H_32_NaOSi [M+Na]^+^ 387.2115, found 387.2115.

(*E*)-(benzhydryloxy)diethyl(styryl)silane (**4i**) Light yellow oil, yield = 77%, 143 mg, ^1^H NMR (500 MHz, CDCl_3_): δ = 7.49 − 7.32 (m, 15H), 7.04–7.00 (m, 1H), 6.42–6.38 (m, 1H), 5.95 (s, 1H), 1.08–1.01 (m, 6H), 0.83–0.81 (m, 4H). ^13^C{^1^H} NMR (125 MHz, CDCl_3_): δ = 146.5, 145.1, 138.2, 128.7, 128.42, 128.39, 128.36, 128.3, 127.9, 127.2, 126.7, 126.6, 126.5, 124.2, 76.8, 6.9, 5.7. GC: t_R_ = 11.57 min (Method A). HRMS (EI): *m/z* calcd for C_25_H_28_NaOSi [M+Na]^+^ 395.1802, found 395.1801.

(*E*)-2-(2-((diethyl(styryl)silyl)oxy)ethyl)pyridine (**4j**) Colorless oil, yield = 77%, 120 mg, ^1^H NMR (500 MHz, CDCl_3_): δ = 8.54 − 8.53 (m, 1H), 7.64–7.61 (m, 1H), 7.43 (d, 2H, *J* = 7.0 Hz), 7.35 (d, 2H, *J* = 7.0 Hz), 7.29–7.26 (m, 2H), 7.16–7.13 (m, 1H), 6.93 (d, 1H, *J* = 19.5 Hz), 6.29 (d, 1H, *J* = 19.5 Hz), 4.07 (t, 2H, *J* = 6.5 Hz), 308 (t, 2H, *J* = 6.5 Hz), 0.97 (t, 6H, *J* = 8.0 Hz), 0.73–0.68 (m, 4H). ^13^C{^1^H} NMR (125 MHz, CDCl_3_): δ = 159.4, 149.0, 146.3, 138.2, 136.6, 128.7, 128.4, 126.7, 124.4, 124.0, 121.6, 62.8, 41.6, 6.8, 5.1. GC: t_R_ = 5.08 min (Method B). HRMS (EI): *m/z* calcd for C_19_H_26_NOSi [M+H]^+^ 312.1778, found 312.1778.

(*E*)-diethyl(1-(4-methoxyphenyl)ethoxy)(styryl)silane (**5a**) Light yellow oil, yield = 89%, 151 mg, ^1^H NMR (300 MHz, CDCl_3_): δ = 7.45 − 7.30 (m, 7H) 7.01–6.88 (m, 3H), 6.36 (d, 1H, *J* = 19.2 Hz), 5.02-4.86 (m, 1H), 3.83 (s, 3H), 1.48 (d, 3H, *J* = 6.0 Hz), 1.06–0.96 (m, 6H), 0.81–0.70 (m, 4H). ^13^C{^1^H} NMR (75 MHz, CDCl_3_): δ = 158.7, 146.1, 139.0, 138.3, 128.7, 128.4, 126.8, 126.7, 124.7, 113.7, 70.8, 55.4, 27.2, 6.9, 6.8, 5.7, 5.6. GC: t_R_ = 9.67 min (Method A). HRMS (EI): *m/z* calcd for C_21_H_28_NaO_2_Si [M+Na]^+^ 363.1751, found 363.1748.

(*E*)-diethyl(1-(4-methoxyphenyl)ethoxy)(4-methoxystyryl)silane (**5b**) Light yellow oil, yield = 87%, 161 mg, ^1^H NMR (500 MHz, CDCl_3_): δ = 7.39 − 7.31 (m, 4H), 6.94–6.88 (m, 5H), 6.19 (d, 1H, *J* = 19.5 Hz), 4.95–4.91 (m, 1H), 3.85 (s, 3H), 3.84 (s, 3H), 1.49 (d, 3H, *J* = 6.5 Hz), 1.05-0.96 (m, 6H), 0.79–0.67 (m, 4H). ^13^C{^1^H} NMR (125 MHz, CDCl_3_): δ = 159.9, 158.6, 145.6, 139.1, 131.3, 128.0, 126.8, 121.7, 114.0, 113.6, 70.6, 55.5, 55.4, 27.2, 7.0, 6.9, 5.7, 5.6. GC: t_R_ = 10.04 min (Method A). HRMS (EI): *m/z* calcd for C_22_H_31_O_3_Si [M+H]^+^ 371.2037, found 371.2036.

(*E*)-diethyl(1-(4-methoxyphenyl)ethoxy)(4-methylstyryl)silane (**5c**) Light yellow oil, yield = 74%, 131 mg, ^1^H NMR (500 MHz, CDCl_3_): δ = 7.36−7.28 (m, 4H), 7.18 (d, 2H, *J* = 7.5 Hz), 6.98–6.89 (m, 3H), 6.31 (d, 1H, *J* = 19.5 Hz), 4.96–4.92 (m, 1H), 3.84 (s, 3H), 2.39 (s, 3H), 1.49 (d, 3H, *J* = 6.5 Hz), 1.05–0.97 (m, 6H), 0.80–0.68 (m, 4H). ^13^C{^1^H} NMR (125 MHz, CDCl_3_): δ = 158.6, 146.0, 139.0, 138.3, 135.6, 129.4, 126.8, 126.6, 123.2, 113.6, 70.7, 55.4, 27.2, 21.4, 7.0, 6.9, 5.7, 5.6. GC: t_R_ = 9.61 min (Method A). HRMS (EI): *m/z* calcd for C_22_H_30_NaO_2_Si [M+Na]^+^ 377.1907, found 377.1904.

(*E*)-diethyl(1-(4-methoxyphenyl)ethoxy)(3-methylstyryl)silane (**5d**) Light yellow oil, yield = 68%, 120 mg, ^1^H NMR (500 MHz, CDCl_3_): δ = 7.36−7.34 (m, 2H), 7.30–7.28 (m, 2H), 7.16–7.15 (m, 1H), 7.00–6.92 (m, 3H), 6.38 (d, 1H, *J* = 19.5 Hz), 4.98–4.95 (m, 1H), 3.86 (s, 3H), 2.43 (s, 3H), 1.52 (d, 3H, *J* = 6.5 Hz), 1.09–1.00 (m, 6H), 0.83–0.70 (m, 4H). ^13^C{^1^H} NMR (125 MHz, CDCl_3_): δ = 158.6, 146.2, 139.0, 138.2, 129.2, 128.5, 127.4, 126.8, 126.6, 124.3, 123.9, 113.6, 70.7, 55.3, 27.2, 21.5, 7.0, 6.9, 5.6, 5.5. GC: t_R_ = 9.59 min (Method A). HRMS (EI): *m/z* calcd for C_22_H_30_NaO_2_Si [M+Na]^+^ 377.1907, found 377.1903.

(*E*)-(4-(tert-butyl)styryl)diethyl(1-(4-methoxyphenyl)ethoxy)silane (**5e**) Light yellow oil, yield = 74%, 147 mg, ^1^H NMR (500 MHz, CDCl_3_): δ = 7.39 − 7.29 (m, 6H), 6.96–6.88 (m, 3H), 6.30 (d, 1H, *J* = 19.5 Hz), 4.93–4.89 (m, 1H), 3.83 (s, 3H), 1.47 (d, 3H, *J* = 6.0 Hz), 1.35 (s, 9H), 1.03–0.94 (m, 6H), 0.78–0.66 (m, 4H). ^13^C{^1^H} NMR (125 MHz, CDCl_3_): δ = 158.6, 151.6, 145.9, 139.0, 135.6, 126.8, 126.4, 125.6, 123.6, 113.6, 70.7, 55.4, 34.8, 31.5, 27.2, 7.0, 6.9, 5.7, 5.6. GC: t_R_ = 10.29 min (Method A). HRMS (EI): *m/z* calcd for C_25_H_36_NaO_2_Si [M+Na]^+^ 419.2377, found 419.2373.

(*E*)-diethyl(4-fluorostyryl)(1-(4-methoxyphenyl)ethoxy)silane (**5f**) Light yellow oil, yield = 63%, 113 mg, ^1^H NMR (500 MHz, CDCl_3_): δ = 7.39−7.37 (m, 2H), 7.31–7.28 (m, 2H), 7.06–7.02 (m, 2H), 6.92–6.87 (m, 3H), 6.23 (d, 1H, *J* = 19.0 Hz), 4.93-4.89 (m, 1H), 3.82 (s, 3H), 1.47 (d, 3H, *J* = 6.0 Hz), 1.02–0.95 (m, 6H), 0.77–0.67 (m, 4H). ^13^C{^1^H} NMR (125 MHz, CDCl_3_): δ = 161.9 (*J*_CF_ = 246.3 Hz), 158.7, 144.8, 138.9, 134.5 (*J*_CF_ = 3.4 Hz), 128.3 (*J*_CF_ = 8.0 Hz), 126.8, 124.4 (*J*_CF_ = 2.3 Hz), 115.6 (*J*_CF_ = 21.1 Hz), 113.6, 70.8, 55.4, 27.2, 7.0, 6.9, 5.6, 5.5. ^19^F NMR (470 MHz, CDCl_3_): δ = 113.6 Hz. GC: t_R_ = 8.76 min (Method A). HRMS (EI): *m/z* calcd for C_21_H_27_FNaO_2_Si [M+Na]^+^ 381.1657, found 381.1651.

(*E*)-(4-chlorostyryl)diethyl(1-(4-methoxyphenyl)ethoxy)silane (**5g**) Light yellow oil, yield = 78%, 146 mg, ^1^H NMR (500 MHz, CDCl_3_): δ = 7.35−7.28 (m, 6H), 6.90–6.86 (m, 3H), 6.30 (d, 1H, *J* = 19.5 Hz), 4.93–4.89 (m, 1H), 3.83 (s, 3H), 1.47 (d, 3H, *J* = 6.5 Hz), 1.03–0.95 (m, 6H), 0.78–0.66 (m, 4H). ^13^C{^1^H} NMR (125 MHz, CDCl_3_): δ = 158.7, 144.7, 138.9, 136.7, 134.0, 128.8, 127.9, 126.8, 125.6, 113.6, 70.8, 55.4, 27.2, 6.9, 6.8, 5.6, 5.5. GC: t_R_ = 9.63 min (Method A). HRMS (EI): *m/z* calcd for C_21_H_27_ClNaO_2_Si [M+Na]^+^ 397.1361, found 397.1360.

(*E*)-(4-bromostyryl)diethyl(1-(4-methoxyphenyl)ethoxy)silane (**5h**) Light yellow oil, yield = 67%, 140 mg, ^1^H NMR (500 MHz, CDCl_3_): δ = 7.48 (d, 2H, *J* = 8.5 Hz) 7.32–7.27 (m, 4H), 6.90–6.86 (m, 3H), 6.33 (d, 1H, *J* = 19.5 Hz), 4.94–4.90 (m, 1H), 3.83 (s, 3H), 1.49 (d, 3H, *J* = 6.0 Hz), 1.07–0.97 (m, 6H), 0.81–0.67 (m, 4H). ^13^C{^1^H} NMR (125 MHz, CDCl_3_): δ = 158.7, 144.7, 138.8, 137.2, 131.7, 128.2, 126.8, 125.8, 122.2, 113.6, 70.8, 55.4, 27.2, 6.9, 6.8, 5.6, 5.5. GC: t_R_ = 9.95 min (Method A). HRMS (EI): *m/z* calcd for C_21_H_27_BrNaO_2_Si [M+Na]^+^ 441.0856, found 441.0853.

(*E*)-4-(2-(diethyl(1-(4-methoxyphenyl)ethoxy)silyl)vinyl)pyridine (**5i**) Light yellow oil, yield = 73%, 124 mg, ^1^H NMR (500 MHz, CDCl_3_): δ = 8.57 (d, 2H, *J* = 5.5 Hz), 7.29–7.23 (m, 4H), 6.88–6.82 (m, 3H), 6.55 (d, 1H, *J* = 19.5 Hz), 4.91-4.87 (m, 1H), 3.81 (s, 3H), 1.47 (d, 3H, *J* = 6.0 Hz), 1.01–0.95 (m, 6H), 0.78-0.68 (m, 4H). ^13^C{^1^H} NMR (125 MHz, CDCl_3_): δ = 158.8, 150.2, 145.2, 143.3, 138.7, 131.2, 126.8, 121.1, 113.7, 71.0, 55.4, 27.2, 6.9, 6.8, 5.5, 5.4. GC: t_R_ = 9.73 min (Method A). HRMS (EI): *m/z* calcd for C_20_H_28_NO_2_Si [M+H]^+^ 342.1884, found 342.1882.

2,2-diethyl-2H-naphtho [1,8-cd][1,2]oxasilole (**7a**) Light yellow oil, yield = 88%, 115 mg, ^1^H NMR (500 MHz, CDCl_3_): δ = 7.77 (d, 1H, *J* = 8.0 Hz), 7.60 (d, 1H, *J* = 6.5 Hz), 7.48-7.46 (m, 1H), 7.32–7.26 (m, 2H), 6.83–6.81 (m, 1H), 0.97–0.91 (m, 10H). ^13^C{^1^H} NMR (125 MHz, CDCl_3_): δ = 159.1, 134.3, 132.4, 132.1, 129.3, 128.1, 128.0, 127.5, 118.0, 106.9, 6.4, 6.2. GC: t_R_ = 4.37 min (Method B). HRMS (EI): *m/z* calcd for C_14_H_17_OSi [M+H]^+^ 229.1043, found 229.1042.

2,2-diethyl-6-methoxy-2H-naphtho [1,8-cd][1,2]oxasilole (**7b**) Light yellow oil, yield = 59%, 76 mg, ^1^H NMR (500 MHz, CDCl_3_): δ = 8.23 − 8.21 (m, 1H), 7.77–7.76 (m, 1H), 7.63–7.60 (m, 1H), 6.84 (d, 1H, *J* = 8.0 Hz), 6.72 (d, 1H, *J* = 8.5 Hz), 3.99 (s, 3H), 1.09–1.03 (m, 10H). ^13^C{^1^H} NMR (125 MHz, CDCl_3_): δ = 152.7, 148.8, 134.9, 131.8, 129.9, 126.9, 124.4, 123.2, 105.4, 104.9, 55.9, 6.5, 6.2. GC: t_R_ = 8.25 min (Method A). HRMS (EI): *m/z* calcd for C_15_H_19_O_2_Si [M+H]^+^ 259.1149, found 259.1149.

6-chloro-2,2-diethyl-2H-naphtho [1,8-cd][1,2]oxasilole (**7c**) Light yellow oil, yield = 71%, 93 mg, ^1^H NMR (500 MHz, CDCl_3_): δ = 8.18−8.17 (m, 1H), 7.78–7.68 (m, 2H), 7.45 (d, 1H, *J* = 8.0 Hz), 6.84 (d, 1H, *J* = 8.0 Hz), 1.08–1.00 (m, 10H). ^13^C{^1^H} NMR (125 MHz, CDCl_3_): δ = 158.2, 135.1, 132.6, 130.1, 129.8, 128.4, 127.6, 125.3, 121.2, 107.2, 6.3, 6.2. GC: t_R_ = 7.29 min (Method A). HRMS (EI): *m/z* calcd for C_14_H_16_ClOSi [M+H]^+^ 263.0653, found 263.0648.

6-bromo-2,2-diethyl-2H-naphtho [1,8-cd][1,2]oxasilole (**7d**) Light yellow oil, yield = 82%, 126 mg, ^1^H NMR (500 MHz, CDCl_3_): δ = 8.14−8.12 (m, 1H), 7.77–7.65 (m, 3H), 6.83 (d, 2H, *J* = 8.0 Hz), 1.09–1.01 (m, 10H). ^13^C{^1^H} NMR (125 MHz, CDCl_3_): δ = 158.9, 135.3, 132.7, 131.2, 131.1, 130.2, 128.7, 127.7, 110.7, 108.0, 6.3, 6.2. GC: t_R_ = 7.79 min (Method A). HRMS (EI): *m/z* calcd for C_14_H_16_BrOSi [M+H]^+^ 307.1498, found 307.1502.

4,4-diethyl-4H-pyreno [10,1-cd][1,2]oxasilole (**7e**) Light yellow oil, yield = 70%, 106 mg, ^1^H NMR (500 MHz, CDCl_3_): δ = 8.33 (s, 1H), 8.21-8.12 (m, 3H), 8.04-8.02 (m, 2H), 7.92 (d, 1H, *J* = 9.0 Hz), 7.63 (d, 1H, *J* = 8.5 Hz), 1.21-1.13 (m, 10H). ^13^C{^1^H} NMR (125 MHz, CDCl_3_): δ = 157.3, 132.5, 131.8, 131.1, 131.0, 127.5, 127.1, 126.5, 126.2, 125.6, 124.8, 124.6, 124.4, 123.5, 111.4, 6.5, 6.3. GC: t_R_ = 14.09 min (Method A). HRMS (EI): *m/z* calcd for C_20_H_19_OSi [M+H]^+^ 303.1199, found 303.1201.

3,3-diethyl-1,3-dihydronaphtho [2,1-c][1,2]oxasilole (**9a**) Light yellow oil, yield = 88%, 106 mg, ^1^H NMR (500 MHz, CDCl_3_): δ = 7.95−7.93 (m, 1H), 7.82 (d, 1H, *J* = 8.0 Hz), 7.74–7.72 (m, 1H), 7.64 (d, 1H, *J* = 8.0 Hz), 7.58–7.56 (m, 2H), 1.02–0.90 (m, 10H). ^13^C{^1^H} NMR (125 MHz, CDCl_3_): δ = 148.1, 134.2, 130.6, 128.8, 128.1, 127.5, 127.4, 126.7, 126.5, 123.2, 7.8, 7.3, 6.7. GC: t_R_ = 5.68 min (Method A). HRMS (EI): *m/z* calcd for C_15_H_19_OSi [M+H]^+^ 243.1199, found 243.1203.

## 4. Conclusions

In summary, we have described a highly regio- and stereoselective sequential ruthenium-catalyzed dehydrogenative intermolecular silylation to access (*E*)-alkenyl silyl-ether derivatives with alcohols and alkenes and H_2_SiEt_2_ as the hydrosilane reagent. Furthermore, a regioselective intermolecular C-H silylation of naphthol derivatives with H_2_SiEt_2_ to synthesize silyl-ether heterocycles was also developed using the RuH_2_(CO)(PPh_3_)_3_/NBE catalytic system. Various (*E*)-alkenyl silyl-ether derivatives and silyl-ether heterocycles bearing tolerated functional groups were successfully synthesized in moderate to excellent yields. Additionally, two pathways of Ru-catalyzed dehydrogenative intermolecular silylations were proposed.

## Figures and Tables

**Table 1:**
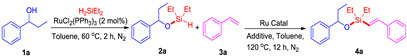
Optimization of reaction conditions for Ru-catalyzed dehydrogenative intermolecular silylation of 1-phenylpropan-1-ol (1a) with styrene (3a) ^[a]^.

Entry	Ru Catalyst	Additive	Solvent	Yield (%)
1	RuHCl(CO)(PPh_3_)_3_	NBE	toluene	88
2	[RuCl_2_(*p*-cymene)]_2_	NBE	toluene	50
3	[RuCl_2_(COD)]_n_	NBE	toluene	12
4	RuH_2_(CO)(PPh_3_)_3_	NBE	toluene	98
5	RuCl_2_(PPh_3_)_3_	NBE	toluene	53
6	Ru_3_(CO)_12_	NBE	toluene	42
7	RuH_2_(CO)(PPh_3_)_3_	NBE	toluene	74 ^b^
8	RuH_2_(CO)(PPh_3_)_3_	cyclohexene	toluene	7
9	RuH_2_(CO)(PPh_3_)_3_	*t*-butyl acrylate	toluene	11
10	RuH_2_(CO)(PPh_3_)_3_	ɑ-methylstyrene	toluene	44
11	RuH_2_(CO)(PPh_3_)_3_	NBE	toluene	15 ^c^
12	RuH_2_(CO)(PPh_3_)_3_	NBE	MeOH	trace ^d^
13	RuH_2_(CO)(PPh_3_)_3_	NBE	THF	trace ^d^
14	RuH_2_(CO)(PPh_3_)_3_	NBE	CH_3_CN	trace ^c^
15	RuH_2_(CO)(PPh_3_)_3_	NBE	1,4-dioxane	13 ^e^

**^a^** Reaction conditions: Step 1: 1-phenylpropan-1-ol **1a** (0.5 mmol), RuCl_2_ (PPh_3_)_3_ (2 mol%), H_2_SiEt_2_ (0.55 mmol), toluene (2 mL), 60 °C for 2 h, under N_2_. Step 2: styrene **3a** (0.6 mmol), Ru catalyst (10 mol%), additive (6 equiv.), at 120 °C under N_2_, 12 h, GC yield. ^b^ 3 equiv. of NBE. ^c^ at 80 °C. ^d^ at 60 °C. ^e^ at 100 °C.

## Supplementary Materials

The following supporting information can be downloaded at: https://www.mdpi.com/article/10.3390/molecules28207186/s1. Table S1. Optimization of reaction conditions for Ru catalyzed dehydrogenative silylation of 1-phenylpropan-1-ol (1a) with EtSiH_2_^[a]^.

## Sample Availability

Samples of the compounds are not available from the authors.
