# Supplementary material for: Ruthenium-Catalyzed Dehydrogenative Intermolecular O-H/Si-H/C-H Silylation: Synthesis of (E)-Alkenyl Silyl-Ether and Silyl-Ether Heterocycle
Source: Molecules. 2023 Oct 20;28(20):7186. doi: 10.3390/molecules28207186 (PMC10609488; doi:10.3390/molecules28207186)

Electronic Supporting Information

Ruthenium-Catalyzed Dehydrogenative Intermolecular O-H/Si-H/C-H Silylation: Synthesis of (*E*)-Alkenyl Silyl-ether and Silyl-ether Heterocycle

Ziwei Huang,[1,2†] Qiao Lin,[1†] Jiefang Li,[1] Shanshan Xu,[1] Shaohuan Lv,[1] Feng Xie,[1*] Jun Wang[3] and Bin Li[1,4*]

[1]*School of Biotechnology and Health Sciences, Wuyi University, Jiangmen 529020, China*
[2]*Guangdong Wamo New Material Technology Co., Ltd, Jiangmen 529020, China*
[3]*Department of Chemistry, Hong Kong Baptist University, Hong Kong, China*
[4]*Guangdong Provincial Key Laboratory of Large Animal Models for Biomedicine, Wuyi University, Jiangmen 529020, China*

Table of Contents

S2 ***General remarks***

S2 ***General procedures for dehydrogenative intermolecular O-H/Si-H/C-H silylation***

S3 ***Optimization of reaction conditions***

S5 ***H/D exchange experiments***

S7 ***The HR-MS spectra of the complex A***

S8 ***Characterization data of substrates***

S16 ***^{1}H and ^{13}C NMR Spectra***

General remarks

All reagents were obtained from commercial sources and used as received. Ethanol (anhydrous) were used as received. Technical grade petroleum ether (40-60°C bp.) and ethyl acetate were used for chromatography column.

^{1}H NMR spectra were recorded in $CDCl_3$ at ambient temperature on Bruker AVANCE I 400 or 500 spectrometers at 400.1 or 500.1 MHz, using the solvent as internal standard (7.26 ppm). ^{13}C NMR spectra were obtained at 100 MHz or 125 MHz and referenced to the internal solvent signals (central peak is 77.2 ppm). Chemical shift (δ) and coupling constants (J) are given in ppm and in Hz, respectively. The peak patterns are indicated as follows: s, singlet; d, doublet; t, triplet; q, quartet; m, multiplet, and br. for broad.

GC analyses were performed with GC-7890A (Agilent) equipped with a 30-m capillary column (HP-5ms, fused silica capillary column, 30 M*0.25 mm*0.25 mm film thickness), was used with N_2/air as vector gas. GCMS were measured by GCMS-7890A-5975C (Agilent) with GC-7890A equipped with a 30-m capillary column (HP-5ms, fused silica capillary column, 30 M*0.25 mm*0.25 mm film thickness), was used with helium as vector gas. HRMS were measured by MAT 95XP (Termol) (LCMS-IT-TOF).

The following GC conditions were used: Method A: initial temperature 100 °C, for 1.7 minutes, then rate 10 °C/min. until 250 °C and 250°C for 13 minutes. Method B: initial temperature 120 °C, for 2 minutes, then rate 10 °C/min. until 280 °C and 280°C for 15 minutes.

General procedure for ruthenium catalyzed dehydrogenative intermolecular O-H/Si-H/C-H silylation of alcohols with alkenes

Alcohol (0.5 mmol), $RuCl_2(PPh_3)_3$ (2 mol%), H_2SiEt_2 (0.55 mmol), toluene (2 mL) were introduced in a tube under N_2, equipped with magnetic stirring bar and was stirred at 60 °C. After 2 h, alkene (0.6 mmol), $RuH_2(CO)(PPh_3)_3$ (10 mol%), norbornene (3 mmol), were introduced in a tube under N_2, and was stirred at 120 °C for 12 h, then the conversion of the reaction was analyzed by gas chromatography. The solvent was then evaporated under vacuum and the desired product was purified by using a silica gel chromatography column and a mixture of petrol ether/ethyl acetate as eluent.

General procedure for ruthenium catalyzed dehydrogenative intermolecular O-H/Si-H/C-H silylation of naphthalen-1-ol derivatives

Naphthalen-1-ol derivative (0.5 mmol), $RuH_2(CO)(PPh_3)_3$ (10 mol%), norbornene (3 mmol), H_2SiEt_2 (0.55 mmol), toluene (2 mL) were introduced in a tube under N_2, equipped with magnetic stirring bar and was stirred at 120 °C for 12 h, then the conversion of the reaction was analyzed by gas chromatography. The solvent was

then evaporated under vacuum and the desired product was purified by using a silica gel chromatography column and a mixture of petrol ether/ethyl acetate as eluent.

Table S1. Optimization of reaction conditions for Ru catalyzed dehydrogenative silylation of 1-phenylpropan-1-ol (1a) with $EtSiH_2$ [a]

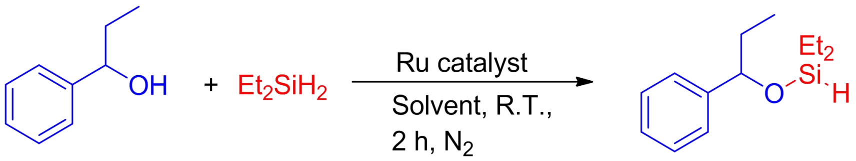

1a **2a**

Entry	Ru Catalyst	Solvent	Yield of 2a (%)
1	$RuHCl(CO)(PPh_3)_3$	toluene	73
2	$[RuCl_2(p\text{-cymene})]_2$	toluene	49
3	$[RuCl_2(COD)]_n$	toluene	51
4	$RuH_2(CO)(PPh_3)_3$	toluene	90
5	$RuCl_2(PPh_3)_3$	toluene	95
6	$RuCl_2(PPh_3)_3$	DMF	trace
7	$RuCl_2(PPh_3)_3$	1,4-dioxane	26
8	$RuCl_2(PPh_3)_3$	toluene	99[b]

[a] Reaction conditions: 1-phenylpropan-1-ol **1a** (0.5 mmol), Ru catalyst (2 mol%), H_2SiEt_2 (0.55 mmol), solvent (2 mL), room temperature for 2 h, under N_2. [b] at 60 ^{o}C.

H/D exchange experiment of 4-methoxystyrene 3b.

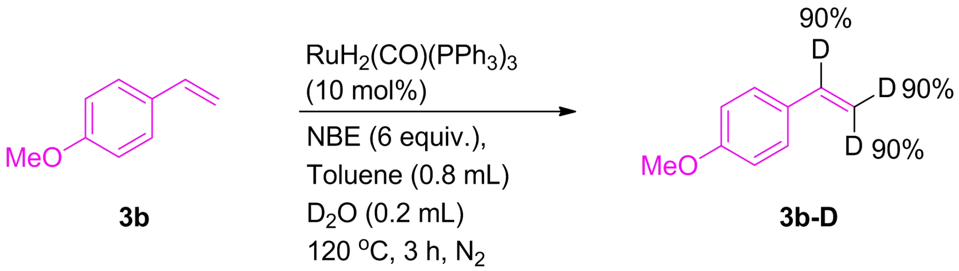

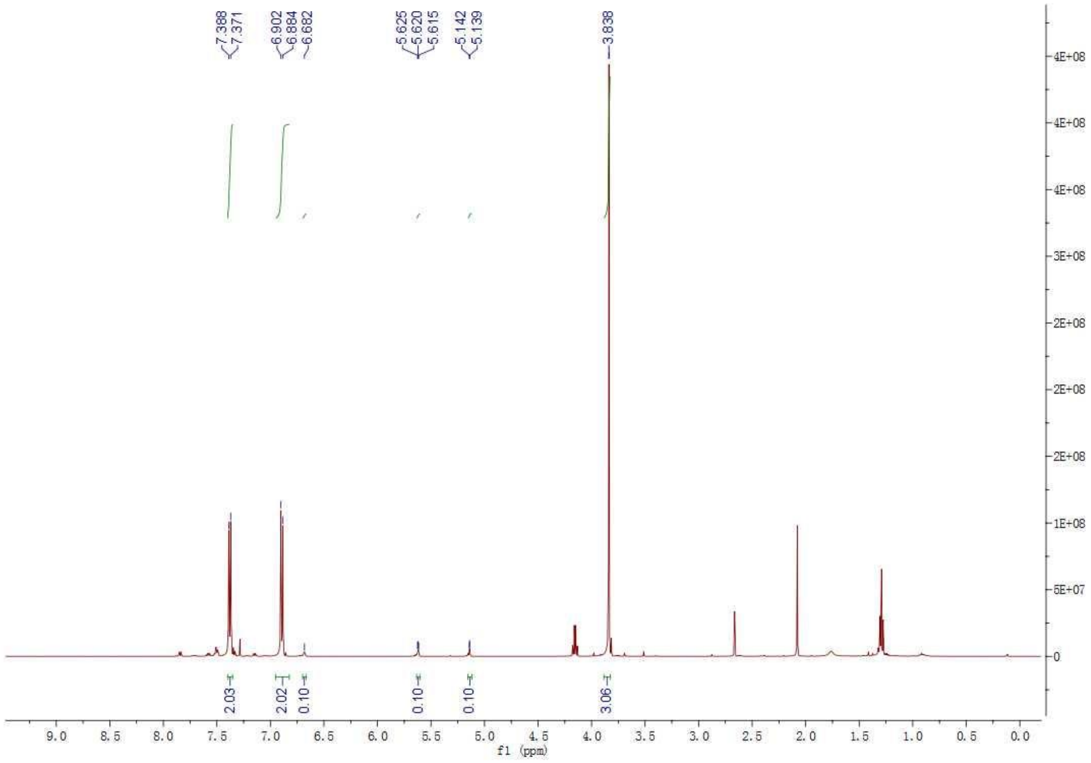

H/D exchange experiment of naphthol 6a.

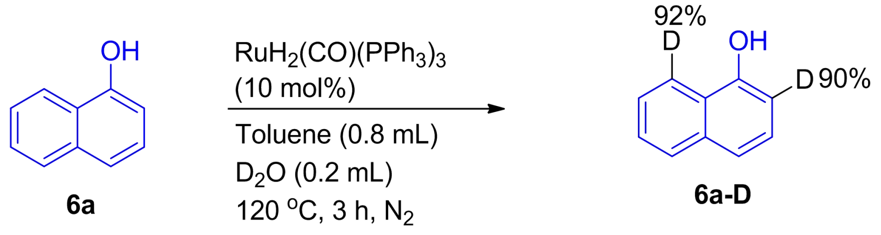

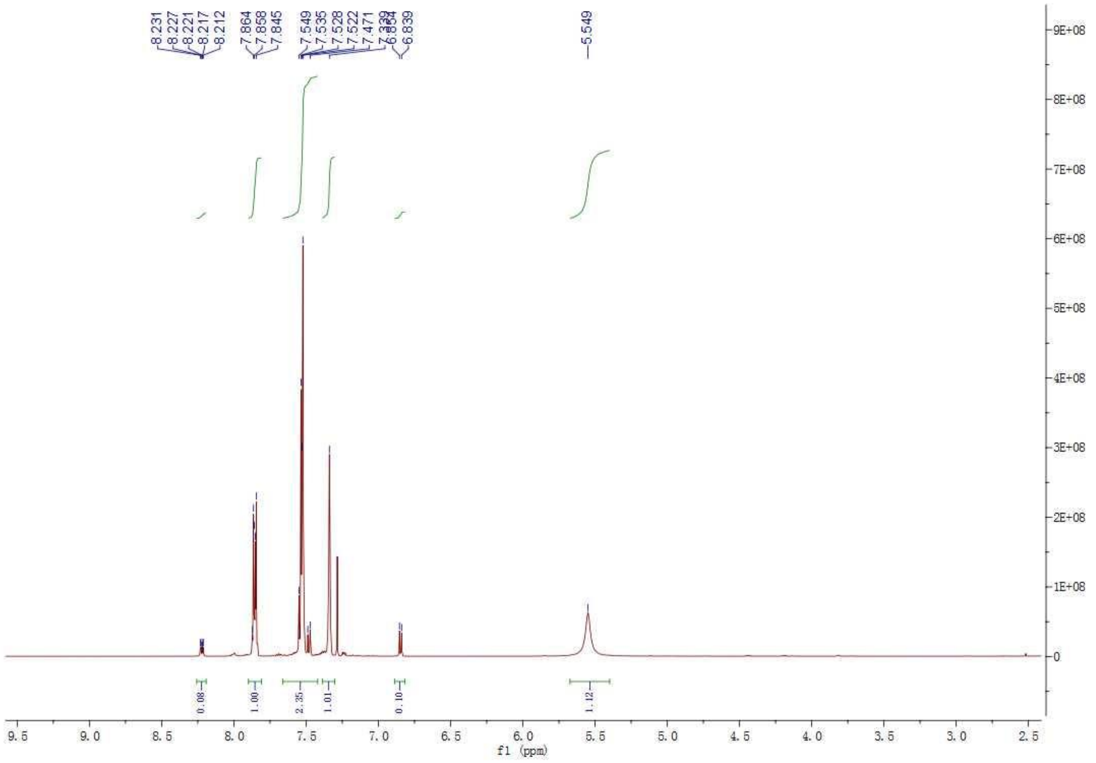

The HR-MS spectra of the complex A Ru(H)(o-$C_6H_4PPh_2$)(PPh_3)(CO)

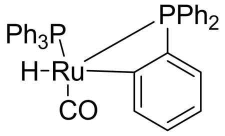

HRMS (EI): *m/z* calcd for $C_{37}H_{31}OP_2Ru$ $[M+H]^+$ 655.0888, found 655.0888.

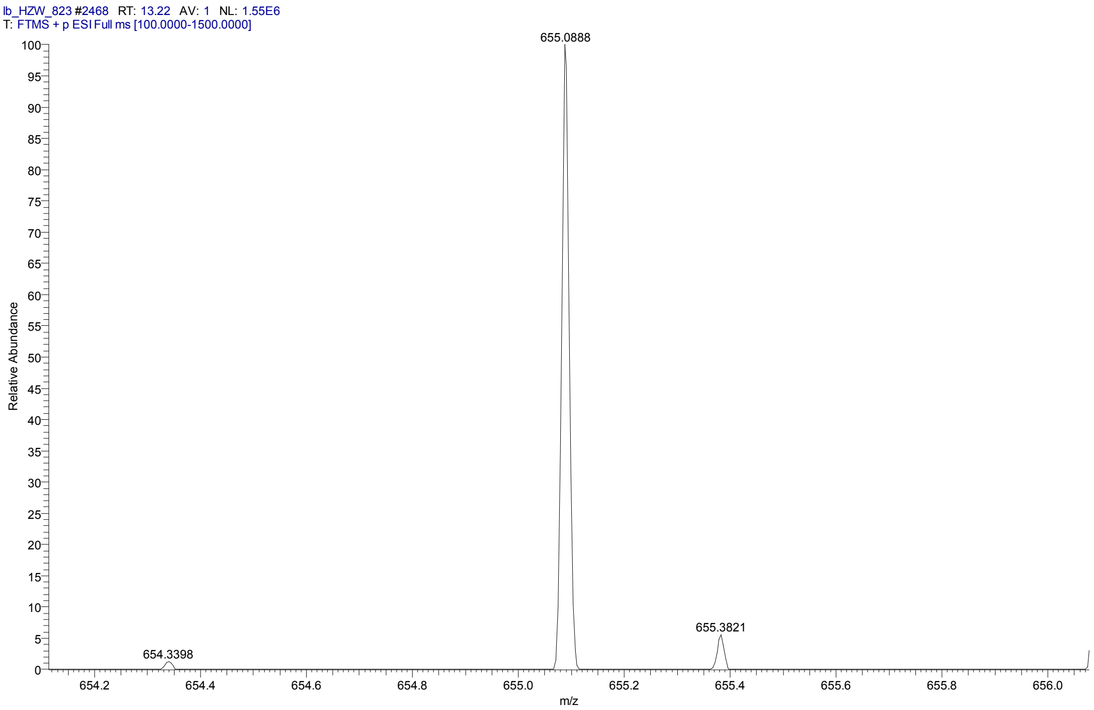

Characterization data of substrates

(E)-diethyl(1-phenylpropoxy)(styryl)silane (4a)

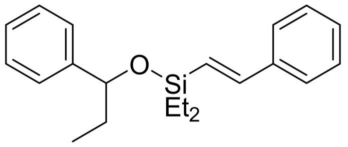

Light yellow oil, yield = 63%, 102 mg, ^{1}H NMR (500 MHz, $CDCl_3$): δ = 7.46-7.32 (m, 10H), 7.01-6.96 (m, 1H), 6.40-6.35 (m, 1H), 4.72-4.69 (m, 1H), 1.89-1.77 (m, 2H), 1.08-1.04 (m, 3H), 1.00-0.95 (m, 6H), 0.83-0.70 (m, 4H). ^{13}C{^{1}H} NMR (125 MHz, $CDCl_3$): δ = 146.1, 145.5, 138.3, 128.6, 128.4, 128.2, 127.1, 126.7, 126.3, 124.7, 76.7, 33.6, 10.3, 6.9, 6.8, 5.7, 5.6. GC: t_R = 7.96 min (Method A). HRMS (EI): *m/z* calcd for $C_{21}H_{28}NaOSi$ $[M+Na]^+$ 347.1802, found 347.1801.

(E)-diethyl(styryl)(1-(p-tolyl)ethoxy)silane (4b)

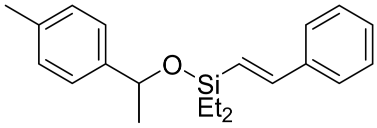

Light yellow oil, yield = 76%, 123 mg, ^{1}H NMR (500 MHz, $CDCl_3$): δ = 7.53-7.25 (m, 9H), 7.08 (d, 1H, *J* = 19.5 Hz), 6.46 (d, 1H, *J* = 19.5 Hz), 5.06-5.01 (m, 1H), 2.46 (s, 3H), 1.59 (d, 3H, *J* = 6.5 Hz), 1.16-1.07 (m, 6H), 0.90-0.79 (m, 4H). ^{13}C{^{1}H} NMR (125 MHz, $CDCl_3$): δ = 146.1, 143.8, 138.2, 136.5, 129.0, 128.6, 128.4, 126.7, 125.5, 124.6, 71.0, 27.2, 21.3, 7.0, 6.9, 5.7, 5.5. GC: t_R = 8.07 min (Method A). HRMS (EI): *m/z* calcd for $C_{21}H_{28}NaOSi$ $[M+Na]^+$ 347.1802, found 347.1799.

(E)-(1-(4-chlorophenyl)ethoxy)diethyl(styryl)silane (4c)

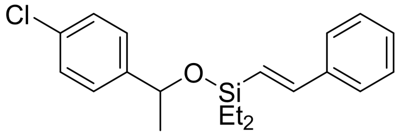

Light yellow oil, yield = 91%, 157 mg, ^{1}H NMR (500 MHz, $CDCl_3$): δ = 7.45-7.29 (m, 9H), 7.00 (d, 1H, *J* = 19.0 Hz), 6.36 (d, 1H, *J* = 19.5 Hz), 4.97-4.91 (m, 1H), 1.48 (d, 3H, *J* = 6.5 Hz), 1.07-0.98 (m, 6H), 0.82-0.70 (m, 4H). ^{13}C{^{1}H} NMR (125 MHz, $CDCl_3$): δ = 146.4, 145.3, 138.1, 132.6, 128.7, 128.5, 128.4, 127.0, 126.7, 124.2, 70.5, 27.2, 6.9, 6.8, 5.6, 5.5. GC: t_R = 8.05 min (Method A). HRMS (EI): *m/z* calcd for $C_{20}H_{25}ClNaOSi$ $[M+Na]^+$ 367.1255, found 367.1260.

(E)-diethyl((2-phenylpropan-2-yl)oxy)(styryl)silane (4d)

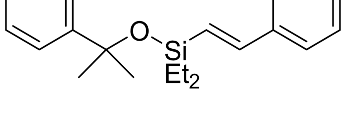

Light yellow oil, yield = 63%, 103 mg, ^{1}H NMR (500 MHz, $CDCl_3$): δ = 7.55-7.53 (m, 2H), 7.44-7.25 (m, 8H), 6.97 (d, 1H, *J* = 19.5 Hz), 6.38 (d, 1H, *J* = 19.5 Hz), 1.66 (s, 3H), 1.06 (t, 6H, *J* = 7.5 Hz), 0.88-0.77 (m, 4H). $^{13}C\{^1H\}$ NMR (125 MHz, $CDCl_3$): δ = 150.2, 145.1, 138.4, 128.7, 128.3, 128.1, 126.8, 126.7, 126.5, 124.9, 75.4, 32.7, 7.2, 7.1. GC: t_R = 8.12 min (Method A). HRMS (EI): *m/z* calcd for $C_{21}H_{28}NaOSi$ $[M+Na]^+$ 347.1802, found 347.1798.

(E)-diethyl(tert-pentyloxy)(styryl)silane (4e)

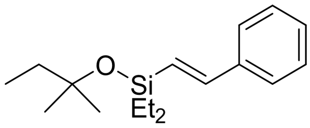

Light yellow oil, yield = 89%, 123 mg, ^{1}H NMR (500 MHz, $CDCl_3$): δ = 7.51 (d, 2H, *J* = 7.5 Hz), 7.41-7.30 (m, 3H), 7.02 (d, 1H, *J* = 19.0 Hz), 6.52 (d, 1H, *J* = 19.5 Hz), 1.57-1.53 (m, 2H), 1.28 (s, 6H), 1.06 (t, 6H, *J* = 7.5 Hz), 0.96 (t, 3H, *J* = 7.5 Hz), 0.80-0.76 (m, 4H). $^{13}C\{^1H\}$ NMR (125 MHz, $CDCl_3$): δ = 144.8, 138.6, 128.7, 128.2, 127.4, 126.6, 74.4, 37.6, 29.6, 9.0, 7.3, 7.2. GC: t_R = 4.23 min (Method B). HRMS (EI): *m/z* calcd for $C_{17}H_{28}NaOSi$ $[M+Na]^+$ 299.1802, found 299.1800.

(E)-tert-butoxydiethyl(styryl)silane (4f)

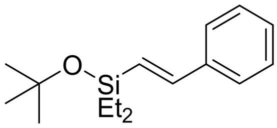

Colorless oil, yield = 85%, 111 mg, ^{1}H NMR (500 MHz, $CDCl_3$): δ = 7.51 (d, 2H, *J* = 7.5 Hz), 7.39 (t, 2H, *J* = 7.0 Hz), 7.32-7.29 (m, 1H), 7.02 (d, 1H, *J* = 19.0 Hz), 6.52 (d, 1H, *J* = 19.5 Hz), 1.33 (s, 9H), 1.06 (t, 6H, *J* = 7.5 Hz), 0.80-0.76 (m, 4H). $^{13}C\{^1H\}$ NMR (125 MHz, $CDCl_3$): δ = 144.9, 138.6, 128.7, 128.2, 127.4, 126.7, 72.3, 32.3, 7.2, 7.1. GC: t_R = 4.11 min (Method B). HRMS (EI): m/z calcd for $C_{19}H_{26}OSiK$ $[M+K]^+$ 301.1384, found 301.1382.

(E)-(cyclohexyloxy)diethyl(styryl)silane (4g)

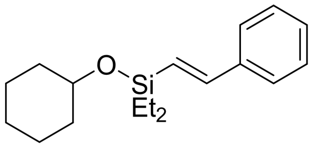

Colorless oil, yield = 72%, 104 mg, ^{1}H NMR (500 MHz, $CDCl_3$): δ = 7.46 (d, 2H, *J* = 7.0 Hz), 7.34 (t, 2H, *J* = 7.5 Hz), 7.28-7.24 (m, 1H), 7.01 (d, 1H, *J* = 19.5 Hz), 6.42 (d, 1H, *J* = 19.5 Hz), 3.67-3.62 (m, 1H), 1.84-1.81 (m, 2H), 1.74-1.71 (m, 2H), 1.35-1.19 (m, 6H), 1.01 (t, 6H, *J* = 8.0 Hz), 0.77-0.72 (m, 4H). $^{13}C\{^1H\}$ NMR (125 MHz, $CDCl_3$): δ = 145.9, 138.4, 128.7, 128.35, 126.7, 125.2, 71.4, 36.2, 25.7, 24.6, 7.0, 5.7. GC: t_R = 5.05 min (Method B). HRMS (EI): *m/z* calcd for $C_{18}H_{28}OSiK$ $[M+K]^+$ 327.1541, found 327.1541.

(E)-diethyl((1-phenylcyclohexyl)oxy)(styryl)silane (4h)

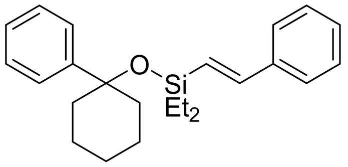

Light yellow oil, yield = 73%, 133 mg, ^{1}H NMR (500 MHz, $CDCl_3$): δ = 7.56-7.54 (m, 2H), 7.39-7.28 (m, 8H), 6.81-6.76 (m, 1H), 6.06-6.01 (m, 1H), 2.13-2.11 (m, 2H), 1.93-1.83 (m, 4H), 1.72-1.70 (m, 1H), 1.62-1.60 (m, 2H), 1.34-1.32 (m, 1H), 1.00-0.96 (m, 6H), 0.64-0.58 (m, 4H). ^{13}C{^{1}H} NMR (125 MHz, $CDCl_3$): δ = 148.4, 144.3, 138.6, 128.6, 128.1, 127.1, 126.8, 126.6, 126.2, 75.5, 39.2, 26.0, 22.7, 7.1, 6.6. GC: t_R = 10.41 min (Method A). HRMS (EI): *m/z* calcd for $C_{24}H_{32}NaOSi$ $[M+Na]^+$ 387.2115, found 387.2115.

(E)-(benzhydryloxy)diethyl(styryl)silane (4i)

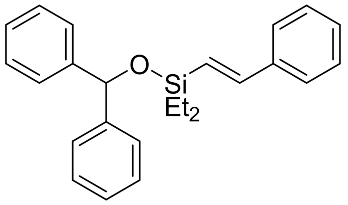

Light yellow oil, yield = 77%, 143 mg, ^{1}H NMR (500 MHz, $CDCl_3$): δ = 7.49-7.32 (m, 15H), 7.04-7.00 (m, 1H), 6.42-6.38 (m, 1H), 5.95 (s, 1H), 1.08-1.01 (m, 6H), 0.83-0.81 (m, 4H). ^{13}C{^{1}H} NMR (125 MHz, $CDCl_3$): δ = 146.5, 145.1, 138.2, 128.7, 128.42, 128.39, 128.36, 128.3, 127.9, 127.2, 126.7, 126.6, 126.5, 124.2, 76.8, 6.9, 5.7. GC: t_R = 11.57 min (Method A). HRMS (EI): *m/z* calcd for $C_{25}H_{28}NaOSi$ $[M+Na]^+$ 395.1802, found 395.1801.

(E)-2-(2-((diethyl(styryl)silyl)oxy)ethyl)pyridine (4j)

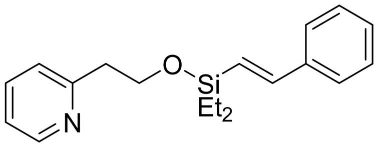

Colorless oil, yield = 77%, 120 mg, ^{1}H NMR (500 MHz, $CDCl_3$): δ = 8.54-8.53 (m, 1H), 7.64-7.61 (m, 1H), 7.43 (d, 2H, *J* = 7.0 Hz), 7.35 (d, 2H, *J* = 7.0 Hz), 7.29-7.26 (m, 2H), 7.16-7.13 (m, 1H), 6.93 (d, 1H, *J* = 19.5 Hz), 6.29 (d, 1H, *J* = 19.5 Hz), 4.07 (t, 2H, *J* = 6.5 Hz), 308 (t, 2H, *J* = 6.5 Hz), 0.97 (t, 6H, *J* = 8.0 Hz), 0.73-0.68 (m, 4H). ^{13}C{^{1}H} NMR (125 MHz, $CDCl_3$): δ = 159.4, 149.0, 146.3, 138.2, 136.6, 128.7, 128.4, 126.7, 124.4, 124.0, 121.6, 62.8, 41.6, 6.8, 5.1. GC: t_R = 5.08 min (Method B). HRMS (EI): *m/z* calcd for $C_{19}H_{26}NOSi$ $[M+H]^+$ 312.1778, found 312.1778.

(E)-diethyl(1-(4-methoxyphenyl)ethoxy)(styryl)silane (5a)

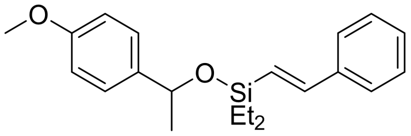

Light yellow oil, yield = 89%, 151 mg, ^{1}H NMR (300 MHz, $CDCl_3$): δ = 7.45-7.30 (m, 7H) 7.01-6.88 (m, 3H), 6.36 (d, 1H, J = 19.2 Hz), 5.02-4.86 (m, 1H), 3.83 (s, 3H), 1.48 (d, 3H, J = 6.0 Hz), 1.06-0.96 (m, 6H), 0.81-0.70 (m, 4H). ^{13}C{^{1}H} NMR (75 MHz, $CDCl_3$): δ = 158.7, 146.1, 139.0, 138.3, 128.7, 128.4, 126.8, 126.7, 124.7, 113.7, 70.8, 55.4, 27.2, 6.9, 6.8, 5.7, 5.6. GC: t_R = 9.67 min (Method A). HRMS (EI): *m/z* calcd for $C_{21}H_{28}NaO_2Si$ $[M+Na]^+$ 363.1751, found 363.1748.

(E)-diethyl(1-(4-methoxyphenyl)ethoxy)(4-methoxystyryl)silane (5b)

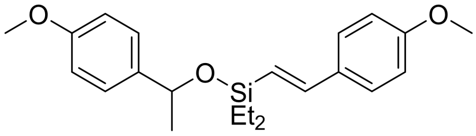

Light yellow oil, yield = 87%, 161 mg, ^{1}H NMR (500 MHz, $CDCl_3$): δ = 7.39-7.31 (m, 4H), 6.94-6.88 (m, 5H), 6.19 (d, 1H, J = 19.5 Hz), 4.95-4.91 (m, 1H), 3.85 (s, 3H), 3.84 (s, 3H), 1.49 (d, 3H, J = 6.5 Hz), 1.05-0.96 (m, 6H), 0.79-0.67 (m, 4H). ^{13}C{^{1}H} NMR (125 MHz, $CDCl_3$): δ = 159.9, 158.6, 145.6, 139.1, 131.3, 128.0, 126.8, 121.7, 114.0, 113.6, 70.6, 55.5, 55.4, 27.2, 7.0, 6.9, 5.7, 5.6. GC: t_R = 10.04 min (Method A). HRMS (EI): *m/z* calcd for $C_{22}H_{31}O_3Si$ $[M+H]^+$ 371.2037, found 371.2036.

(E)-diethyl(1-(4-methoxyphenyl)ethoxy)(4-methylstyryl)silane (5c)

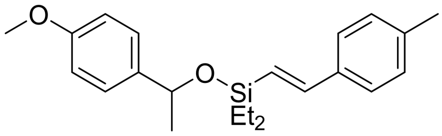

Light yellow oil, yield = 74%, 131 mg, ^{1}H NMR (500 MHz, $CDCl_3$): δ = 7.36-7.28 (m, 4H), 7.18 (d, 2H, J = 7.5 Hz), 6.98-6.89 (m, 3H), 6.31 (d, 1H, J = 19.5 Hz), 4.96-4.92 (m, 1H), 3.84 (s, 3H), 2.39 (s, 3H), 1.49 (d, 3H, J = 6.5 Hz), 1.05-0.97 (m, 6H), 0.80-0.68 (m, 4H). ^{13}C{^{1}H} NMR (125 MHz, $CDCl_3$): δ = 158.6, 146.0, 139.0, 138.3, 135.6, 129.4, 126.8, 126.6, 123.2, 113.6, 70.7, 55.4, 27.2, 21.4, 7.0, 6.9, 5.7, 5.6. GC: t_R = 9.61 min (Method A). HRMS (EI): *m/z* calcd for $C_{22}H_{30}NaO_2Si$ $[M+Na]^+$ 377.1907, found 377.1904.

(E)-diethyl(1-(4-methoxyphenyl)ethoxy)(3-methylstyryl)silane (5d)

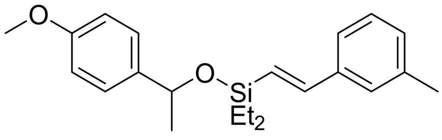

Light yellow oil, yield = 68%, 120 mg, ^{1}H NMR (500 MHz, $CDCl_3$): δ = 7.36-7.34 (m, 2H), 7.30-7.28 (m, 2H), 7.16-7.15 (m, 1H), 7.00-6.92 (m, 3H), 6.38 (d, 1H, J = 19.5 Hz), 4.98-4.95 (m, 1H), 3.86 (s, 3H), 2.43 (s, 3H), 1.52 (d, 3H, J = 6.5 Hz), 1.09-1.00 (m, 6H), 0.83-0.70 (m, 4H). $^{13}C\{^{1}H\}$ NMR (125 MHz, $CDCl_3$): δ = 158.6, 146.2, 139.0, 138.2, 129.2, 128.5, 127.4, 126.8, 126.6, 124.3, 123.9, 113.6, 70.7, 55.3, 27.2, 21.5, 7.0, 6.9, 5.6, 5.5. GC: t_R = 9.59 min (Method A). HRMS (EI): *m/z* calcd for $C_{22}H_{30}NaO_2Si$ $[M+Na]^+$ 377.1907, found 377.1903.

(E)-(4-(tert-butyl)styryl)diethyl(1-(4-methoxyphenyl)ethoxy)silane (5e)

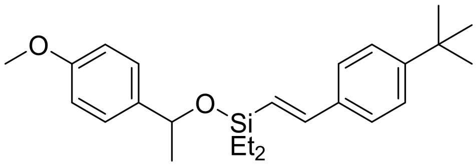

Light yellow oil, yield = 74%, 147 mg, ^{1}H NMR (500 MHz, $CDCl_3$): δ = 7.39-7.29 (m, 6H), 6.96-6.88 (m, 3H), 6.30 (d, 1H, J = 19.5 Hz), 4.93-4.89 (m, 1H), 3.83 (s, 3H), 1.47 (d, 3H, J = 6.0 Hz), 1.35 (s, 9H), 1.03-0.94 (m, 6H), 0.78-0.66 (m, 4H). $^{13}C\{^{1}H\}$ NMR (125 MHz, $CDCl_3$): δ = 158.6, 151.6, 145.9, 139.0, 135.6, 126.8, 126.4, 125.6, 123.6, 113.6, 70.7, 55.4, 34.8, 31.5, 27.2, 7.0, 6.9, 5.7, 5.6. GC: t_R = 10.29 min (Method A). HRMS (EI): *m/z* calcd for $C_{25}H_{36}NaO_2Si$ $[M+Na]^+$ 419.2377, found 419.2373.

(E)-diethyl(4-fluorostyryl)(1-(4-methoxyphenyl)ethoxy)silane (5f)

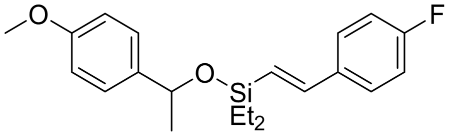

Light yellow oil, yield = 63%, 113 mg, ^{1}H NMR (500 MHz, $CDCl_3$): δ = 7.39-7.37 (m, 2H), 7.31-7.28 (m, 2H), 7.06-7.02 (m, 2H), 6.92-6.87 (m, 3H), 6.23 (d, 1H, J = 19.0 Hz), 4.93-4.89 (m, 1H), 3.82 (s, 3H), 1.47 (d, 3H, J = 6.0 Hz), 1.02-0.95 (m, 6H), 0.77-0.67 (m, 4H). $^{13}C\{^{1}H\}$ NMR (125 MHz, $CDCl_3$): δ = 161.9 (J_{CF} = 246.3 Hz), 158.7, 144.8, 138.9, 134.5 (J_{CF} = 3.4 Hz), 128.3 (J_{CF} = 8.0 Hz), 126.8, 124.4 (J_{CF} = 2.3 Hz), 115.6 (J_{CF} = 21.1 Hz), 113.6, 70.8, 55.4, 27.2, 7.0, 6.9, 5.6, 5.5. ^{19}F NMR (470 MHz, $CDCl_3$): δ = 113.6 Hz. GC: t_R = 8.76 min (Method A). HRMS (EI): *m/z* calcd for $C_{21}H_{27}FNaO_2Si$ $[M+Na]^+$ 381.1657, found 381.1651.

(E)-(4-chlorostyryl)diethyl(1-(4-methoxyphenyl)ethoxy)silane (5g)

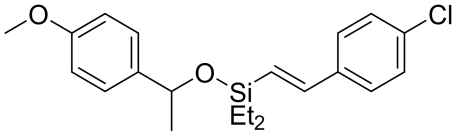

Light yellow oil, yield = 78%, 146 mg, ^{1}H NMR (500 MHz, $CDCl_3$): δ = 7.35-7.28 (m, 6H), 6.90-6.86 (m, 3H), 6.30 (d, 1H, J = 19.5 Hz), 4.93-4.89 (m, 1H), 3.83 (s, 3H), 1.47 (d, 3H, J = 6.5 Hz), 1.03-0.95 (m, 6H), 0.78-0.66 (m, 4H). $^{13}C\{^{1}H\}$ NMR (125

MHz, $CDCl_3$): δ = 158.7, 144.7, 138.9, 136.7, 134.0, 128.8, 127.9, 126.8, 125.6, 113.6, 70.8, 55.4, 27.2, 6.9, 6.8, 5.6, 5.5. GC: t_R = 9.63 min (Method A). HRMS (EI): *m/z* calcd for $C_{21}H_{27}ClNaO_2Si$ $[M+Na]^+$ 397.1361, found 397.1360.

(E)-(4-bromostyryl)diethyl(1-(4-methoxyphenyl)ethoxy)silane (5h)

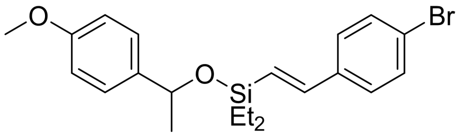

Light yellow oil, yield = 67%, 140 mg, ^1H NMR (500 MHz, $CDCl_3$): δ = 7.48 (d, 2H, *J* = 8.5 Hz) 7.32-7.27 (m, 4H), 6.90-6.86 (m, 3H), 6.33 (d, 1H, *J* = 19.5 Hz), 4.94-4.90 (m, 1H), 3.83 (s, 3H), 1.49 (d, 3H, *J* = 6.0 Hz), 1.07-0.97 (m, 6H), 0.81-0.67 (m, 4H). $^{13}C\{^1H\}$ NMR (125 MHz, $CDCl_3$): δ = 158.7, 144.7, 138.8, 137.2, 131.7, 128.2, 126.8, 125.8, 122.2, 113.6, 70.8, 55.4, 27.2, 6.9, 6.8, 5.6, 5.5. GC: t_R = 9.95 min (Method A). HRMS (EI): *m/z* calcd for $C_{21}H_{27}BrNaO_2Si$ $[M+Na]^+$ 441.0856, found 441.0853.

(E)-4-(2-(diethyl(1-(4-methoxyphenyl)ethoxy)silyl)vinyl)pyridine (5i)

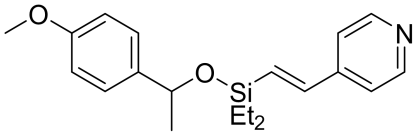

Light yellow oil, yield = 73%, 124 mg, ^1H NMR (500 MHz, $CDCl_3$): δ = 8.57 (d, 2H, *J* = 5.5 Hz), 7.29-7.23 (m, 4H), 6.88-6.82 (m, 3H), 6.55 (d, 1H, *J* = 19.5 Hz), 4.91-4.87 (m, 1H), 3.81 (s, 3H), 1.47 (d, 3H, *J* = 6.0 Hz), 1.01-0.95 (m, 6H), 0.78-0.68 (m, 4H). $^{13}C\{^1H\}$ NMR (125 MHz, $CDCl_3$): δ = 158.8, 150.2, 145.2, 143.3, 138.7, 131.2, 126.8, 121.1, 113.7, 71.0, 55.4, 27.2, 6.9, 6.8, 5.5, 5.4. GC: t_R = 9.73 min (Method A). HRMS (EI): *m/z* calcd for $C_{20}H_{28}NO_2Si$ $[M+H]^+$ 342.1884, found 342.1882.

2,2-diethyl-2H-naphtho[1,8-cd][1,2]oxasilole (7a)

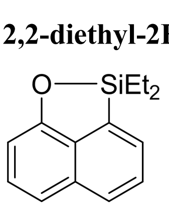

Light yellow oil, yield = 88%, 115 mg, ^1H NMR (500 MHz, $CDCl_3$): δ = 7.77 (d, 1H, *J* = 8.0 Hz), 7.60 (d, 1H, *J* = 6.5 Hz), 7.48-7.46 (m, 1H), 7.32-7.26 (m, 2H), 6.83-6.81 (m, 1H), 0.97-0.91 (m, 10H). $^{13}C\{^1H\}$ NMR (125 MHz, $CDCl_3$): δ = 159.1, 134.3, 132.4, 132.1, 129.3, 128.1, 128.0, 127.5, 118.0, 106.9, 6.4, 6.2. GC: t_R = 4.37 min (Method B). HRMS (EI): *m/z* calcd for $C_{14}H_{17}OSi$ $[M+H]^+$ 229.1043, found 229.1042.

2,2-diethyl-6-methoxy-2H-naphtho[1,8-cd][1,2]oxasilole (7b)

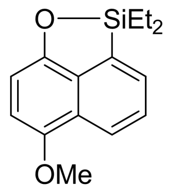

Light yellow oil, yield = 59%, 76 mg, ^{1}H NMR (500 MHz, $CDCl_3$): δ = 8.23-8.21 (m, 1H), 7.77-7.76 (m, 1H), 7.63-7.60 (m, 1H), 6.84 (d, 1H, J = 8.0 Hz), 6.72 (d, 1H, J = 8.5 Hz), 3.99 (s, 3H), 1.09-1.03 (m, 10H). ^{13}C{^{1}H} NMR (125 MHz, $CDCl_3$): δ = 152.7, 148.8, 134.9, 131.8, 129.9, 126.9, 124.4, 123.2, 105.4, 104.9, 55.9, 6.5, 6.2. GC: t_R = 8.25 min (Method A).HRMS (EI): m/z calcd for $C_{15}H_{19}O_2Si$ $[M+H]^+$ 259.1149, found 259.1149.

6-chloro-2,2-diethyl-2H-naphtho[1,8-cd][1,2]oxasilole (7c)

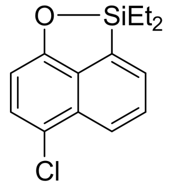

Light yellow oil, yield = 71%, 93 mg, ^{1}H NMR (500 MHz, $CDCl_3$): δ = 8.18-8.17 (m, 1H), 7.78-7.68 (m, 2H), 7.45 (d, 1H, J = 8.0 Hz), 6.84 (d, 1H, J = 8.0 Hz), 1.08-1.00 (m, 10H). ^{13}C{^{1}H} NMR (125 MHz, $CDCl_3$): δ = 158.2, 135.1, 132.6, 130.1, 129.8, 128.4, 127.6, 125.3, 121.2, 107.2, 6.3, 6.2. GC: t_R = 7.29 min (Method A). HRMS (EI): m/z calcd for $C_{14}H_{16}ClOSi$ $[M+H]^+$ 263.0653, found 263.0648.

6-bromo-2,2-diethyl-2H-naphtho[1,8-cd][1,2]oxasilole (7d)

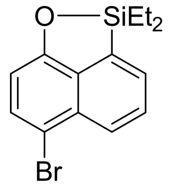

Light yellow oil, yield = 82%, 126 mg, ^{1}H NMR (500 MHz, $CDCl_3$): δ = 8.14-8.12 (m, 1H), 7.77-7.65 (m, 3H), 6.83 (d, 2H, J = 8.0 Hz), 1.09-1.01 (m, 10H). ^{13}C{^{1}H} NMR (125 MHz, $CDCl_3$): δ = 158.9, 135.3, 132.7, 131.2, 131.1, 130.2, 128.7, 127.7, 110.7, 108.0, 6.3, 6.2. GC: t_R = 7.79 min (Method A). HRMS (EI): m/z calcd for $C_{14}H_{16}BrOSi$ $[M+H]^+$ 307.1498, found 307.1502.

4,4-diethyl-4H-pyreno[10,1-cd][1,2]oxasilole (7e)

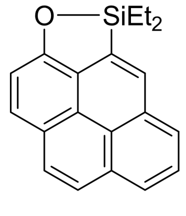

Light yellow oil, yield = 70%, 106 mg, ^{1}H NMR (500 MHz, $CDCl_3$): δ = 8.33 (s, 1H), 8.21-8.12 (m, 3H), 8.04-8.02 (m, 2H), 7.92 (d, 1H, *J* = 9.0 Hz), 7.63 (d, 1H, *J* = 8.5 Hz), 1.21-1.13 (m, 10H). $^{13}C\{^1H\}$ NMR (125 MHz, $CDCl_3$): δ = 157.3, 132.5, 131.8, 131.1, 131.0, 127.5, 127.1, 126.5, 126.2, 125.6, 124.8, 124.6, 124.4, 123.5, 111.4, 6.5, 6.3. GC: t_R = 14.09 min (Method A). HRMS (EI): *m/z* calcd for $C_{20}H_{19}OSi$ $[M+H]^+$ 303.1199, found 303.1201.

3,3-diethyl-1,3-dihydronaphtho[2,1-c][1,2]oxasilole (9a)

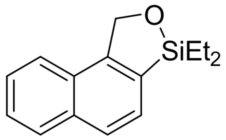

Light yellow oil, yield = 88%, 106 mg, ^{1}H NMR (500 MHz, $CDCl_3$): δ = 7.95-7.93 (m, 1H), 7.82 (d, 1H, *J* = 8.0 Hz), 7.74-7.72 (m, 1H), 7.64 (d, 1H, *J* = 8.0 Hz), 7.58-7.56 (m, 2H), 1.02-0.90 (m, 10H). $^{13}C\{^1H\}$ NMR (125 MHz, $CDCl_3$): δ = 148.1, 134.2, 130.6, 128.8, 128.1, 127.5, 127.4, 126.7, 126.5, 123.2, 7.8, 7.3, 6.7. GC: t_R = 5.68 min (Method A). HRMS (EI): *m/z* calcd for $C_{15}H_{19}OSi$ $[M+H]^+$ 243.1199, found 243.1203.

(E)-diethyl(1-phenylpropoxy)(styryl)silane (4a)

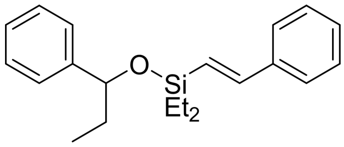

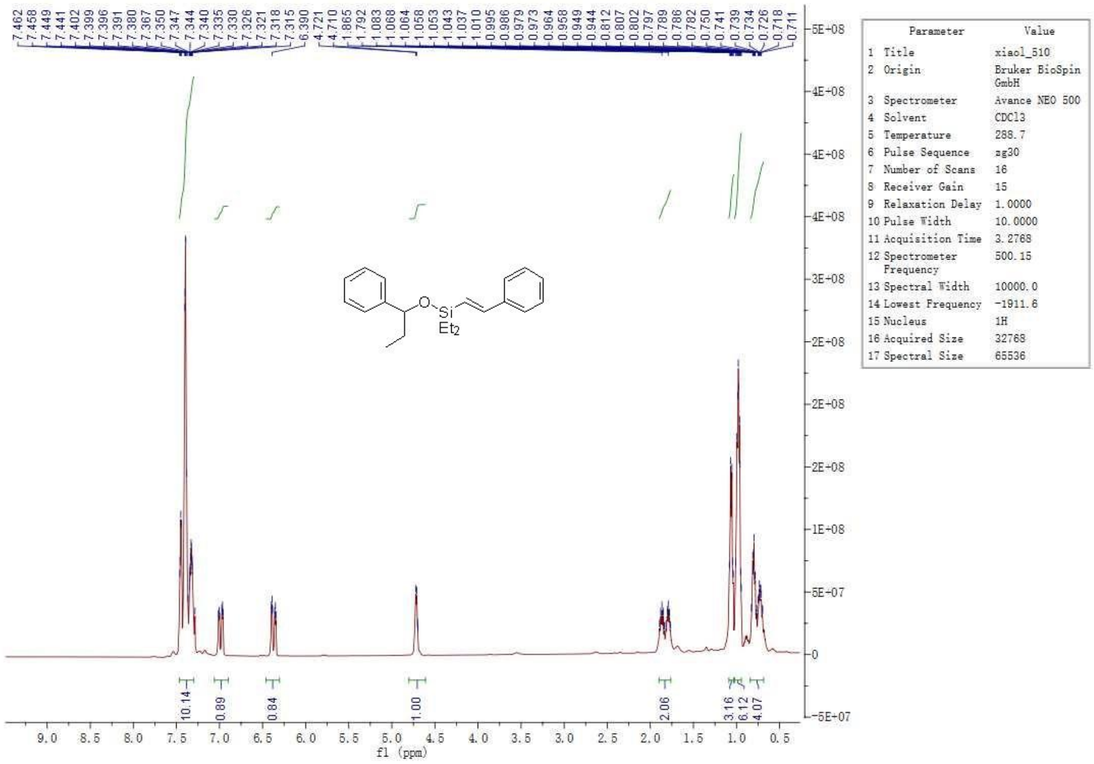

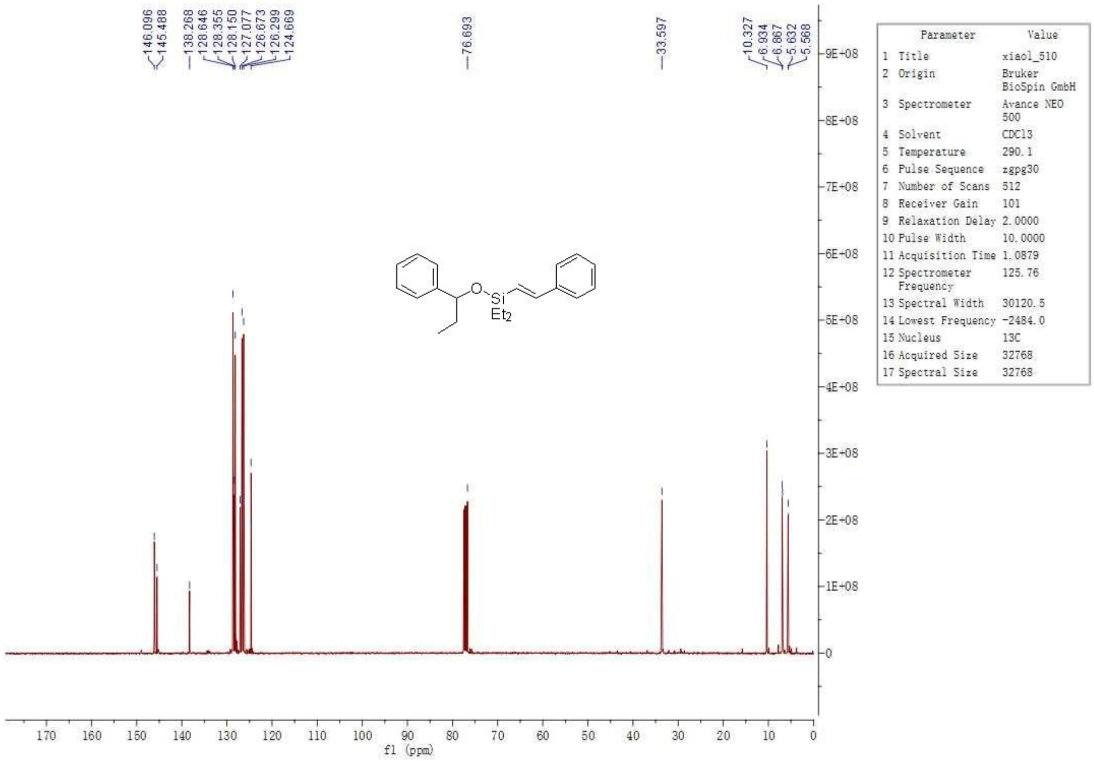

(E)-diethyl(styryl)(1-(p-tolyl)ethoxy)silane (4b)

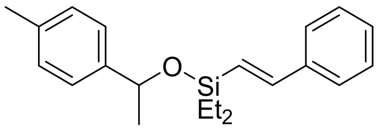

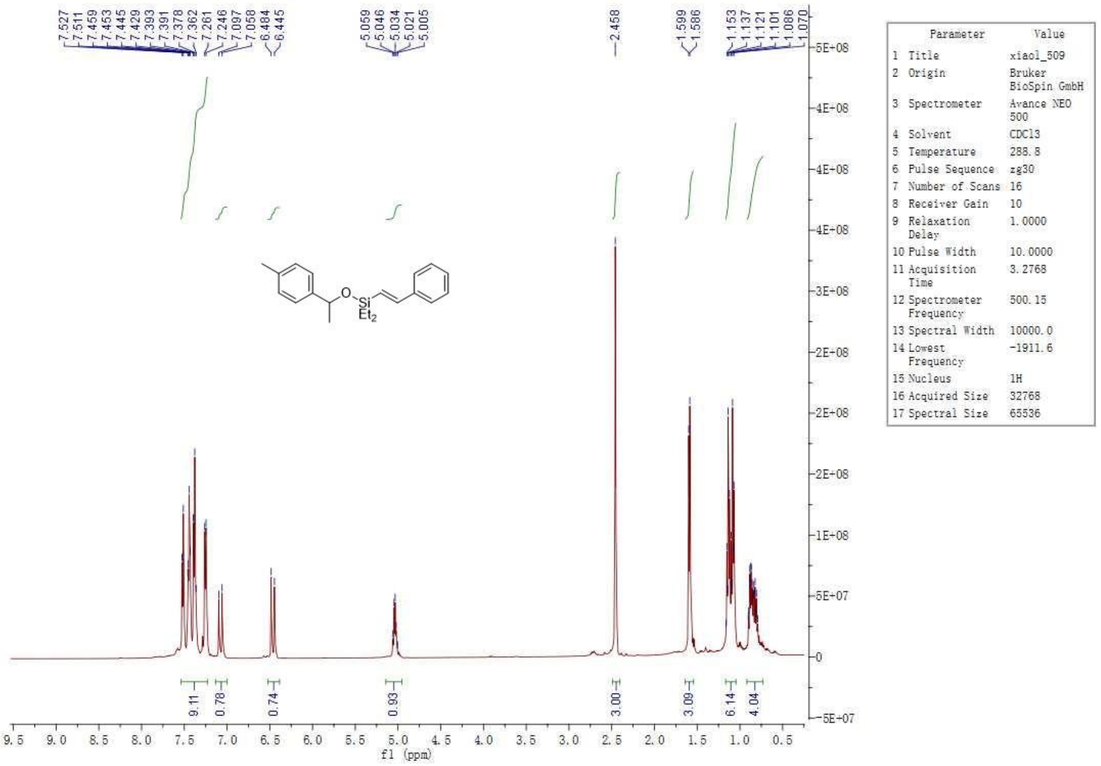

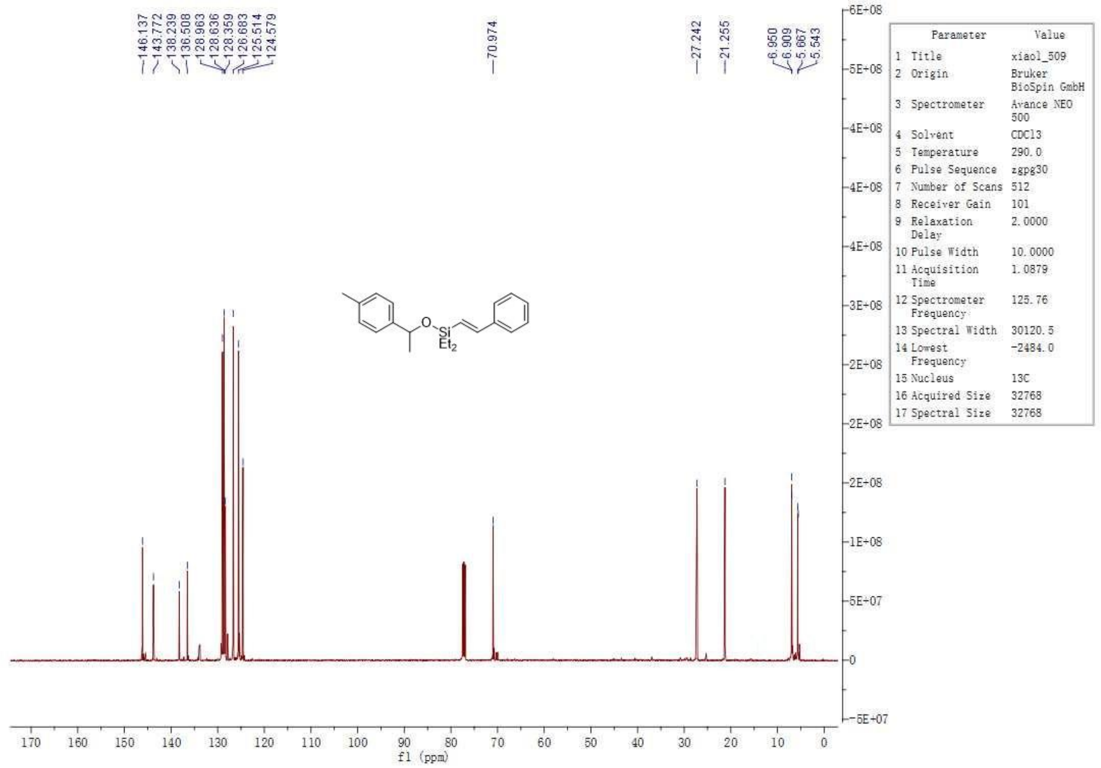

(E)-(1-(4-chlorophenyl)ethoxy)diethyl(styryl)silane (4c)

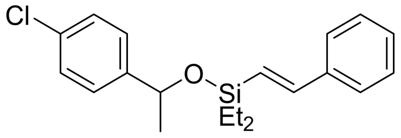

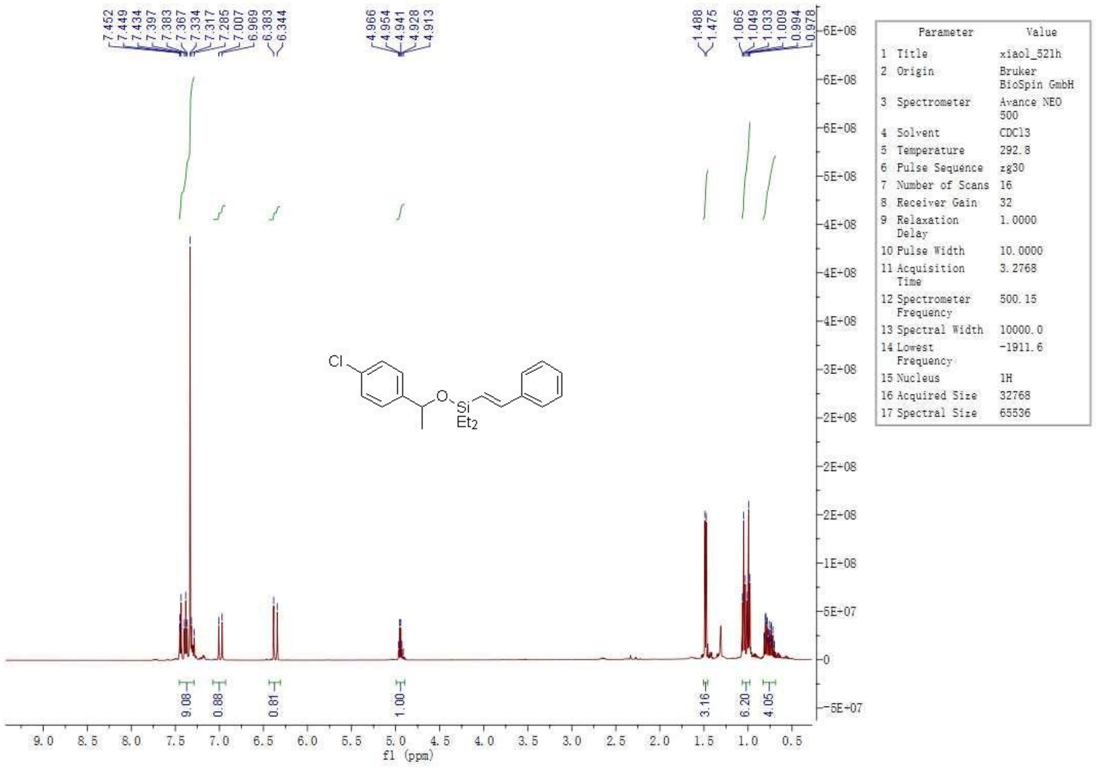

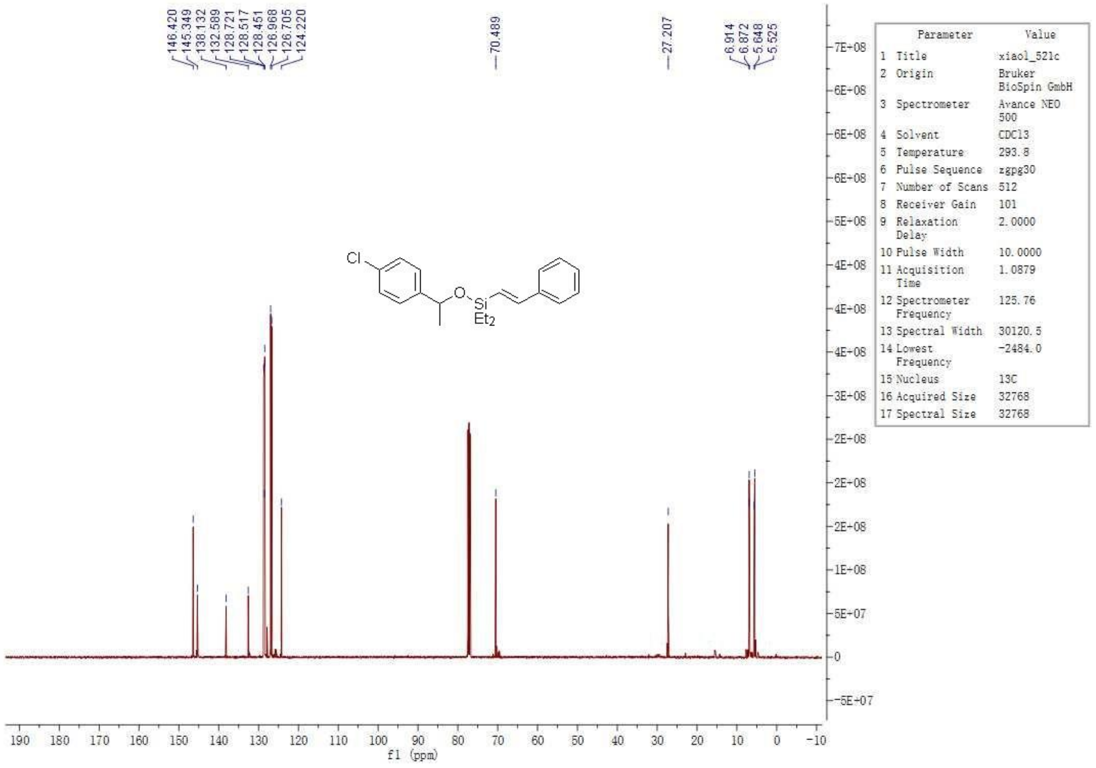

(E)-diethyl((2-phenylpropan-2-yl)oxy)(styryl)silane (4d)

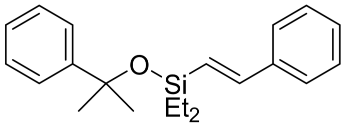

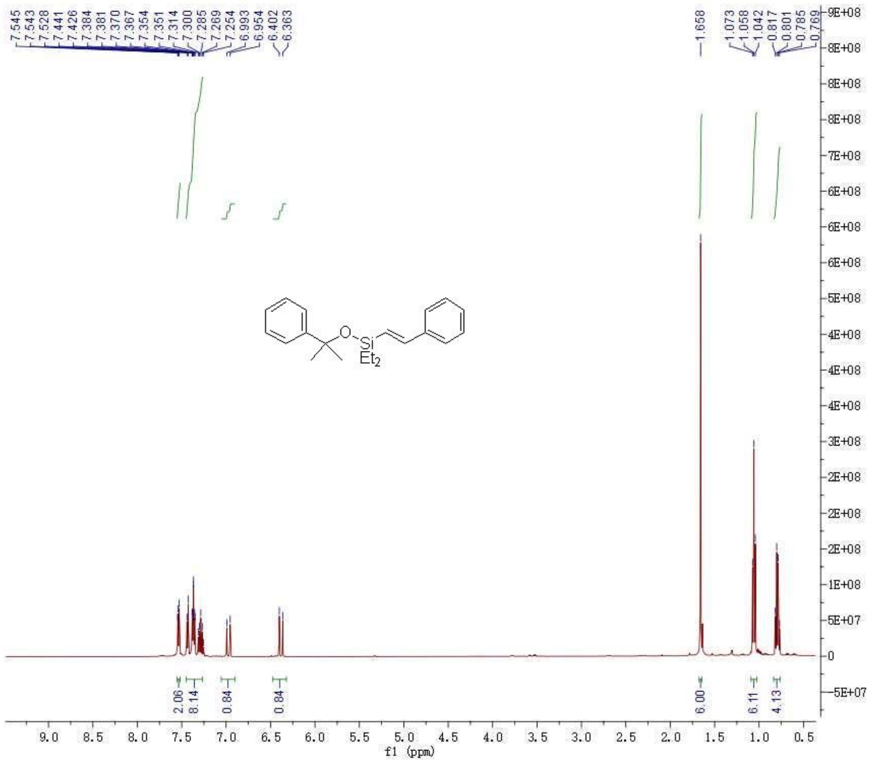

Parameter	Value
1 Title	xiaol-524h
2 Origin	Bruker BioSpin GmbH
3 Spectrometer	Avance NEO 500
4 Solvent	CDCl3
5 Temperature	291.8
6 Pulse Sequence	zg30
7 Number of Scans	16
8 Receiver Gain	32
9 Relaxation Delay	1.0000
10 Pulse Width	10.0000
11 Acquisition Time	3.2768
12 Spectrometer Frequency	500.15
13 Spectral Width	10000.0
14 Lowest Frequency	-1911.6
15 Nucleus	1H
16 Acquired Size	32768
17 Spectral Size	65536

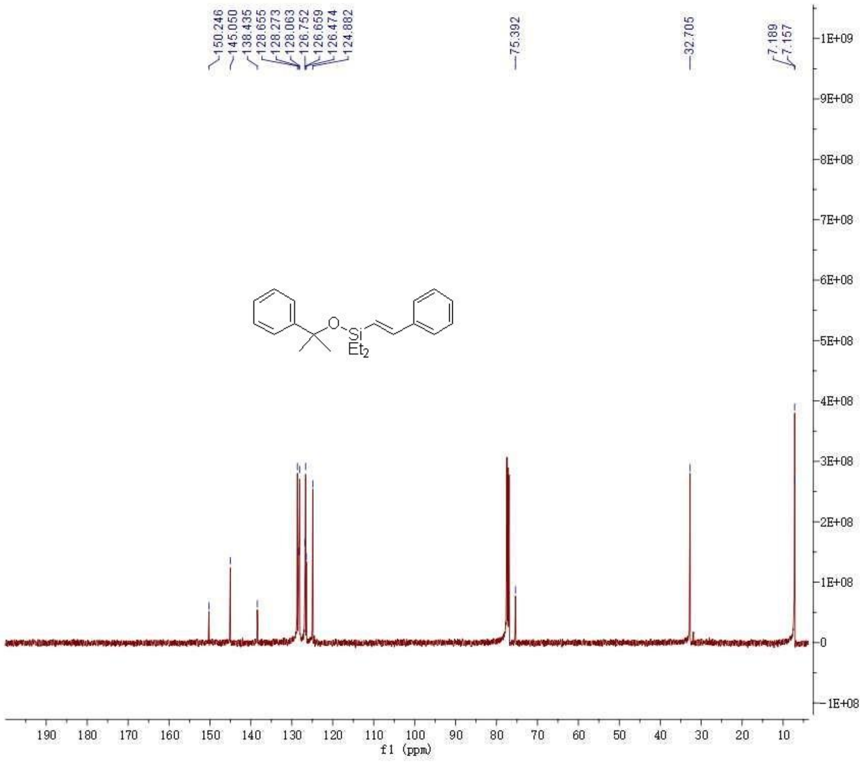

Parameter	Value
1 Title	xiaol_524c
2 Origin	Bruker BioSpin GmbH
3 Spectrometer	Avance NEO 500
4 Solvent	CDCl3
5 Temperature	290.3
6 Pulse Sequence	zgpg30
7 Number of Scans	512
8 Receiver Gain	101
9 Relaxation Delay	2.0000
10 Pulse Width	10.0000
11 Acquisition Time	1.0879
12 Spectrometer Frequency	125.76
13 Spectral Width	30120.5
14 Lowest Frequency	-2484.0
15 Nucleus	13C
16 Acquired Size	32768
17 Spectral Size	32768

(E)-diethyl(tert-pentyloxy)(styryl)silane (4e)

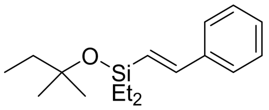

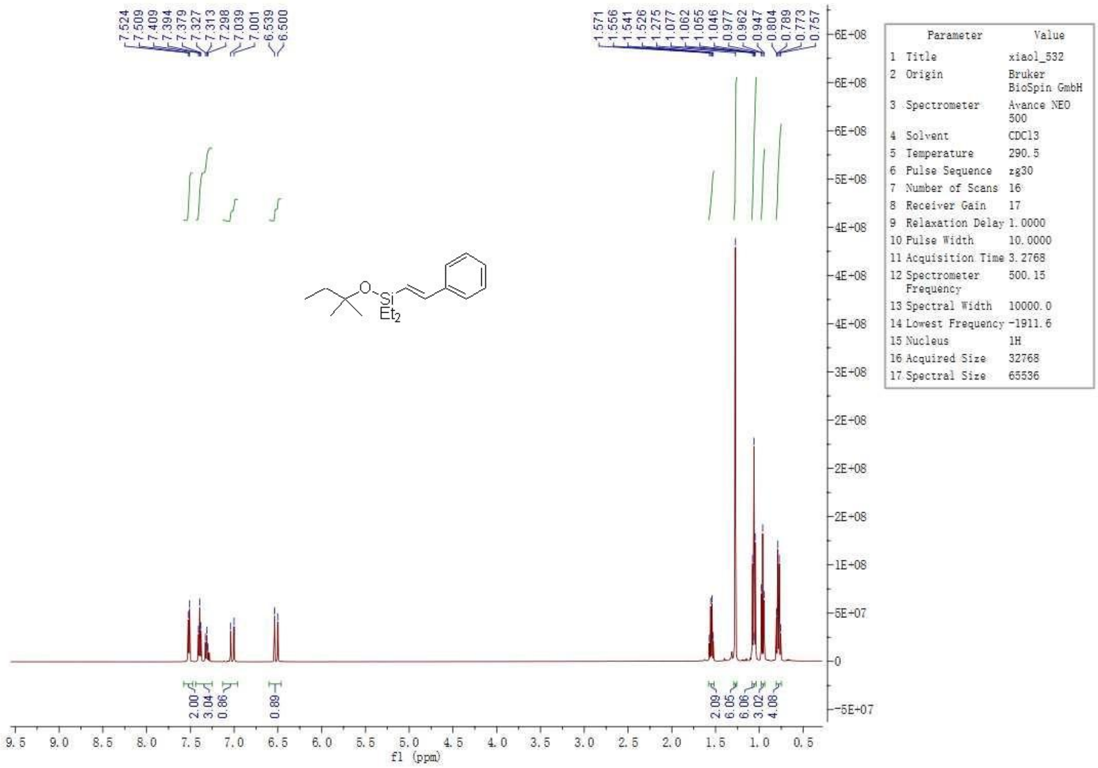

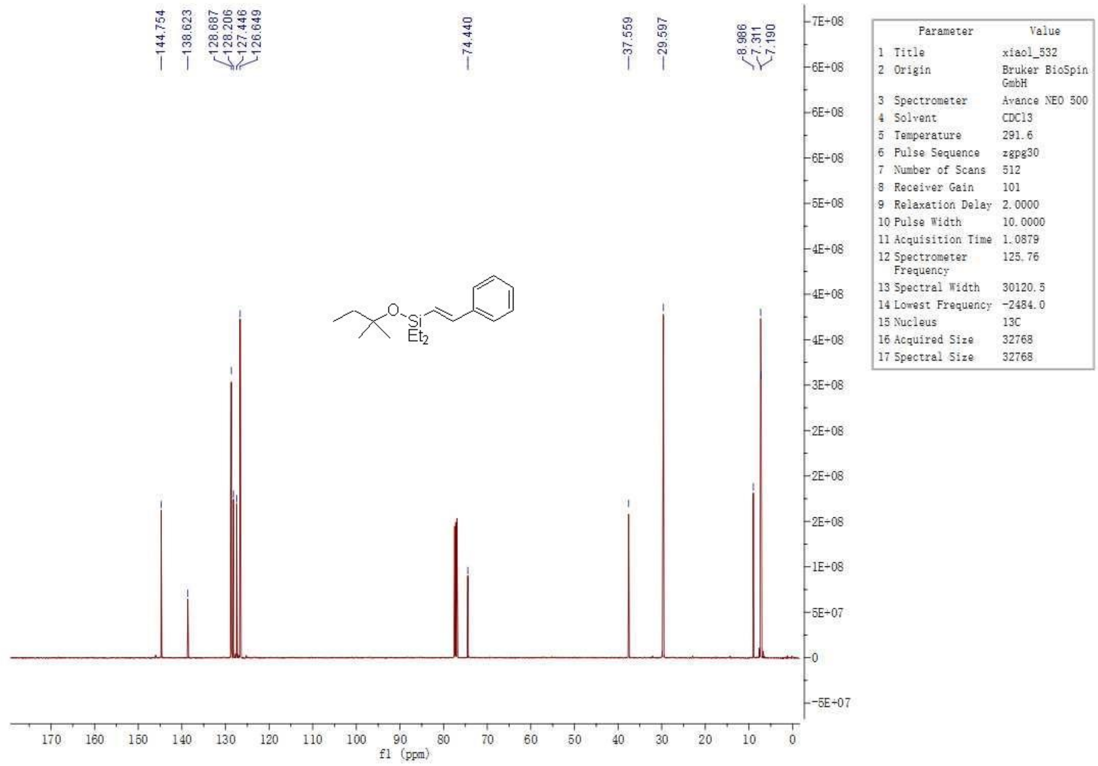

(E)-tert-butoxydiethyl(styryl)silane (4f)

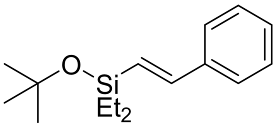

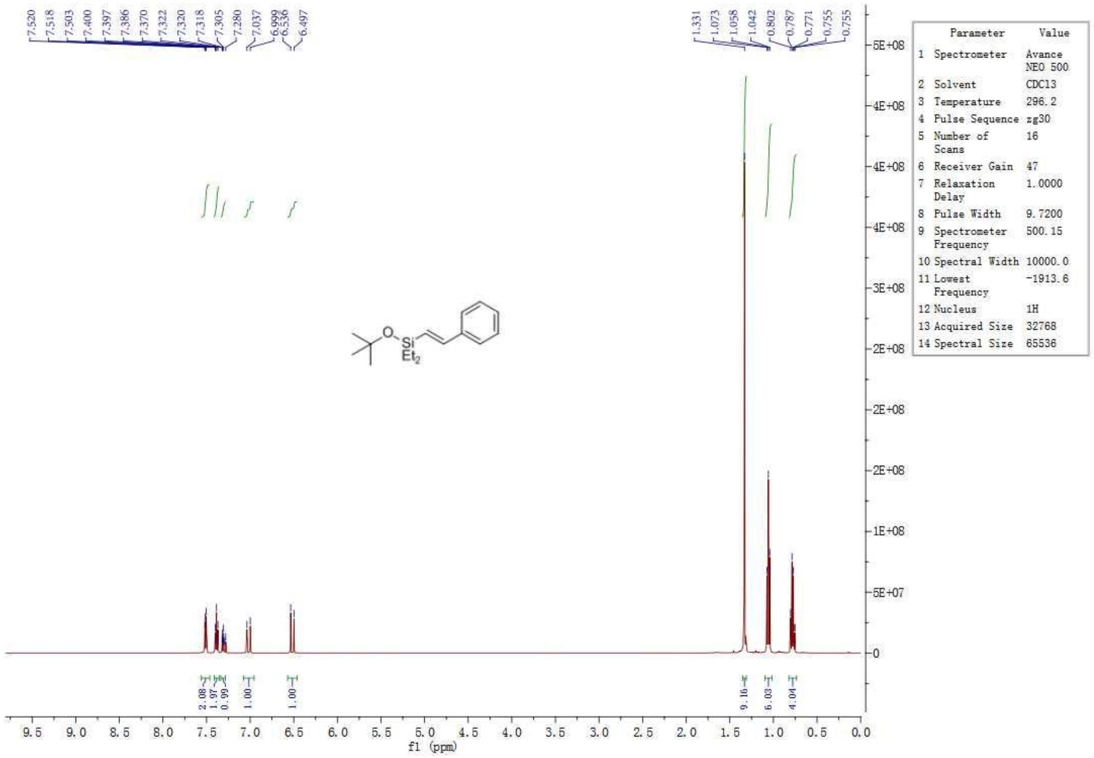

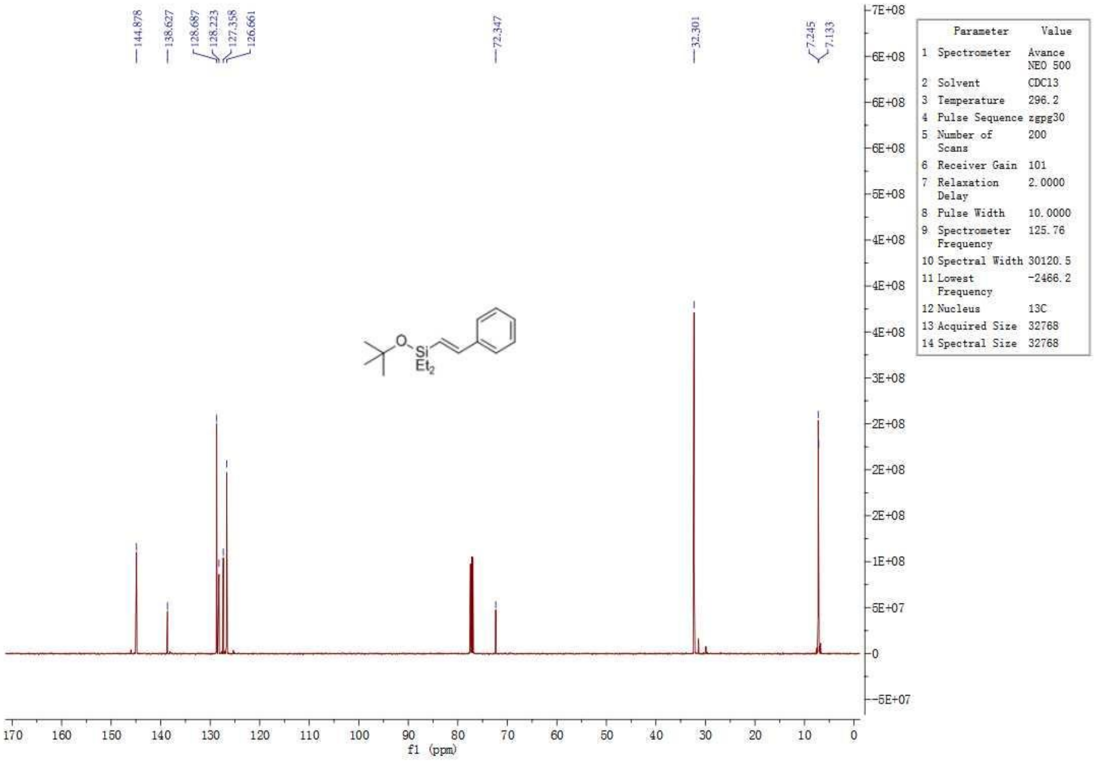

(E)-(cyclohexyloxy)diethyl(styryl)silane (4g)

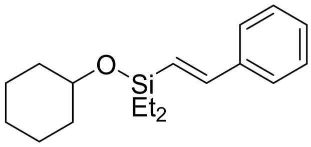

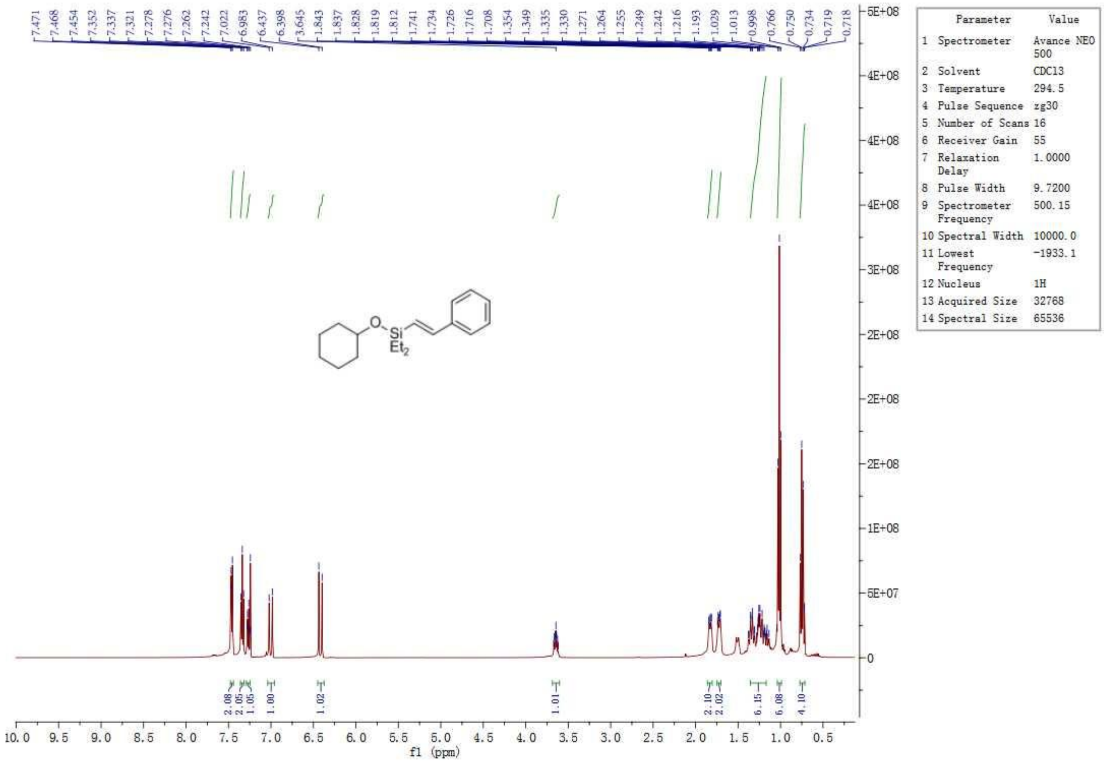

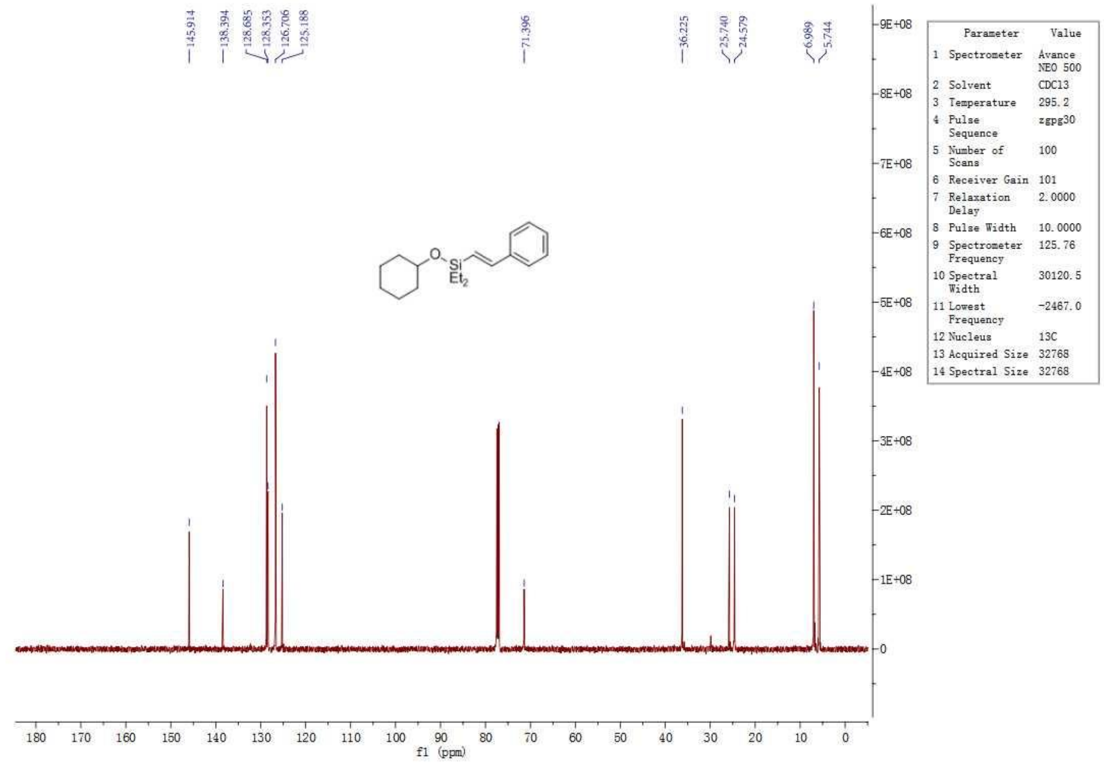

(E)-diethyl((1-phenylcyclohexyl)oxy)(styryl)silane (4h)

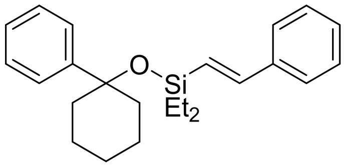

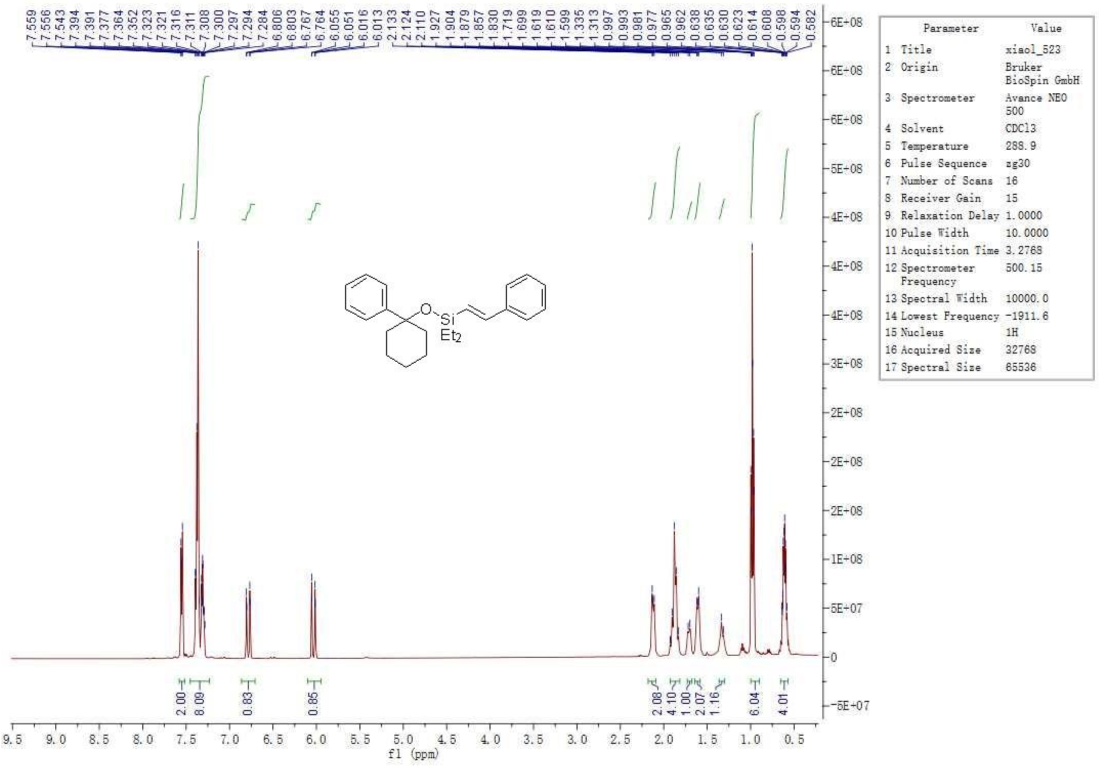

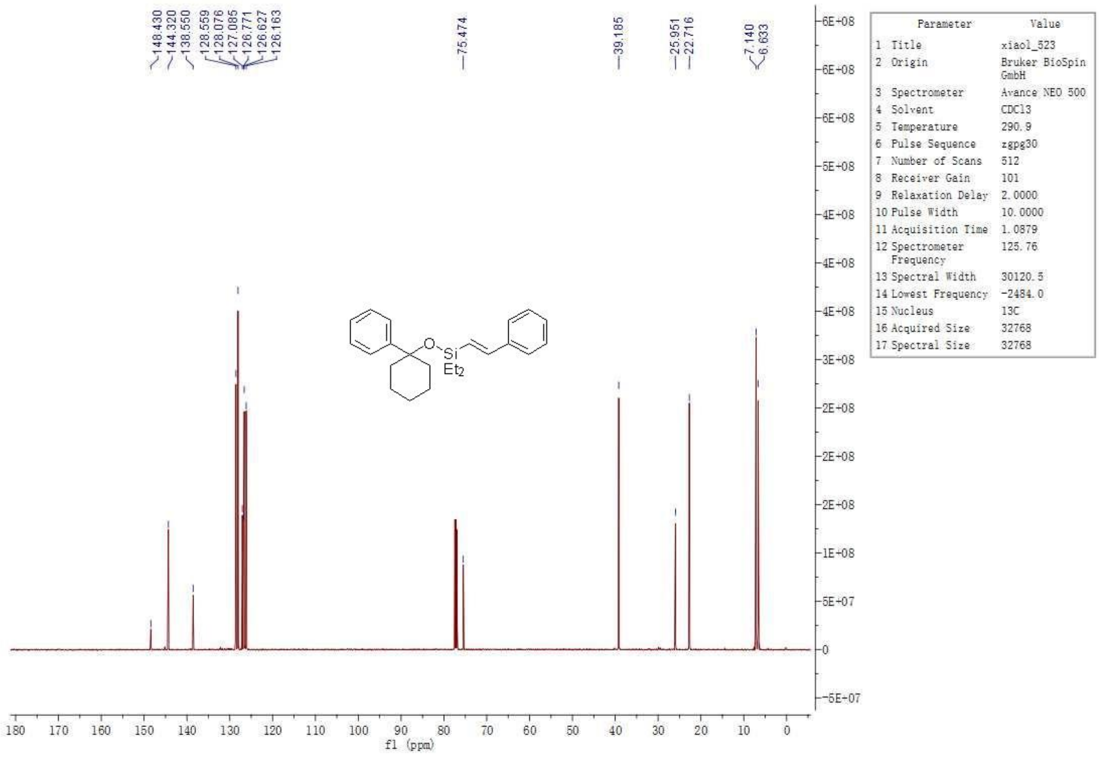

(E)-(benzhydryloxy)diethyl(styryl)silane (4i)

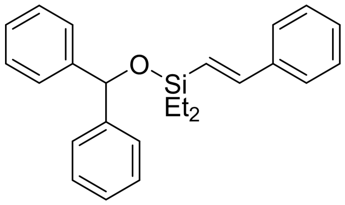

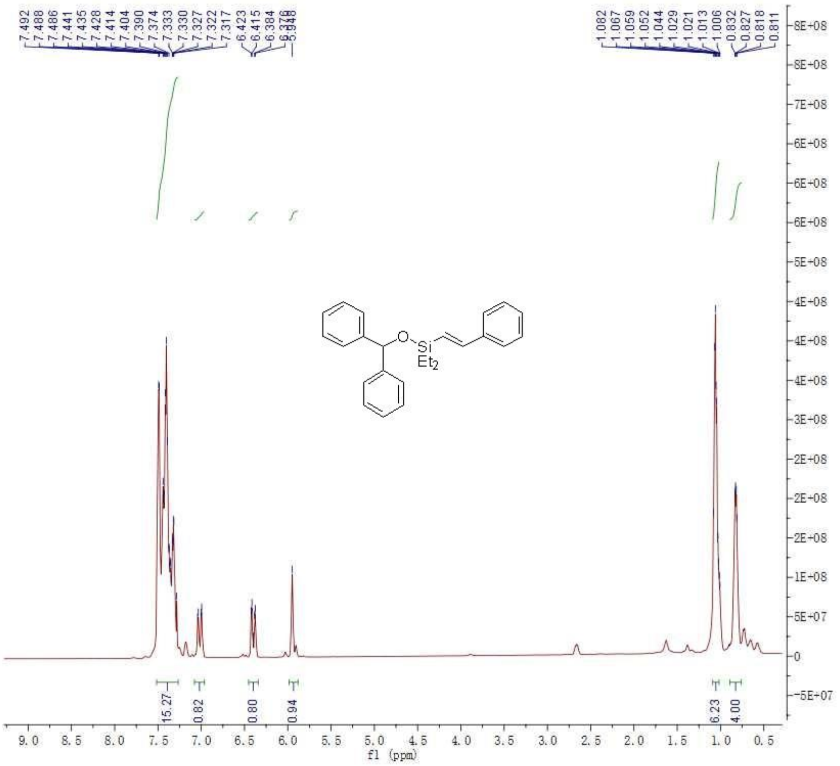

Parameter	Value
1 Title	xiaol_522
2 Origin	Bruker BioSpin GmbH
3 Spectrometer	Avance NEO 500
4 Solvent	CDCl3
5 Temperature	291.2
6 Pulse Sequence	zg30
7 Number of Scans	16
8 Receiver Gain	15
9 Relaxation Delay	1.0000
10 Pulse Width	10.0000
11 Acquisition Time	3.2768
12 Spectrometer Frequency	500.15
13 Spectral Width	10000.0
14 Lowest Frequency	-1911.6
15 Nucleus	1H
16 Acquired Size	32768
17 Spectral Size	65536

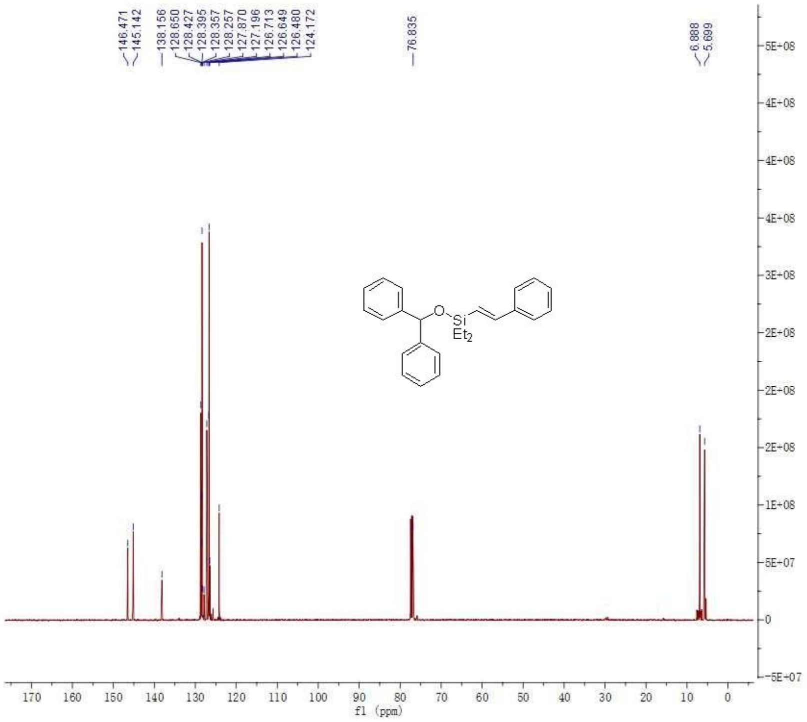

Parameter	Value
1 Title	xiaol_522
2 Origin	Bruker BioSpin GmbH
3 Spectrometer	Avance NEO 500
4 Solvent	CDCl3
5 Temperature	292.4
6 Pulse Sequence	zgpg30
7 Number of Scans	512
8 Receiver Gain	101
9 Relaxation Delay	2.0000
10 Pulse Width	10.0000
11 Acquisition Time	1.0879
12 Spectrometer Frequency	125.76
13 Spectral Width	30120.5
14 Lowest Frequency	-2484.0
15 Nucleus	13C
16 Acquired Size	32768
17 Spectral Size	32768

(E)-2-(2-((diethyl(styryl)silyl)oxy)ethyl)pyridine (4j)

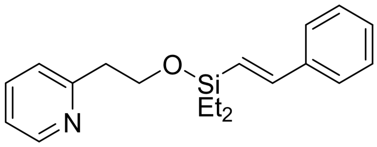

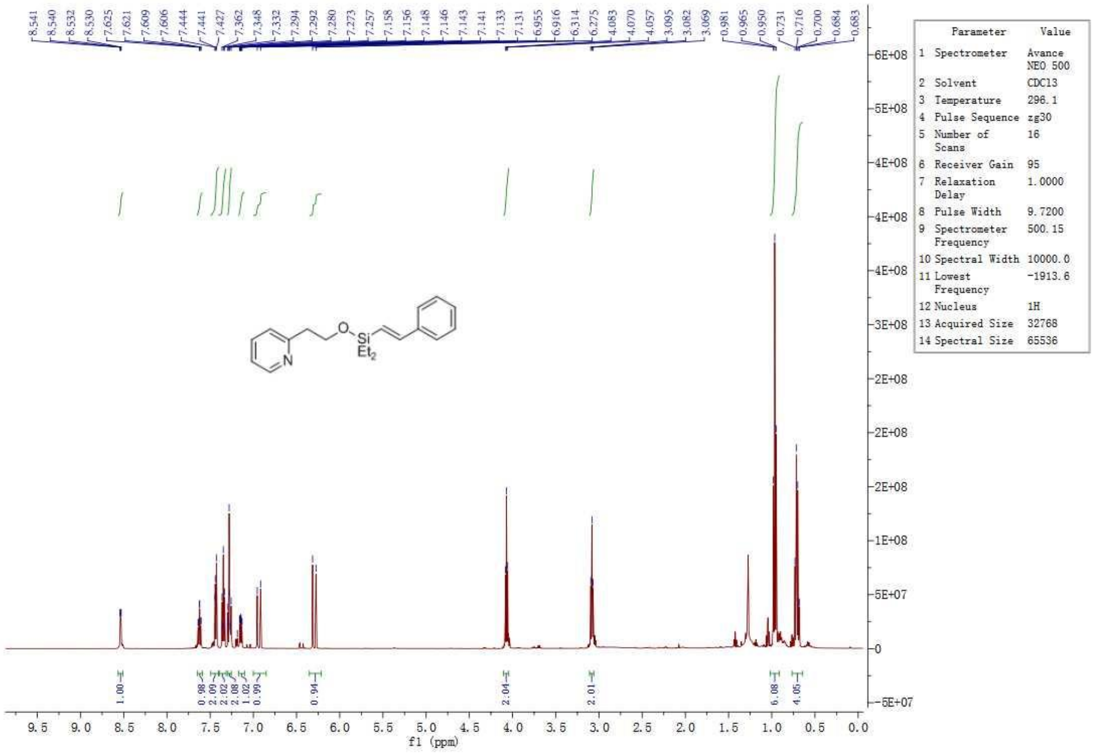

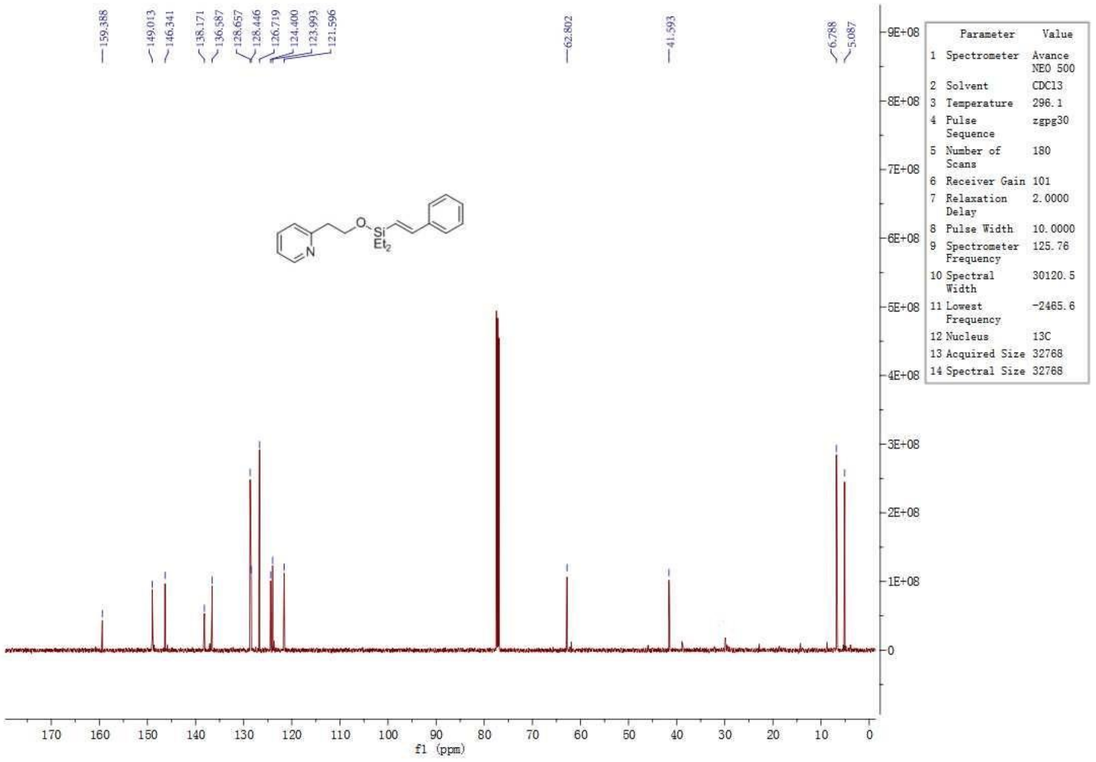

(E)-diethyl(1-(4-methoxyphenyl)ethoxy)(styryl)silane (5a)

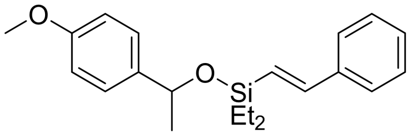

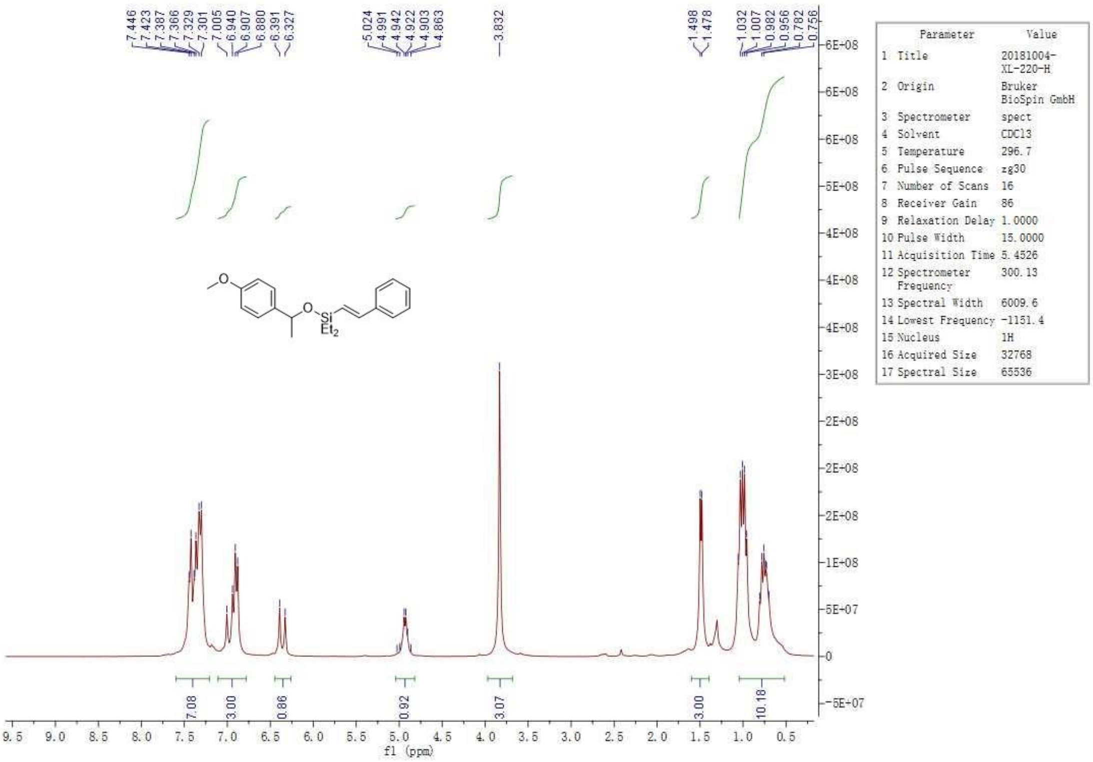

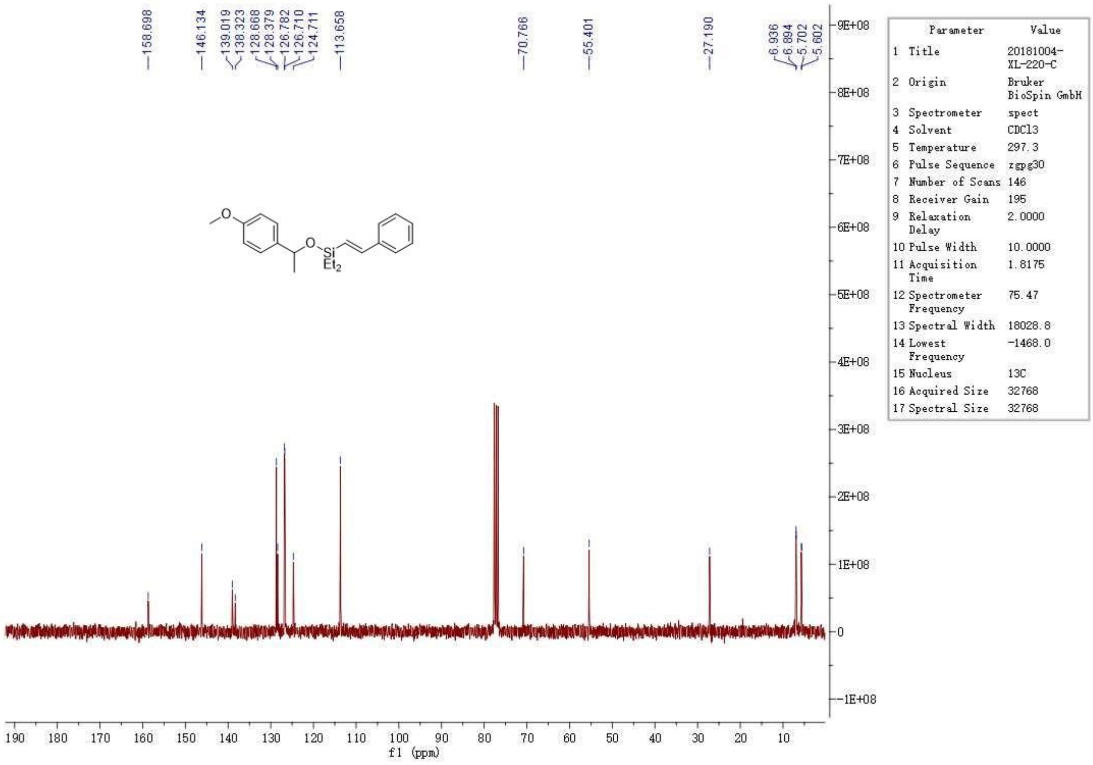

(E)-diethyl(1-(4-methoxyphenyl)ethoxy)(4-methoxystyryl)silane (5b)

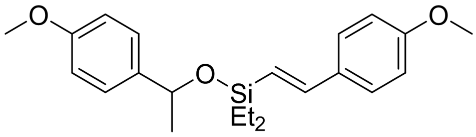

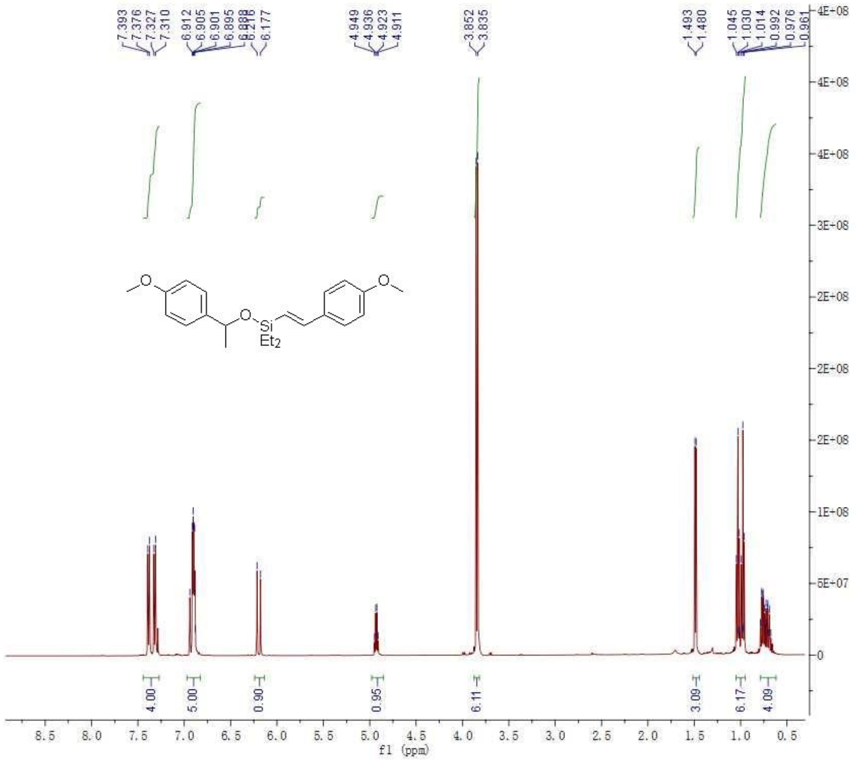

	Parameter	Value
1	Title	xiaol_255
2	Origin	Bruker BioSpin GmbH
3	Spectrometer	Avance NEO 500
4	Solvent	CDCl3
5	Temperature	291.6
6	Pulse Sequence	zg30
7	Number of Scans	16
8	Receiver Gain	32
9	Relaxation Delay	1.0000
10	Pulse Width	10.0000
11	Acquisition Time	3.2768
12	Spectrometer Frequency	500.15
13	Spectral Width	10000.0
14	Lowest Frequency	-1911.6
15	Nucleus	1H
16	Acquired Size	32768
17	Spectral Size	65536

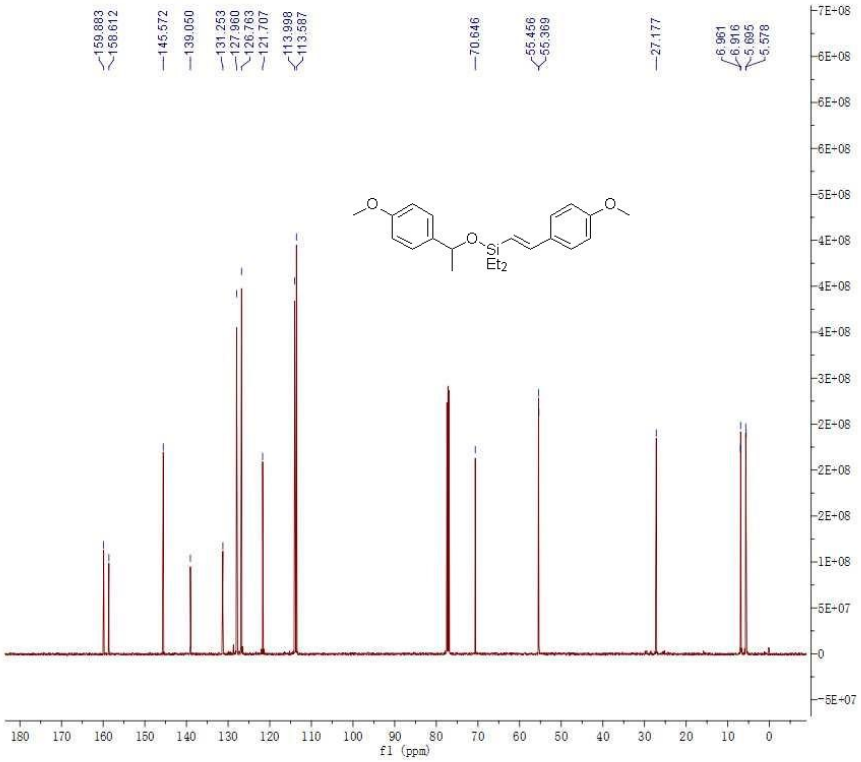

	Parameter	Value
1	Title	xiaol_255
2	Origin	Bruker BioSpin GmbH
3	Spectrometer	Avance NEO 500
4	Solvent	CDCl3
5	Temperature	292.6
6	Pulse Sequence	zgpg30
7	Number of Scans	512
8	Receiver Gain	101
9	Relaxation Delay	2.0000
10	Pulse Width	10.0000
11	Acquisition Time	1.0879
12	Spectrometer Frequency	125.76
13	Spectral Width	30120.5
14	Lowest Frequency	-2484.0
15	Nucleus	13C
16	Acquired Size	32768
17	Spectral Size	32768

(E)-diethyl(1-(4-methoxyphenyl)ethoxy)(4-methylstyryl)silane (5c)

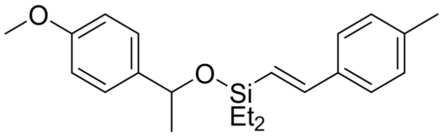

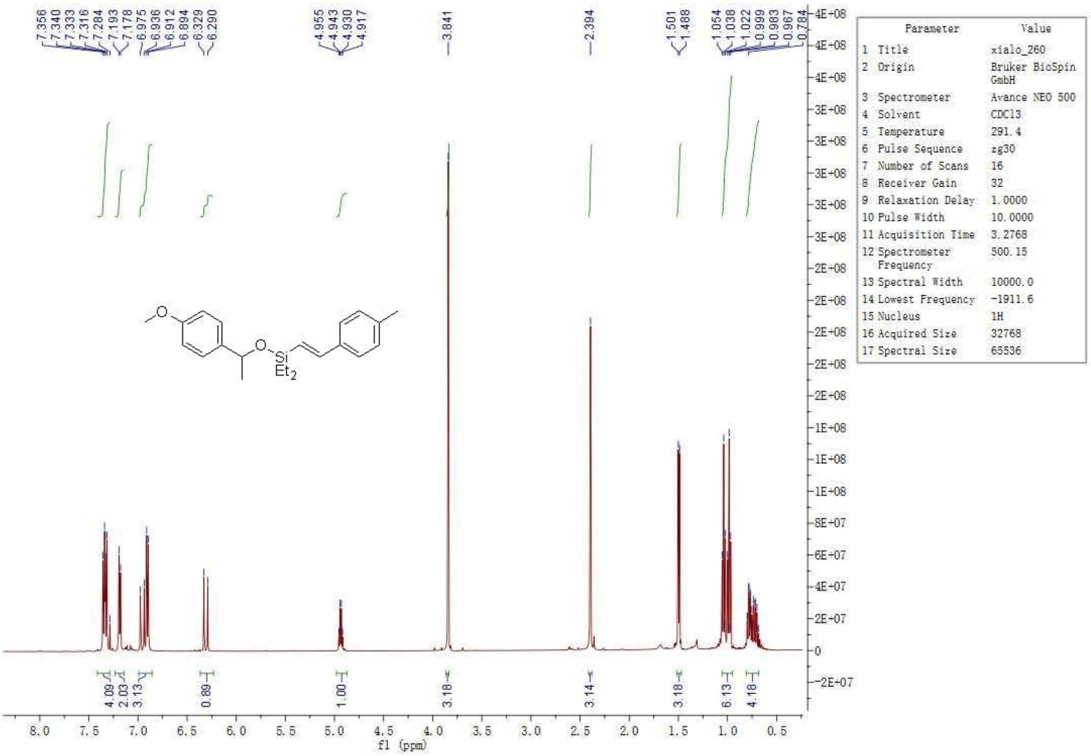

Parameter	Value
1 Title	xialo_260
2 Origin	Bruker BioSpin GmbH
3 Spectrometer	Avance NEO 500
4 Solvent	CDCl3
5 Temperature	291.4
6 Pulse Sequence	zg30
7 Number of Scans	16
8 Receiver Gain	32
9 Relaxation Delay	1.0000
10 Pulse Width	10.0000
11 Acquisition Time	3.2768
12 Spectrometer Frequency	500.15
13 Spectral Width	10000.0
14 Lowest Frequency	-1911.6
15 Nucleus	1H
16 Acquired Size	32768
17 Spectral Size	65536

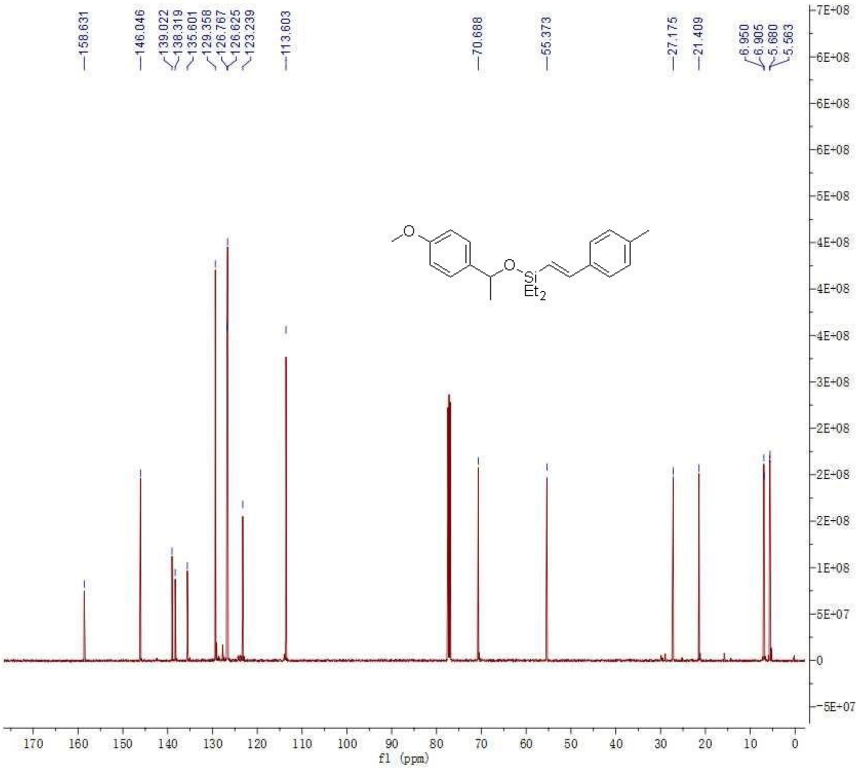

Parameter	Value
1 Title	xiaol_260-c
2 Origin	Bruker BioSpin GmbH
3 Spectrometer	Avance NEO 500
4 Solvent	CDCl3
5 Temperature	292.6
6 Pulse Sequence	zgpg30
7 Number of Scans	512
8 Receiver Gain	101
9 Relaxation Delay	2.0000
10 Pulse Width	10.0000
11 Acquisition Time	1.0879
12 Spectrometer Frequency	125.76
13 Spectral Width	30120.5
14 Lowest Frequency	-2484.0
15 Nucleus	13C
16 Acquired Size	32768
17 Spectral Size	32768

(E)-diethyl(1-(4-methoxyphenyl)ethoxy)(3-methylstyryl)silane (5d)

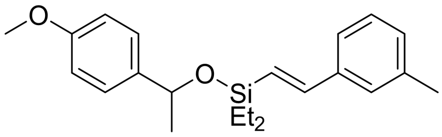

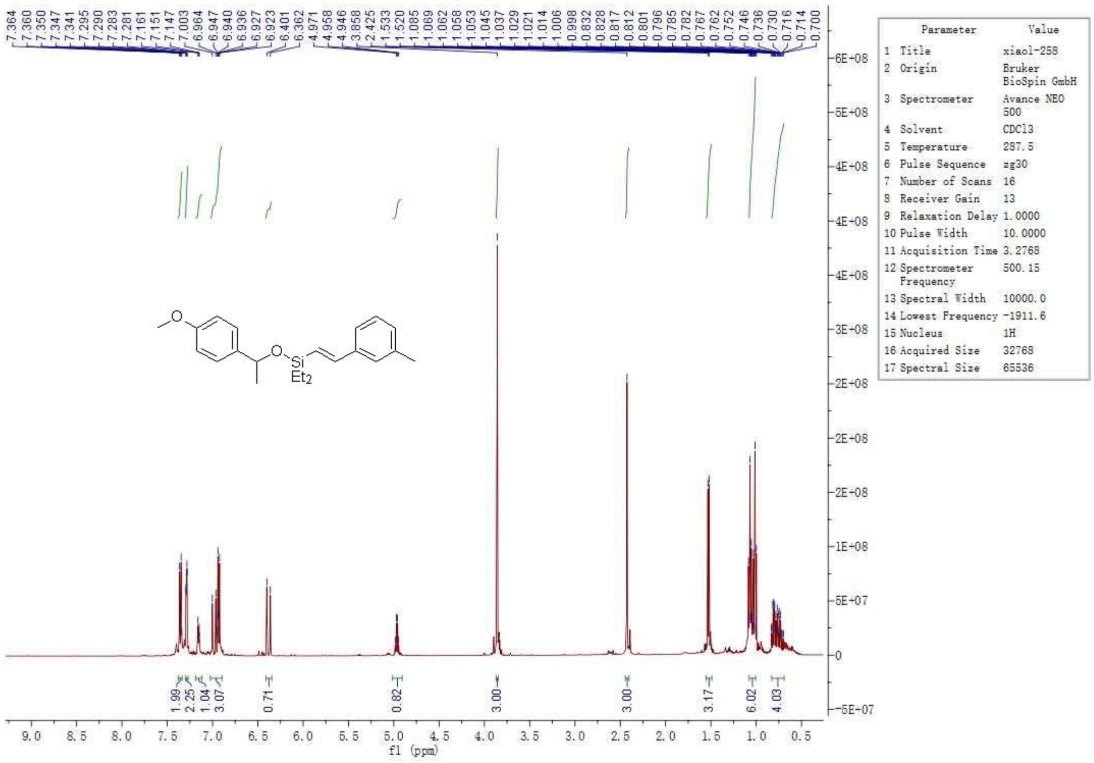

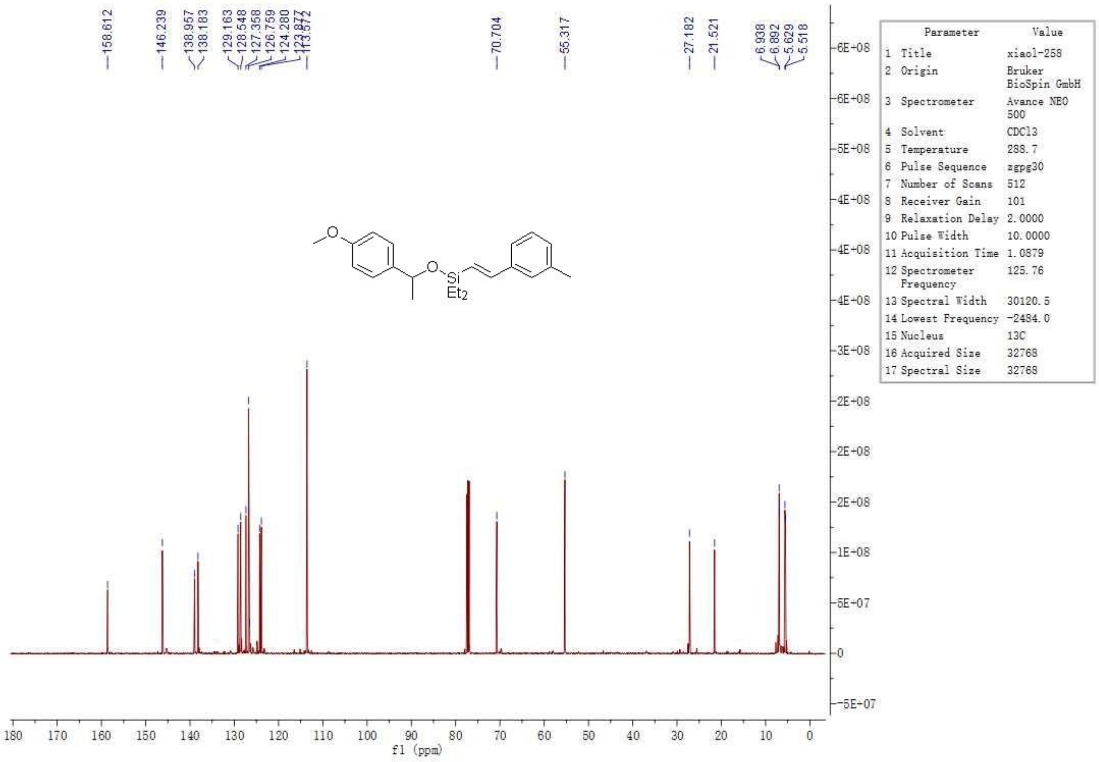

(E)-(4-(tert-butyl)styryl)diethyl(1-(4-methoxyphenyl)ethoxy)silane (5e)

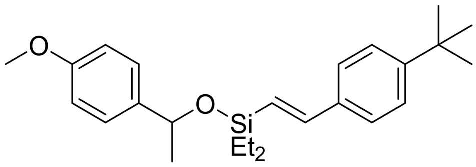

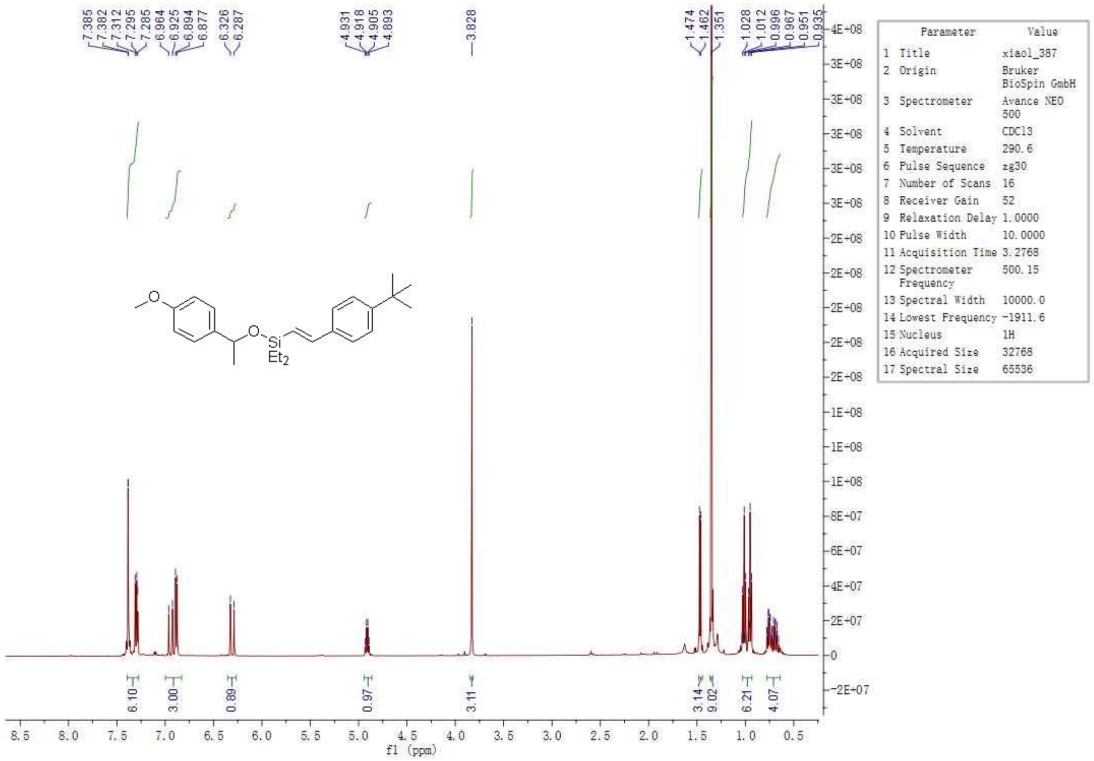

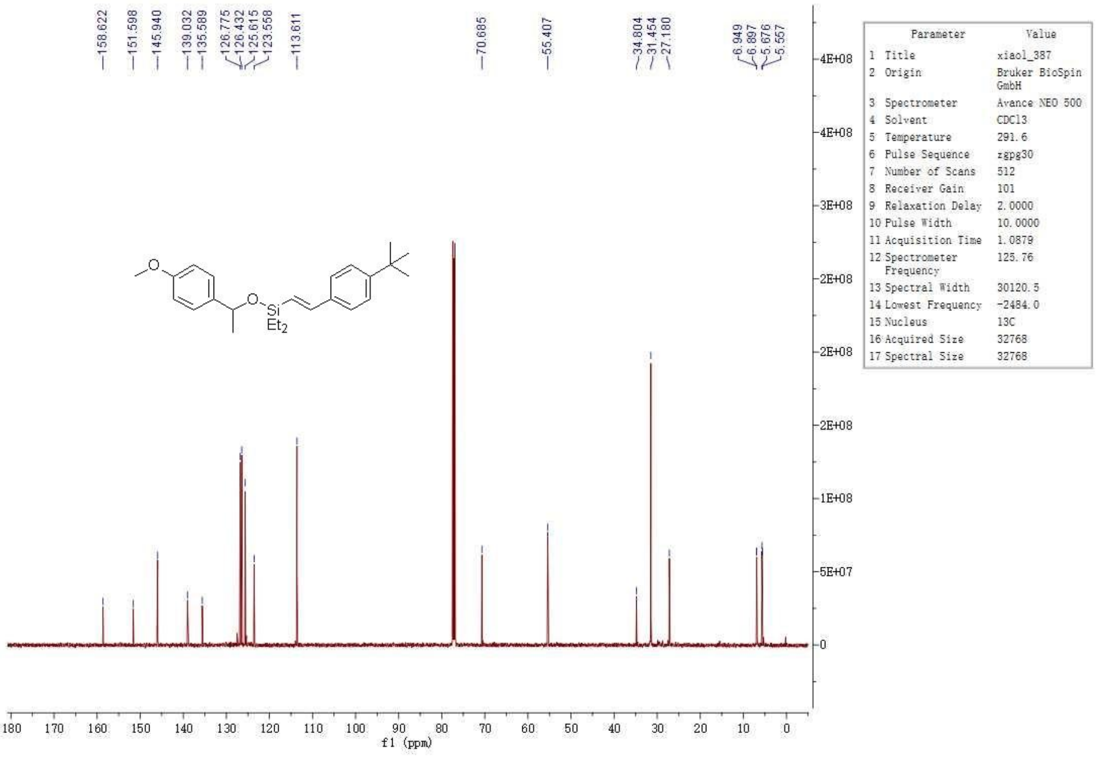

(E)-diethyl(4-fluorostyryl)(1-(4-methoxyphenyl)ethoxy)silane (5f)

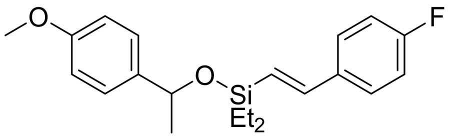

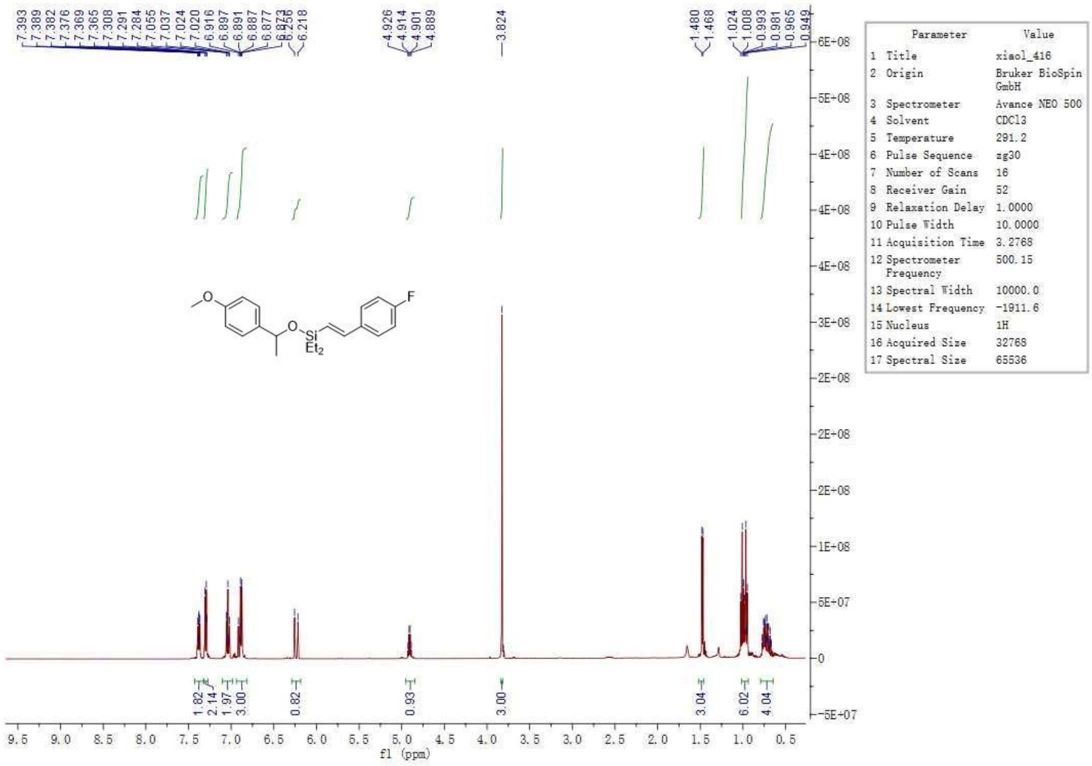

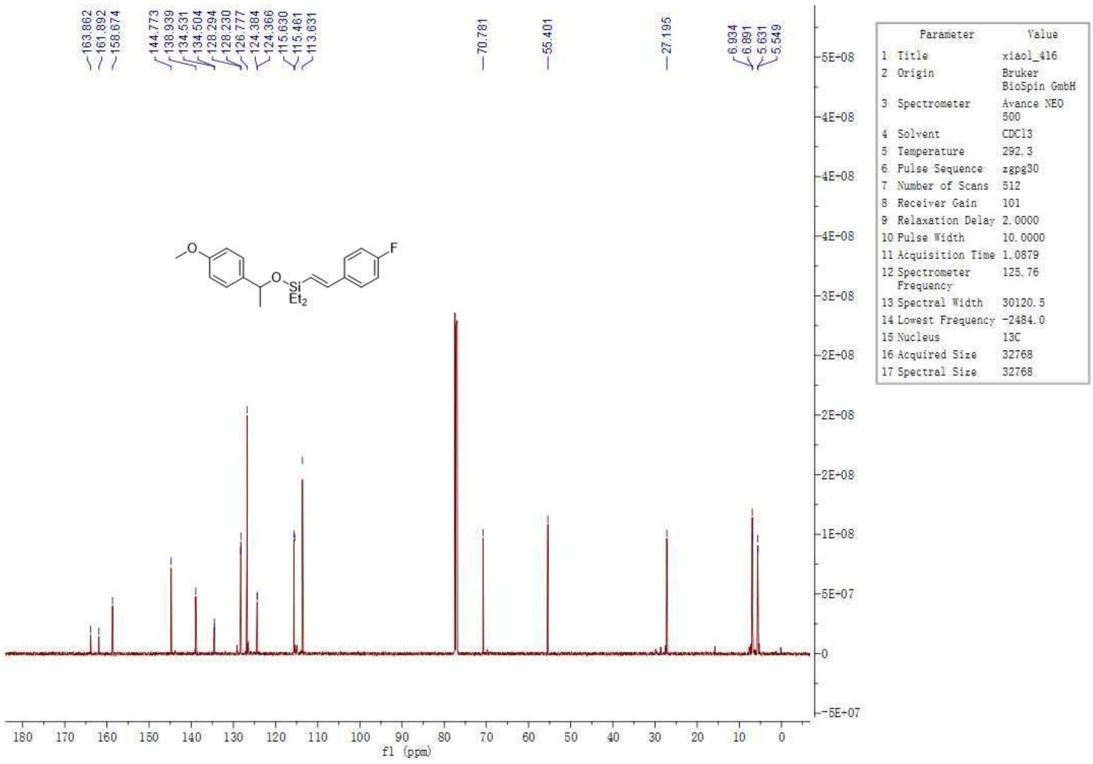

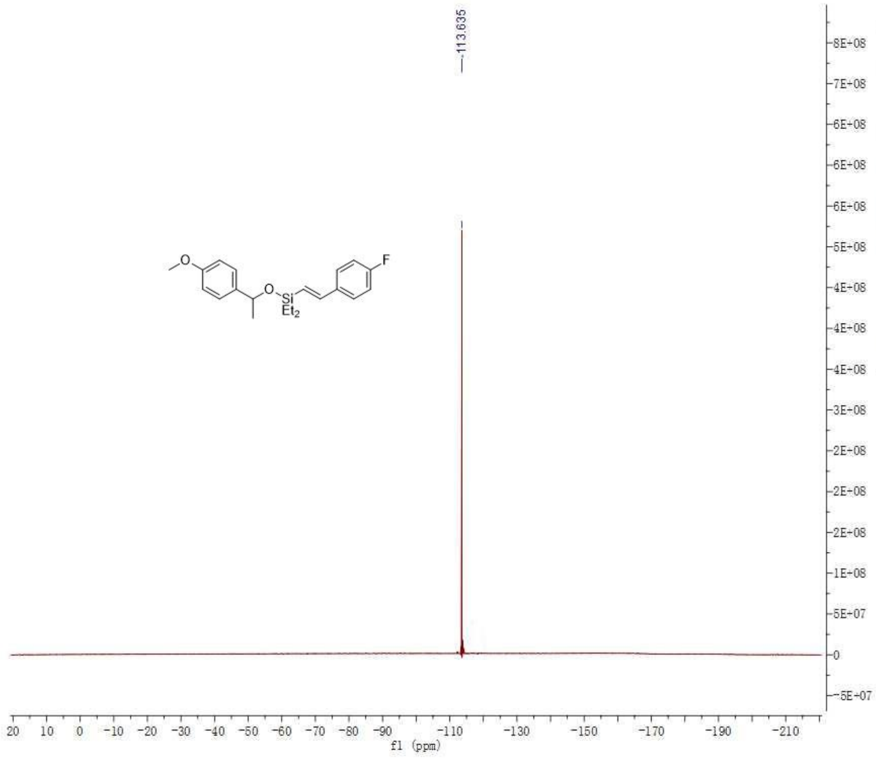

	Parameter	Value
1	Title	xiaol_416-f
2	Origin	Bruker BioSpin GmbH
3	Spectrometer	Avance NEO 500
4	Solvent	CDCl3
5	Temperature	291.6
6	Pulse Sequence	zgig
7	Number of Scans	16
8	Receiver Gain	101
9	Relaxation Delay	1.0000
10	Pulse Width	15.0000
11	Acquisition Time	0.5767
12	Spectrometer Frequency	470.61
13	Spectral Width	113636.4
14	Lowest Frequency	-103879.3
15	Nucleus	19F
16	Acquired Size	65536
17	Spectral Size	65536

(E)-(4-chlorostyryl)diethyl(1-(4-methoxyphenyl)ethoxy)silane (5g)

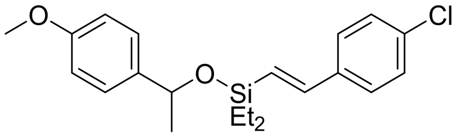

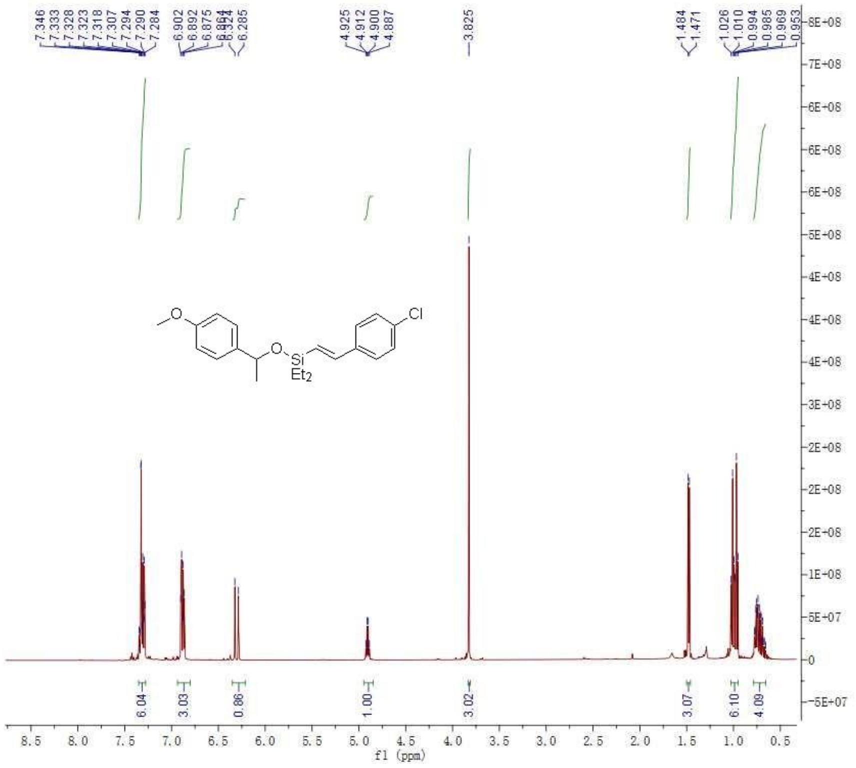

Parameter	Value
1 Title	xiaol_257
2 Origin	Bruker BioSpin GmbH
3 Spectrometer	Avance NEO 500
4 Solvent	CDCl3
5 Temperature	290.5
6 Pulse Sequence	zg30
7 Number of Scans	16
8 Receiver Gain	32
9 Relaxation Delay	1.0000
10 Pulse Width	10.0000
11 Acquisition Time	3.2768
12 Spectrometer Frequency	500.15
13 Spectral Width	10000.0
14 Lowest Frequency	-1911.6
15 Nucleus	1H
16 Acquired Size	32768
17 Spectral Size	65536

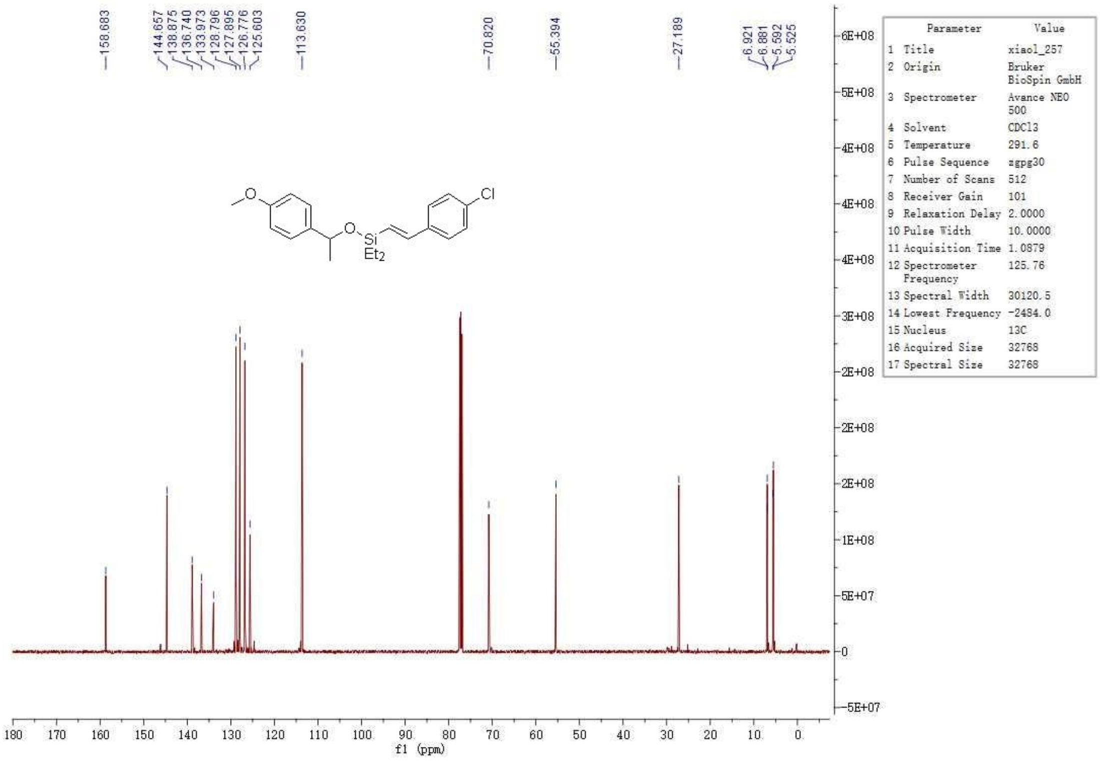

Parameter	Value
1 Title	xiaol_257
2 Origin	Bruker BioSpin GmbH
3 Spectrometer	Avance NEO 500
4 Solvent	CDCl3
5 Temperature	291.6
6 Pulse Sequence	zgpg30
7 Number of Scans	512
8 Receiver Gain	101
9 Relaxation Delay	2.0000
10 Pulse Width	10.0000
11 Acquisition Time	1.0879
12 Spectrometer Frequency	125.76
13 Spectral Width	30120.5
14 Lowest Frequency	-2484.0
15 Nucleus	13C
16 Acquired Size	32768
17 Spectral Size	32768

(E)-(4-bromostyryl)diethyl(1-(4-methoxyphenyl)ethoxy)silane (5h)

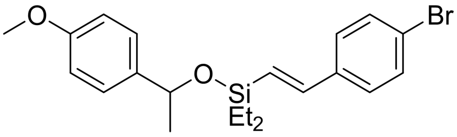

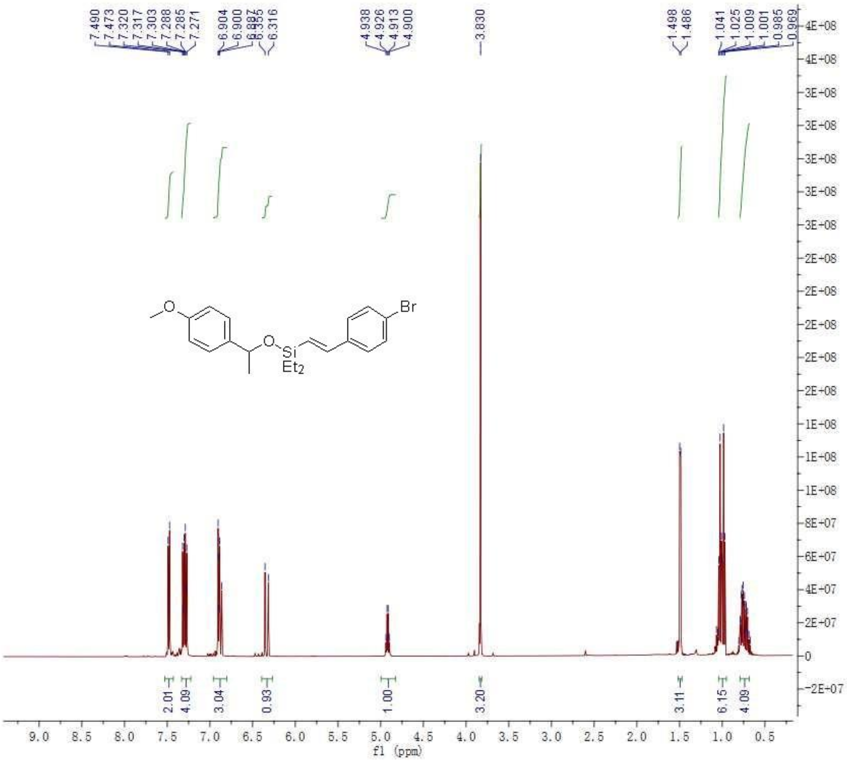

Parameter	Value
1 Title	xiaol_256
2 Origin	Bruker BioSpin GmbH
3 Spectrometer	Avance NEO 500
4 Solvent	CDCl3
5 Temperature	291.5
6 Pulse Sequence	zg30
7 Number of Scans	16
8 Receiver Gain	20
9 Relaxation Delay	1.0000
10 Pulse Width	10.0000
11 Acquisition Time	3.2768
12 Spectrometer Frequency	500.15
13 Spectral Width	10000.0
14 Lowest Frequency	-1911.6
15 Nucleus	1H
16 Acquired Size	32768
17 Spectral Size	65536

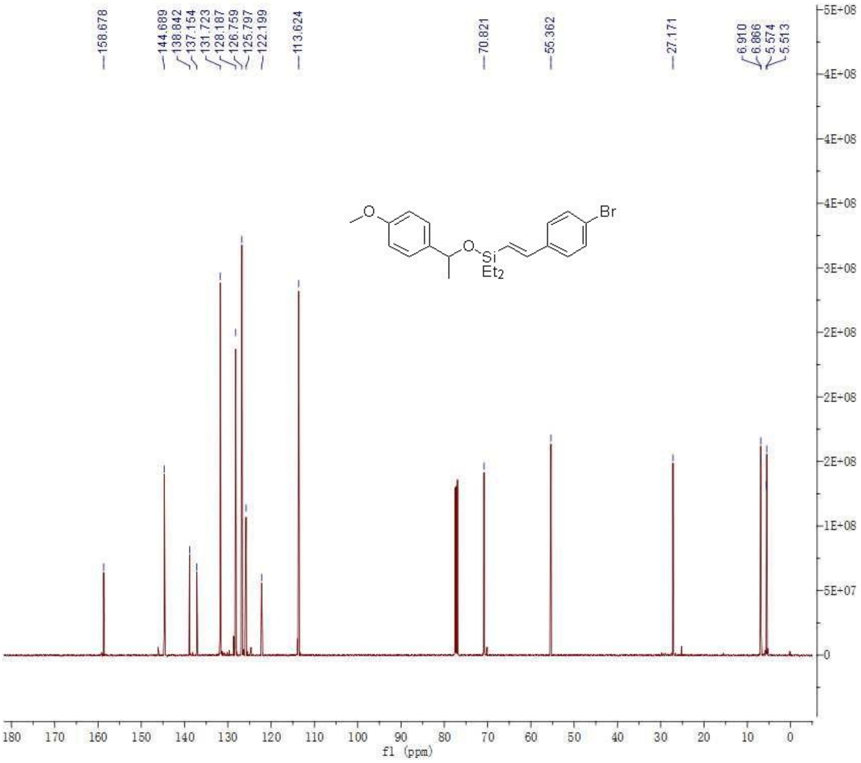

Parameter	Value
1 Title	xiaol_256
2 Origin	Bruker BioSpin GmbH
3 Spectrometer	Avance NEO 500
4 Solvent	CDCl3
5 Temperature	292.6
6 Pulse Sequence	zgpg30
7 Number of Scans	512
8 Receiver Gain	101
9 Relaxation Delay	2.0000
10 Pulse Width	10.0000
11 Acquisition Time	1.0879
12 Spectrometer Frequency	125.76
13 Spectral Width	30120.5
14 Lowest Frequency	-2484.0
15 Nucleus	13C
16 Acquired Size	32768
17 Spectral Size	32768

(E)-4-(2-(diethyl(1-(4-methoxyphenyl)ethoxy)silyl)vinyl)pyridine (5i)

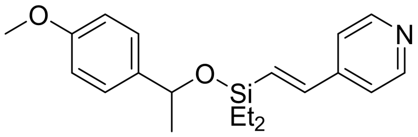

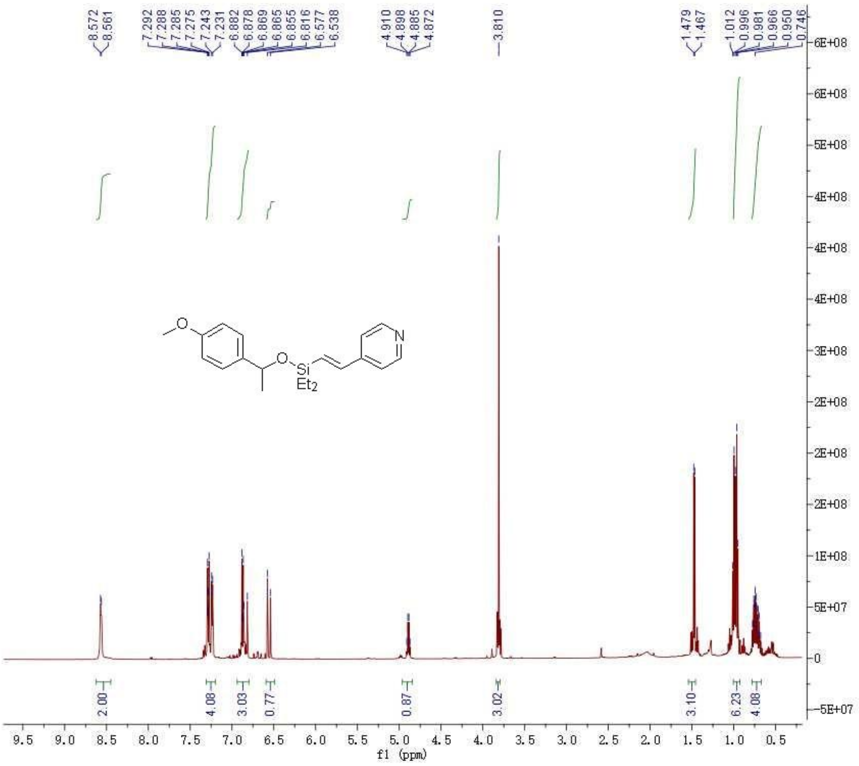

Parameter	Value
1 Title	xiaOL_423
2 Origin	Bruker BioSpin GmbH
3 Spectrometer	Avance NEO 500
4 Solvent	CDCl3
5 Temperature	291.2
6 Pulse Sequence	zg30
7 Number of Scans	16
8 Receiver Gain	57
9 Relaxation Delay	1.0000
10 Pulse Width	10.0000
11 Acquisition Time	3.2768
12 Spectrometer Frequency	500.15
13 Spectral Width	10000.0
14 Lowest Frequency	-1911.6
15 Nucleus	1H
16 Acquired Size	32768
17 Spectral Size	65536

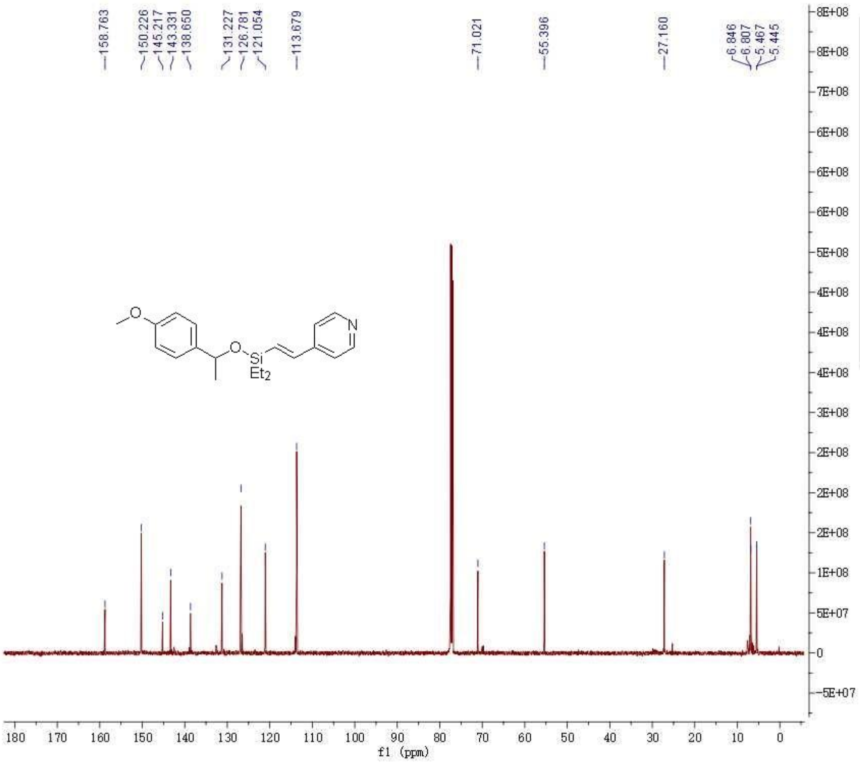

Parameter	Value
1 Title	xiaOL_423
2 Origin	Bruker BioSpin GmbH
3 Spectrometer	Avance NEO 500
4 Solvent	CDCl3
5 Temperature	292.4
6 Pulse Sequence	zgpg30
7 Number of Scans	512
8 Receiver Gain	101
9 Relaxation Delay	2.0000
10 Pulse Width	10.0000
11 Acquisition Time	1.0879
12 Spectrometer Frequency	125.76
13 Spectral Width	30120.5
14 Lowest Frequency	-2484.0
15 Nucleus	13C
16 Acquired Size	32768
17 Spectral Size	32768

2,2-diethyl-2H-naphtho[1,8-cd][1,2]oxasilole (7a)

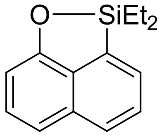

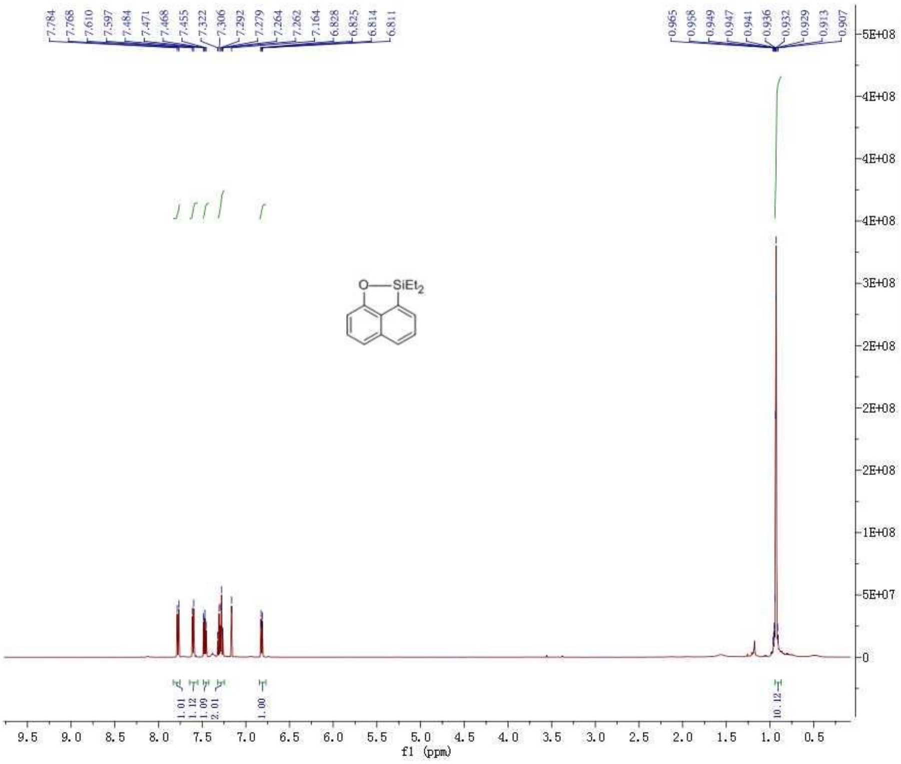

Parameter	Value
1 Spectrometer	Avance NEO 500
2 Solvent	CDCl3
3 Temperature	296.1
4 Pulse Sequence	zg30
5 Number of Scans	16
6 Receiver Gain	101
7 Relaxation Delay	1.0000
8 Pulse Width	9.7200
9 Spectrometer Frequency	500.15
10 Spectral Width	10000.0
11 Lowest Frequency	-1971.6
12 Nucleus	1H
13 Acquired Size	32768
14 Spectral Size	65536

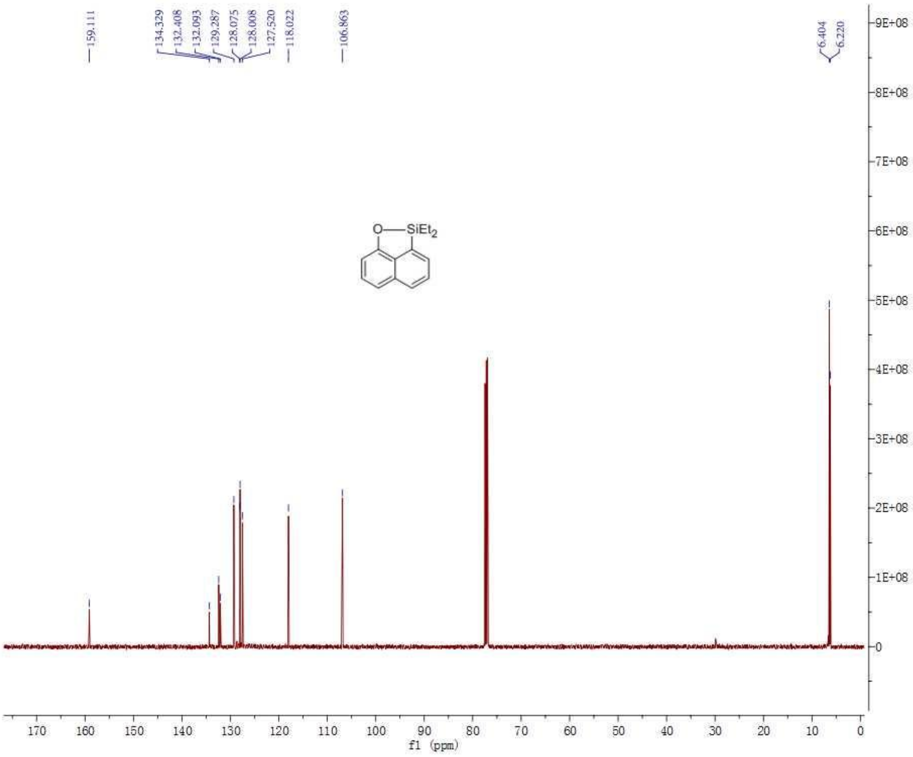

Parameter	Value
1 Spectrometer	Avance NEO 500
2 Solvent	CDCl3
3 Temperature	296.2
4 Pulse Sequence	zgpg30
5 Number of Scans	200
6 Receiver Gain	101
7 Relaxation Delay	2.0000
8 Pulse Width	10.0000
9 Spectrometer Frequency	125.76
10 Spectral Width	30120.5
11 Lowest Frequency	-2465.2
12 Nucleus	13C
13 Acquired Size	32768
14 Spectral Size	32768

2,2-diethyl-6-methoxy-2H-naphtho[1,8-cd][1,2]oxasilole (7b)

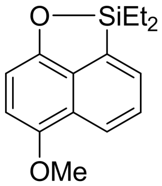

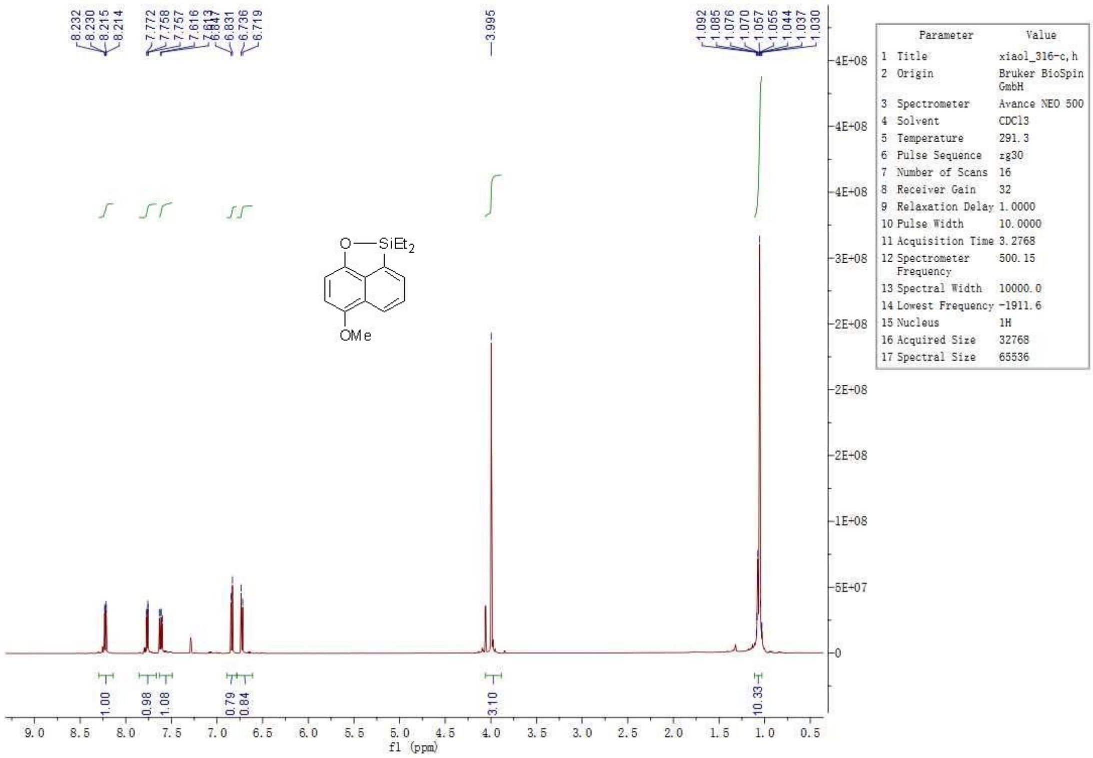

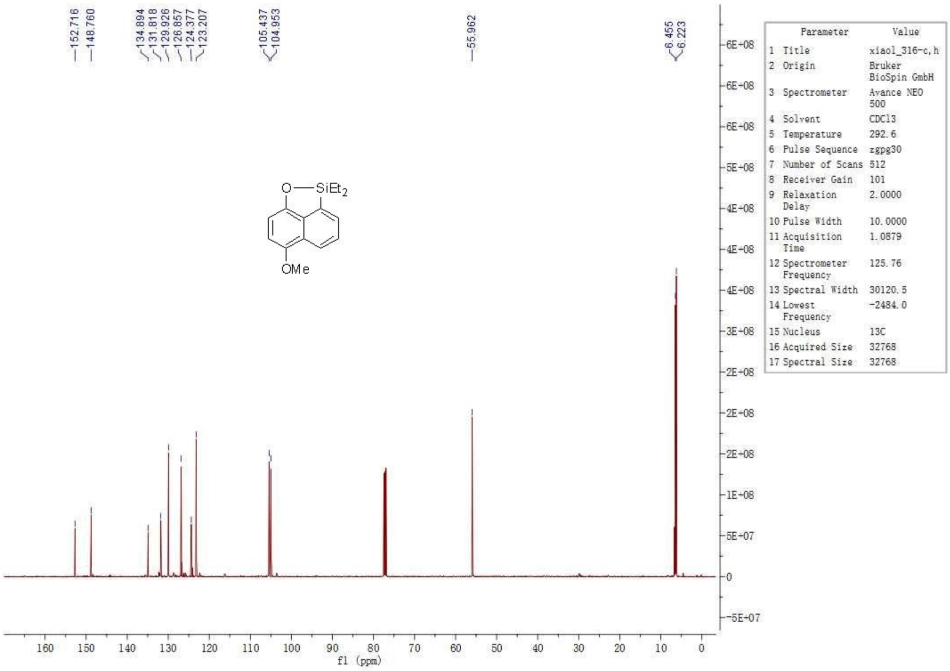

6-chloro-2,2-diethyl-2H-naphtho[1,8-cd][1,2]oxasilole (7c)

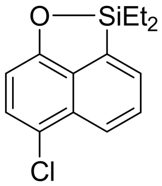

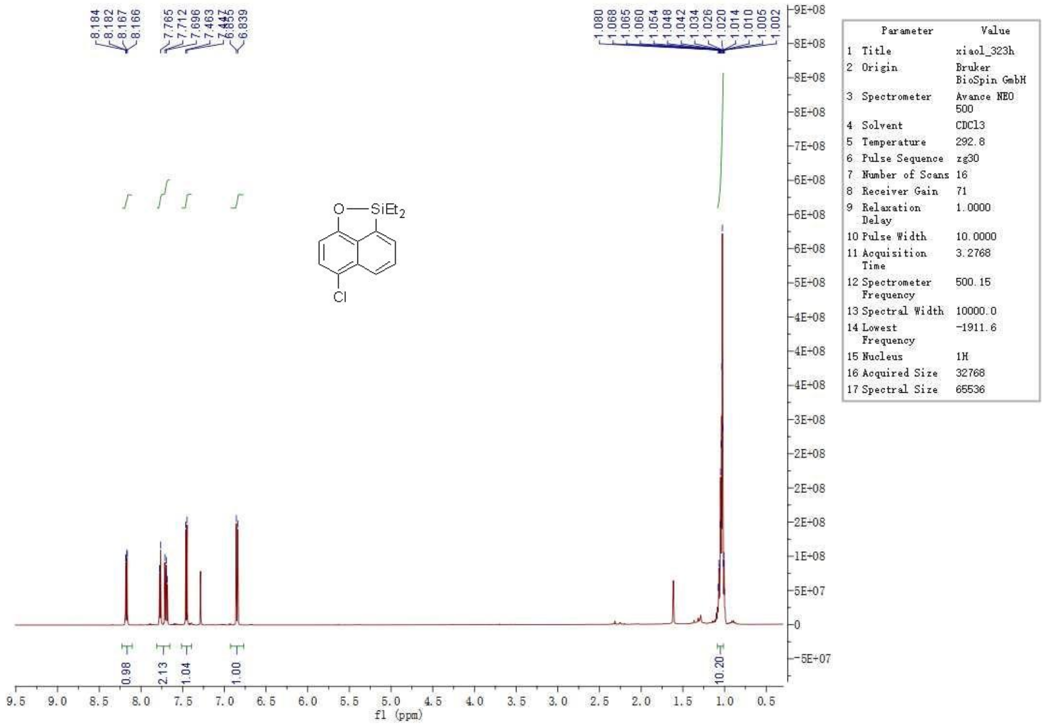

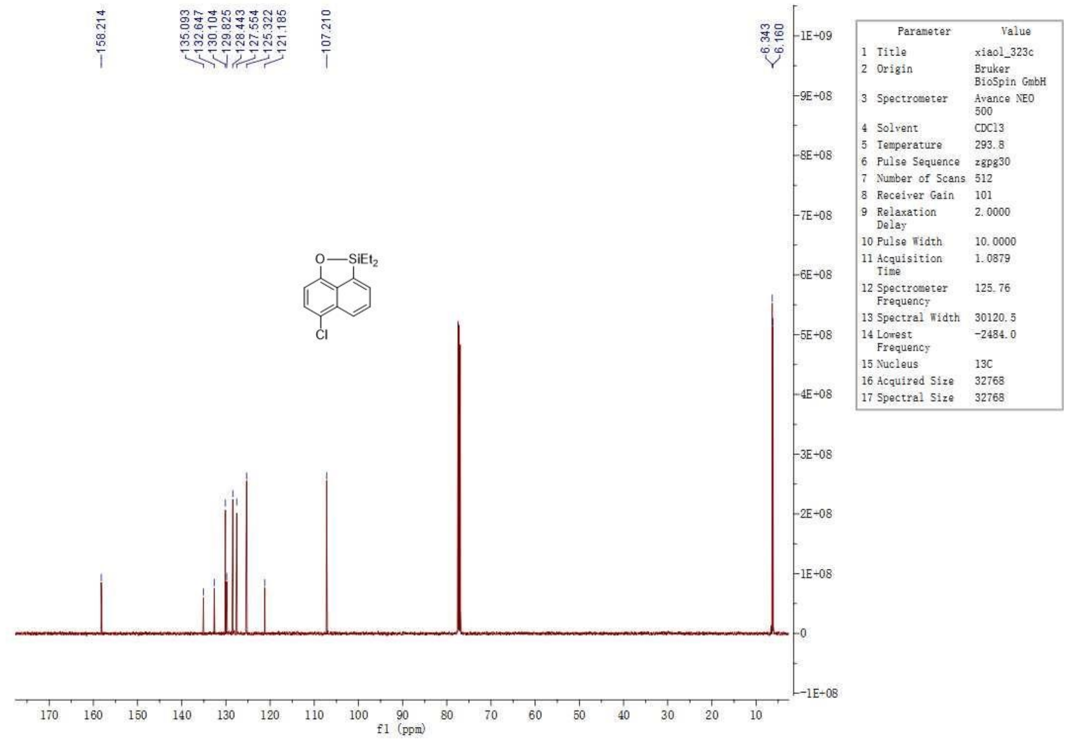

6-bromo-2,2-diethyl-2H-naphtho[1,8-cd][1,2]oxasilole (7d)

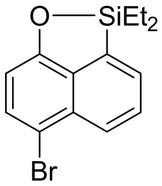

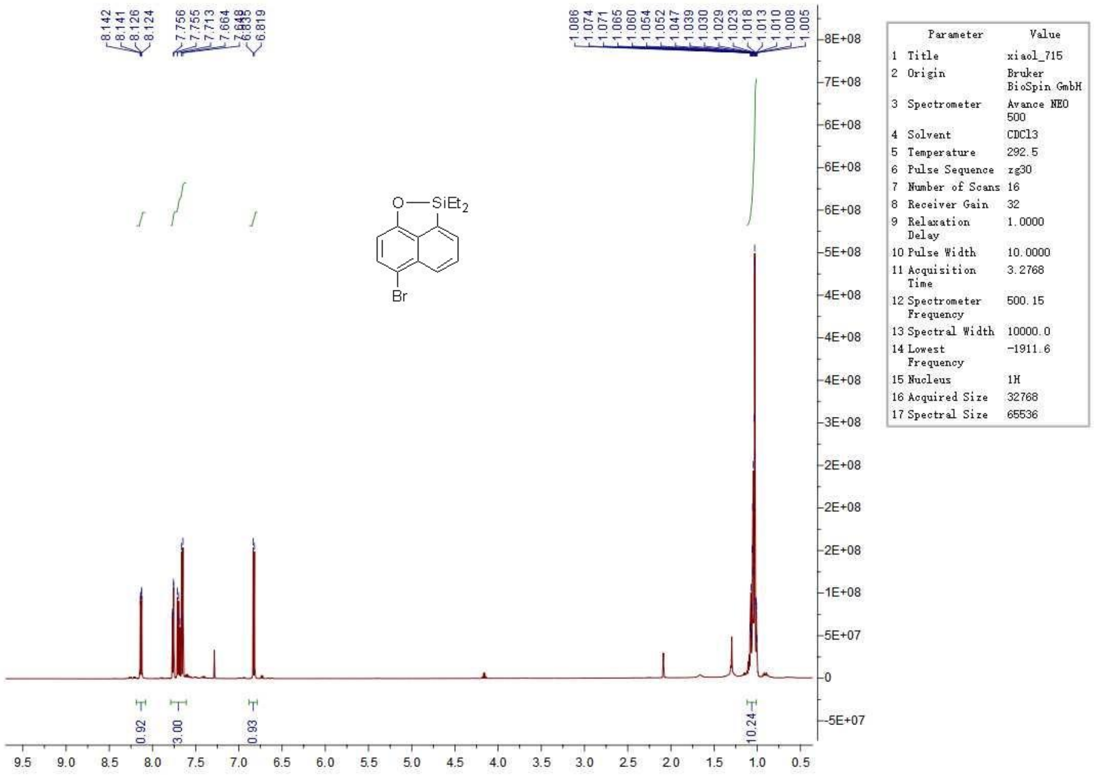

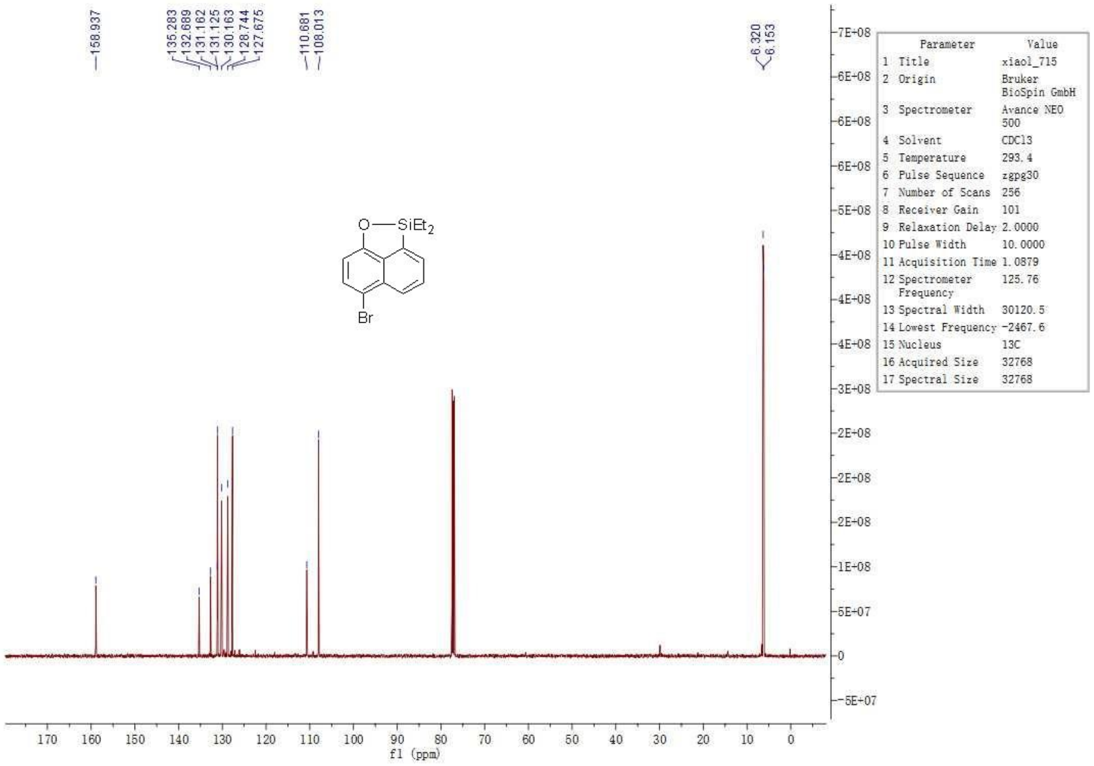

4,4-diethyl-4H-pyreno[10,1-cd][1,2]oxasilole (7e)

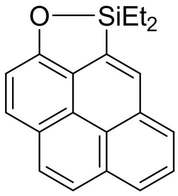

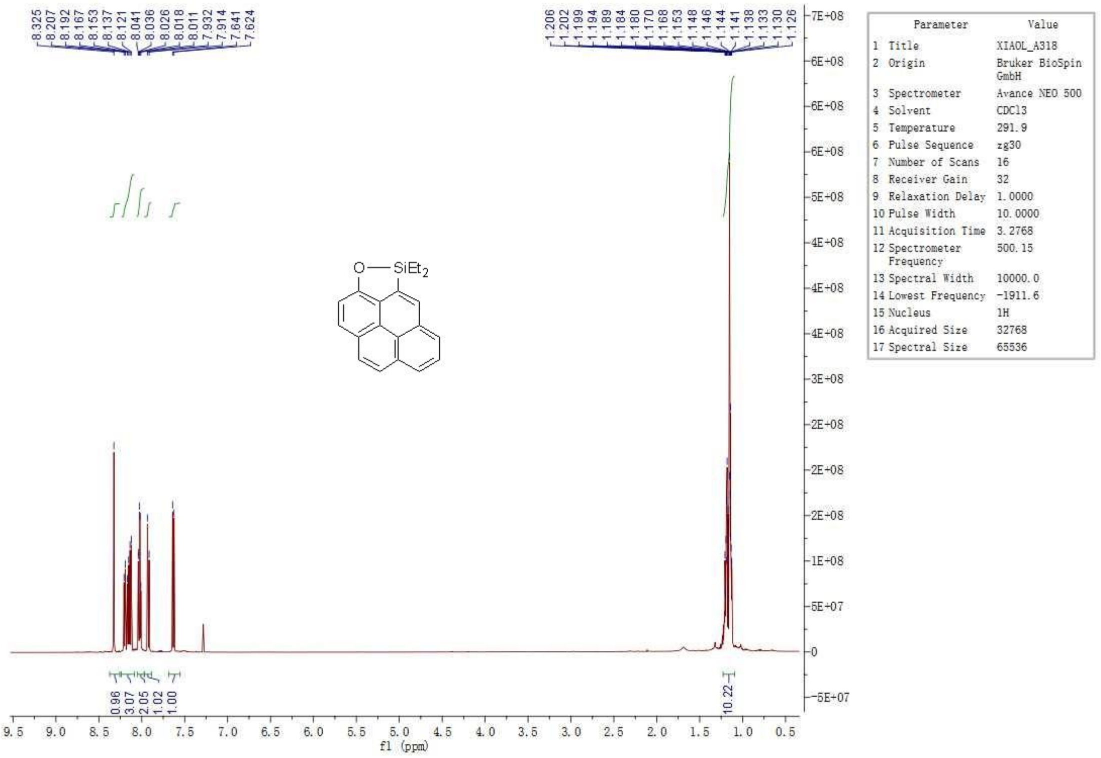

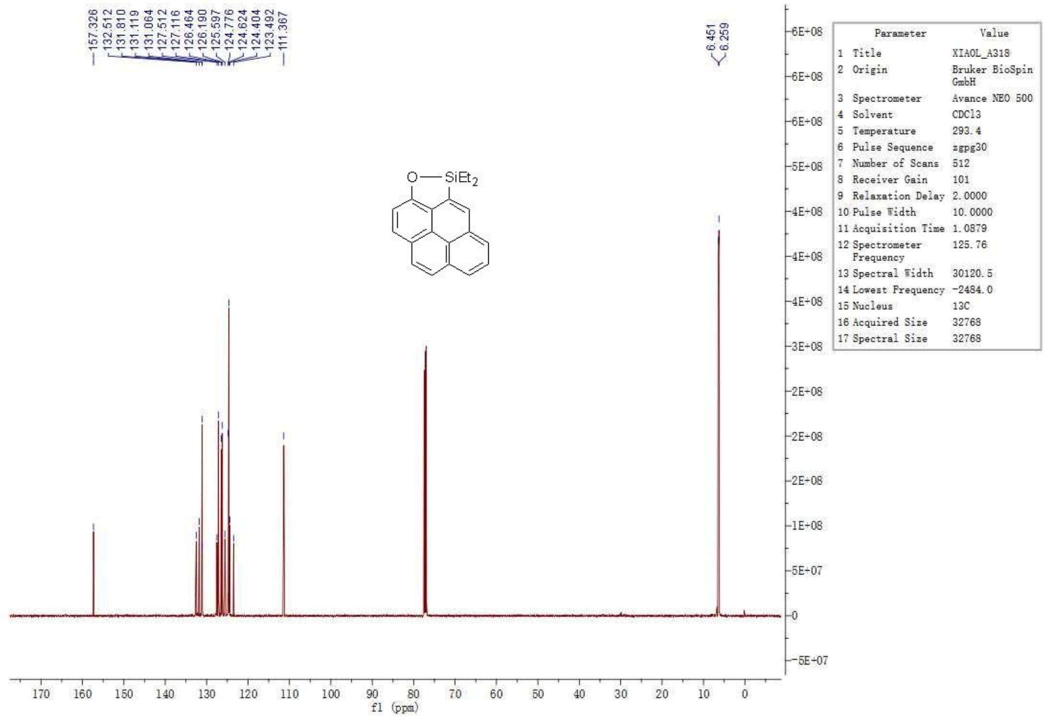

3,3-diethyl-1,3-dihydronaphtho[2,1-c][1,2]oxasilole (9a)

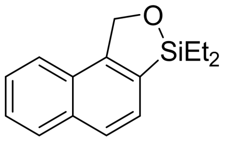

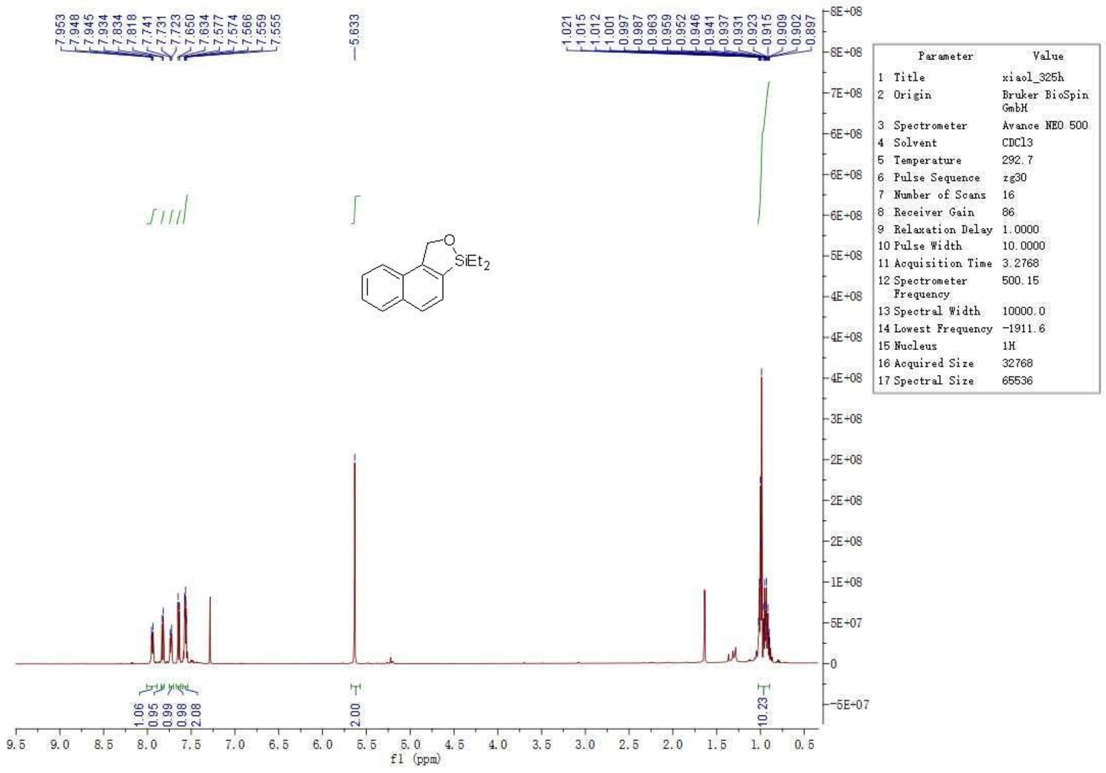

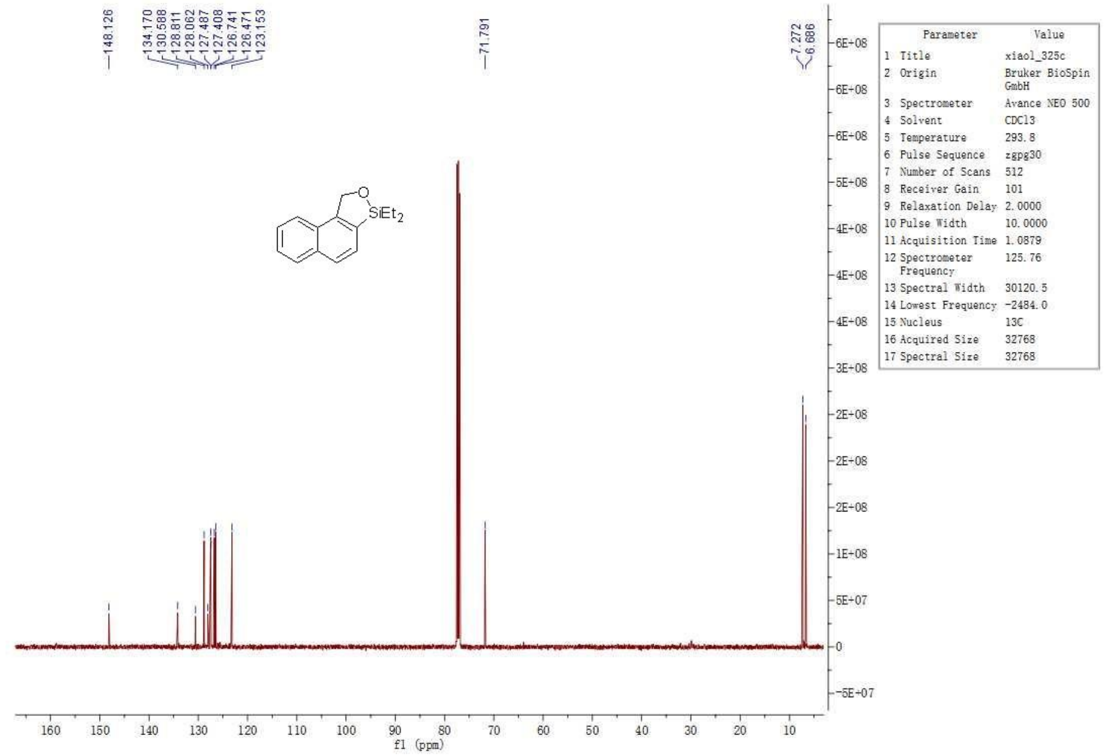

Supplement: Supplementary file 1 [file molecules-28-07186-s001.zip › molecules-2632183-supplementary.pdf]
